# Crossing the Solubility Rubicon: 15-Crown-5 Facilitates the Preparation of Water-Soluble Sulfo-NHS Esters in Organic Solvents

**DOI:** 10.1021/acs.bioconjchem.3c00396

**Published:** 2023-12-12

**Authors:** Nicholas D. J. Yates, Connor G. Miles, Christopher D. Spicer, Martin A. Fascione, Alison Parkin

**Affiliations:** † Department of Chemistry, University of York, York, North Yorkshire YO10 5DD, United Kingdom

## Abstract

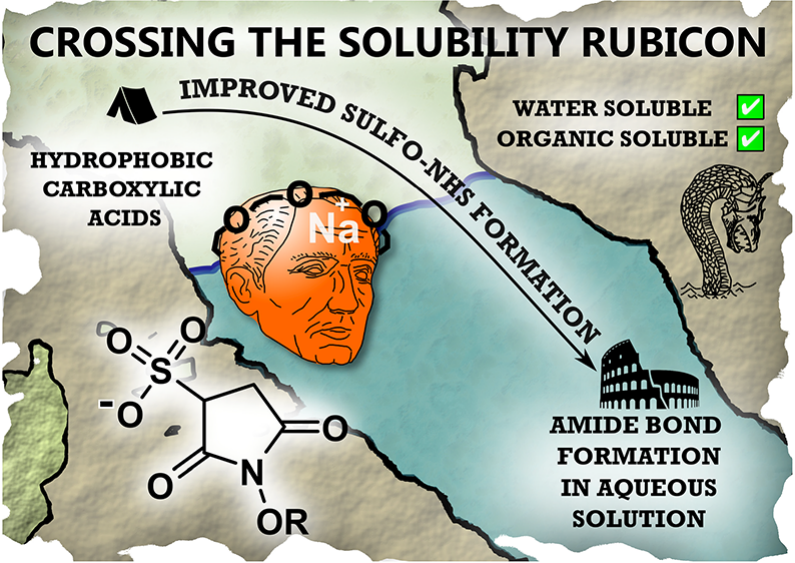

The Sulfo-NHS ester is a mainstay reagent for facilitating amide bond formation between carboxylic acids and amine functionalities in water. However, the preparation of Sulfo-NHS esters currently requires hydrophobic carboxylic acids, which are poorly water-soluble, to first be reacted with the *N*-hydroxysulfosuccinimide sodium salt, which is insoluble in organic solvents. The mutually incompatible solvation requirements thus complicate the synthesis of Sulfo-NHS esters. As a simple, rapid, and cost-effective solution to this problem, we report that the use of 15-crown-5 to complex the sodium cation of *N*-hydroxysulfosuccinimide sodium salt circumnavigates these solvation incompatibility issues by rendering the *N*-hydroxysulfosuccinimide salt soluble in organic solvents, resulting in a cleaner esterification reaction and thus improved yields of activated ester product. We also demonstrate that the resultant “crowned” Sulfo-NHS-ester remains water-soluble and is no less reactive than its classic “uncrowned” Sulfo-NHS counterpart when used in bioconjugation reactions between protein amine-functionalities and hydrophobic carboxylic acids.

The ubiquitous amide bond plays critical roles in biology and, by extension, pharmacology.^1,2^ Indeed, amide bond formation has been the most commonly performed reaction in the pharmaceutical industry for several decades, and amide bonds occur in over half of the target compounds in medicinal chemistry patents.^1^ The properties of amide bonds that allow them to form the strong backbones of proteins also makes them ideal for creating many man-made materials, ranging from hydrogels to nylon.^2^

Due to the poor ability of OH to serve as a leaving group, the direct thermal condensation of an amide and a carboxylic acid requires harsh forcing conditions which are unsuitable for the creation of many pharmaceuticals.^2−4^ Born of this need, a variety of carboxylic acid activation chemistries have been developed that facilitate amide bond formation at mild temperatures. Of all these methods, the activation of carboxylic acids via esterification with *N*-hydroxysuccinimide (NHS) **1** is the most popular for facilitating amide bond formation when preparing a range of bioconjugates (Scheme 1).^5^

**Scheme 1:**
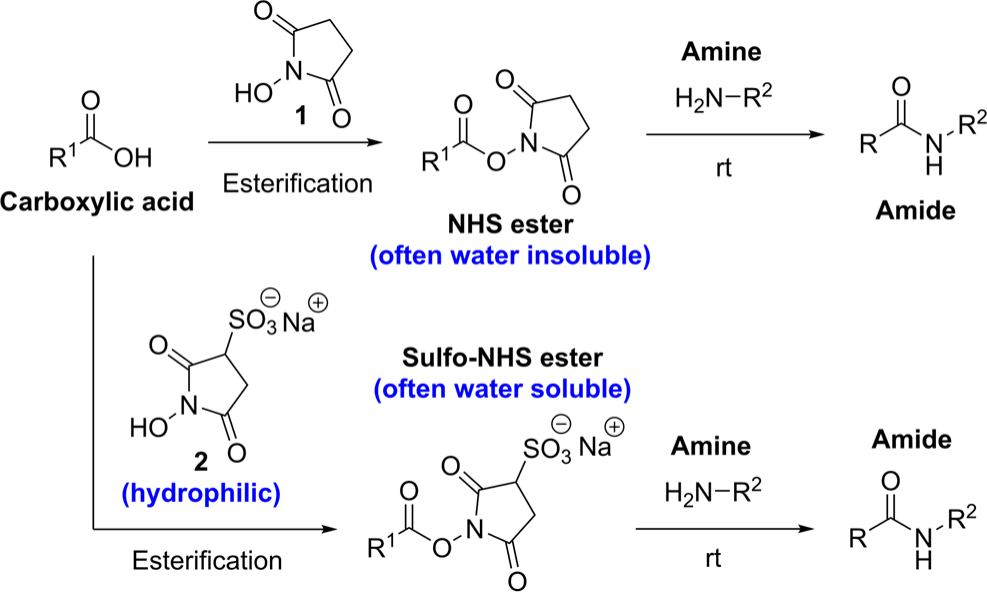
Synthesis of Amide Bonds via Reaction with NHS Esters Derived from **1** and Sulfo-NHS Esters Derived from **2**

While NHS itself is highly water-soluble, NHS esters of nonpolar carboxylic acids are frequently insoluble in water.^6,7^ To overcome these limitations, a more polar *N*-hydroxysuccinimide-themed alcohol was invented – *N*-hydroxysulfosuccinimide sodium salt (“Sulfo-NHS”) **2**, Scheme 1; this is commercially available and has become a mainstay reagent in bioconjugation chemistry.^8,9^ The charged sulfonate group enhances the solubility of Sulfo-NHS esters in aqueous solution; this is useful in applications where the equivalent “plain” NHS-ester is poorly water-soluble,^5−8,10,11^ as is often the case when preparing antibody-drug conjugates^12^ or when appending fluorophores onto biomolecules.^13^ The ionic nature of Sulfo-NHS esters also typically renders them membrane impermeable, making them well-suited to cell-surface labeling applications.^5,8^ However, the preparation of Sulfo-NHS esters relies on the reaction of the carboxylic acid with commercially available **2**, which is highly soluble in water but very poorly soluble in organic solvents.^6^ Experimentalists therefore have to contend with a fundamental solvation incompatibility when attempting to generate Sulfo-NHS esters from poorly water-soluble carboxylic acids (such as the extended conjugated pi-systems frequently encountered in high quantum-yield fluorescent labels). A secondary issue is that **2** is susceptible to hydrolysis, which precludes the use of water/organic co-solvent mixtures for extended periods of time. As such, while **2** is a popular reagent suitable for preparing a wealth of Sulfo-NHS ester products, in certain cases the different solvent preferences of **2** and its intended carboxylic acid partners can lead to diminished yields of Sulfo-NHS ester products, or can necessitate that Sulfo-NHS esters are prepared *in situ* in aqueous solution and used crude.^6^

We hypothesized that **2** (and esters derived thereof) could be rendered more soluble in organic solvents, while not suffering greatly diminished water solubility, by coordinating the Na^+^ cation with 15-crown-5. To this end, *N*-hydroxysulfosuccinimide [Na(15-Crown-5)] salt **3** (referred to as “C-Sulfo-NHS”) was prepared in a one-step methodology from **2** via mixing with one equivalent of 15-crown-5 in methanol, followed by concentration *in vacuo* (Scheme 2).

**Scheme 2:**
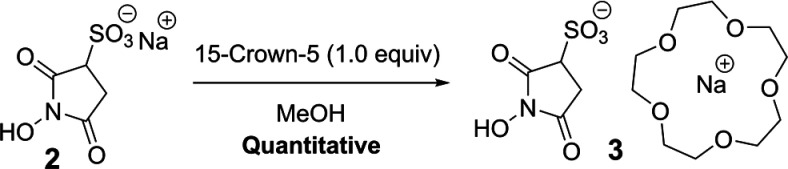
Synthesis of [Na(15-Crown-5)] Sulfo-NHS Salt **3** from Commercially Available Feedstocks **2** and 15-Crown-5

The solubility of the newly prepared “crowned” C-Sulfo-NHS reagent, **3**, in a variety of organic solvents commonly used for esterification reactions was then measured and compared to that of **2** (Table 1, Figure S1, see **SI** for Methods). **3** was far more soluble than **2** in the organic solvents tested yet remained highly soluble in water; a 65-fold increase in the DMF solubility was achieved for a mere 25% decrease in the water solubility. The combined enhancement in organic-solubility and preservation of the water solubility of compound **3** was encouraging, as it suggested that C-Sulfo-NHS esters could be prepared from **3** in organic solvents but remain highly water-soluble and thus still be used in the same applications as esters derived from **2**. The moderate decrease in water solubility was of little concern since bioconjugation reactions involving Sulfo-NHS esters typically only require concentrations within the μM → mM regime.^5,8^

**Table 1:** Solubility of Different Sulfo-NHS Salts in a Variety of Solvent Systems at 22 °C

Solvent	Solubility of **2**/mM	Solubility of **3**/mM
Water	2400	1800
DMSO	30	1300
DMF	4.6	300
Acetonitrile	<1	9.3
Dioxane	<1	1.1
THF	<1	<1

To demonstrate the utility of the organic-soluble C-Sulfo-NHS reagent, **3**, in the preparation of water-soluble activated esters from hydrophobic carboxylic acids, a series of activated esters (compounds **5**–**7**, see Figure 1) were prepared using carboxylic acid functionalized triazabutadiene **4** and reagents **1**, **2** and **3** via a typical NHS-type ester preparation method using DCC in DMF - a choice solvent for such esterification reactions. **4** was selected as the test carboxylic acid as while triazabutadienes have applications in bioconjugation, such as in the preparation of azo-dyes, triazabutadienes based on dimesityl scaffolds are poorly water-soluble.^14^ In addition to this, the chemical shifts of the protons of the benzoic acid motif of **4** are very sensitive to changes in the electronic nature of the carbonyl system, and thus could be used to conveniently report on the progress of the esterification reaction via ^1^H NMR.

**Figure 1:**
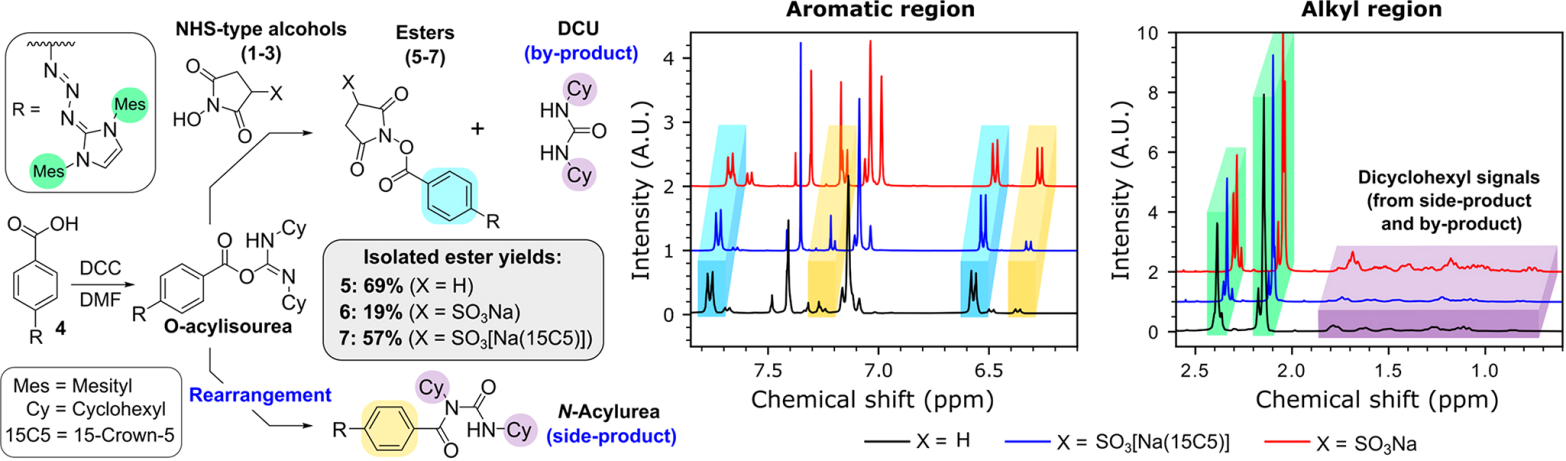
Left) The reaction pathway of the formation of NHS-type esters **5**–**7** via the reaction of **4** with DCC and NHS-type alcohols **1**–**3** (note: for **1**, X = H; for **2**, X = SO_3_Na; for **3**, X = SO_3_[Na(15C5)]), and the pathway leading to side-product formation. Right) ^1^H NMR analyses of the crude product mixtures (containing **5**, **6** or **7**) yielded from these reactions. The color coding indicates which ^1^H NMR signals correspond to which reaction products.

Reactions were performed overnight at rt, after which time the precipitated material was removed via filtration and the eluate was concentrated *in vacuo* (see SI). The crude product mixture isolated from the eluate was analyzed via ^1^H NMR to assess the distribution of products (Figure 1). The crude product mixture was then purified via flash silica column chromatography, and then the yields of the purified ester products **5**–**7** were calculated (Figure 1).

Carboxylic acids are activated by carbodiimides via the formation of an O-acylisourea, which serves as a far superior leaving group than −OH.^15^ However, if a nucleophile is not readily available to displace the O-acylisourea, an *N*-acylurea side-product forms as a result of the rearrangement of the O-acylisourea intermediate (Figure 1, left).^15^ As is evidenced by both the crude ^1^H NMR analyses and the yields tabulated in Figure 1, esterifications of **4** using **1** and **3** proceeded relatively cleanly, resulting in respectable yields of purified ester products **5** (an NHS ester) and **7** (a C-Sulfo-NHS ester) being obtained (69% and 57%, respectively). However, comparable esterification using **2** proceeded in poor yield, with only a 19% yield of **6** (a Sulfo-NHS ester) being recovered after purification. This correlates with a large quantity of the *N*-acylurea side-product being detected in the crude NMR of the reaction between **2** and **4** (isolated in a 38% yield). Isolation of the *N*-acylurea side-product was not achieved during purification of the other reaction mixtures, which can likely be attributed to **1** and **3** being completely dissolved in the reaction solution, thus making them more available for reaction with the O-acylisourea intermediate, whereas **2**, being poorly soluble in DMF, would be far less readily available.

Esters **5**–**7** were then used to bioconjugate to the mutant c-type cytochrome protein CjX183-D R51K,^16^ which has been engineered to present only two amines–its *N*-terminal amine and a single lysine residue. Equimolar quantities of the esters were delivered to identical solutions of CjX183-D R51K, and the resultant solutions were incubated in darkness for 1 h. The degree of amine labeling was evaluated by protein mass spectrometry, which allowed the relative performances of esters **5**–**7** to be assessed (see Figure 2). In order to definitively demonstrate that C-Sulfo-NHS esters are no less reactive than their pure “uncrowned” Sulfo-NHS ester counterparts when used in bioconjugation reactions, an additional experiment was also conducted in which one equivalent of 15-crown-5 was added to an aliquot of Sulfo-NHS ester **6** prior to delivery to the protein solution. The C-Sulfo-NHS ester used in this experiment, dubbed **7′**, can thus be assumed to be of identical purity to the sample of **6**, thus enabling a direct comparison in performance.

**Figure 2:**
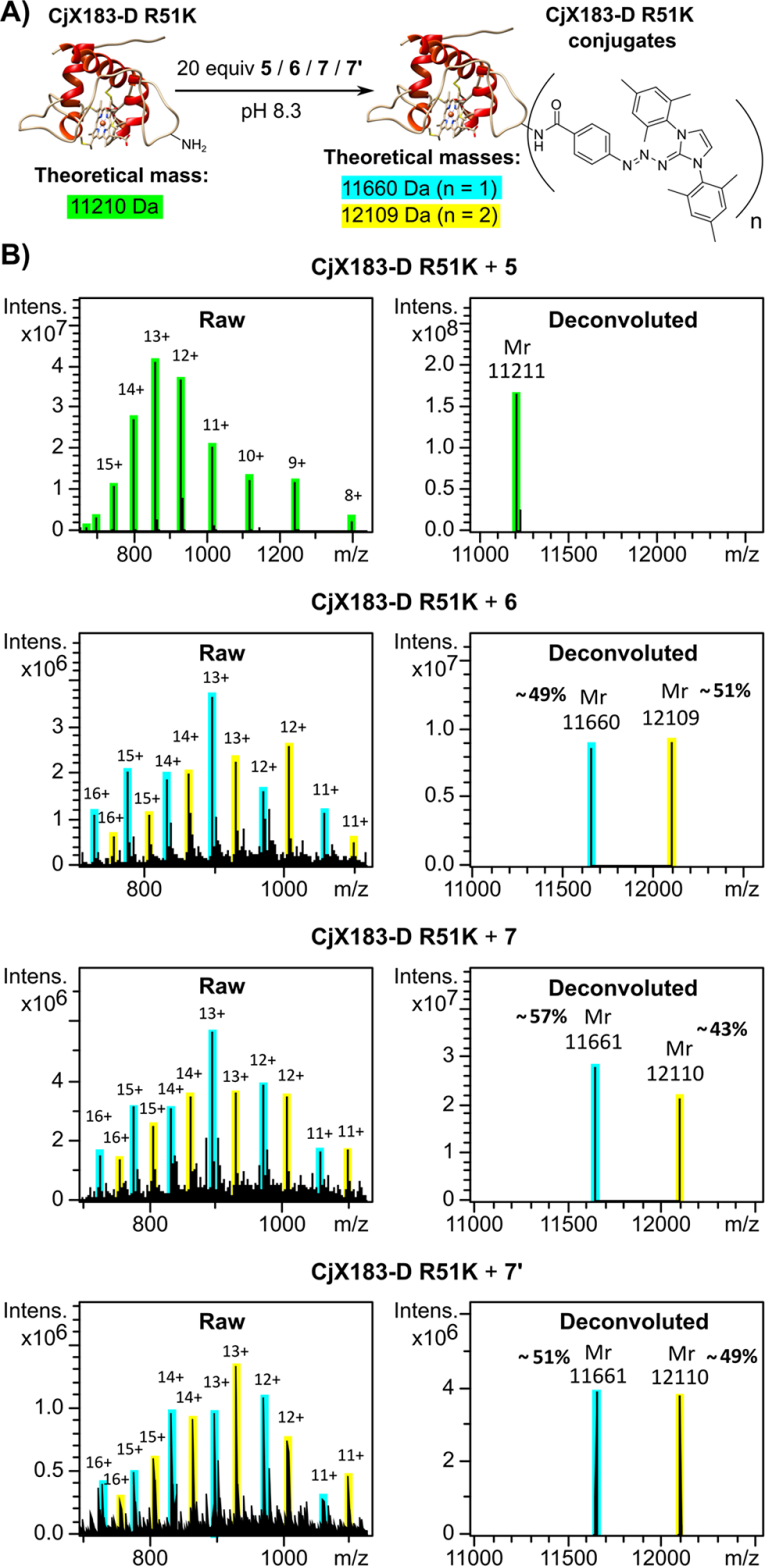
(A) Reaction scheme showing conjugation of the amine motifs of the protein CjX183-D R51K with activated esters **5**, **6**, **7** and **7′**. (B) Analysis of the resulting bioconjugation products via raw and deconvoluted protein mass spectrometry.

As is evident, **5** was completely ineffective in labeling protein amine residues, which can be attributed to its complete insolubility in aqueous solution. However, **6**, **7** and **7′**, which are all water-soluble, successfully labeled the protein amine residues of CjX183-D R51K with comparable efficiencies, with around 50% dual-labeling and 50% single-labeling being achieved. We rationalize the comparable performance of the “uncrowned” Sulfo-NHS ester sample **6** and the C-Sulfo-NHS ester samples **7** and **7′** by considering that, in buffered media containing many ionic species, any “crowned” sodium cations are unlikely to be associated with the sulfonate motif of a Sulfo-NHS ester at any given time, meaning the 15-crown-5 motif is statistically unlikely to confer additional steric hindrance upon the amide-bond formation reactions. (NB: cf 1 mM concentration of 15-crown-5 with 25 mM concentration of Na^+^).

We further sought to demonstrate that (1) C-Sulfo-NHS esters are no more hydrolytically unstable than their classic Sulfo-NHS ester counterparts and (2) no less selective toward amide bond formation. With regards to point (1), it is known that NHS-type alcohols absorb 268 nm radiation, whereas NHS-type ester motifs do not (Figure 3A).^5,17^ In order to exploit this convenient spectroscopic handle to assess the relative hydrolytic instabilities of C-Sulfo and Sulfo-NHS esters, we synthesized both the Sulfo-NHS and C-Sulfo esters of isobutyric acid (**8** and **9** respectively, Figure 3A) and monitored their rates of hydrolysis at a range of pHs in a range of different buffers via UV–vis spectroscopy (Figures S63–S73, see **SI** for Methods). Using this method, we showed the half-lives of Sulfo-NHS ester **8** in aqueous solutions to be akin to those of C-Sulfo-NHS ester **9** (Figure 3B, Figure S80). The decrease in the half-lives of NHS-type esters with increasing pH is already well documented,^5^ yet we noted with interest that the use of organic non-nucleophilic buffer salts (such as MES or BisTris) rather than inorganic buffer salts (such as phosphate) extended the hydrolytic half-lives of both **8** and **9** at both pH 6 and 7 (Figure 3B, Figure S80).

**Figure 3:**
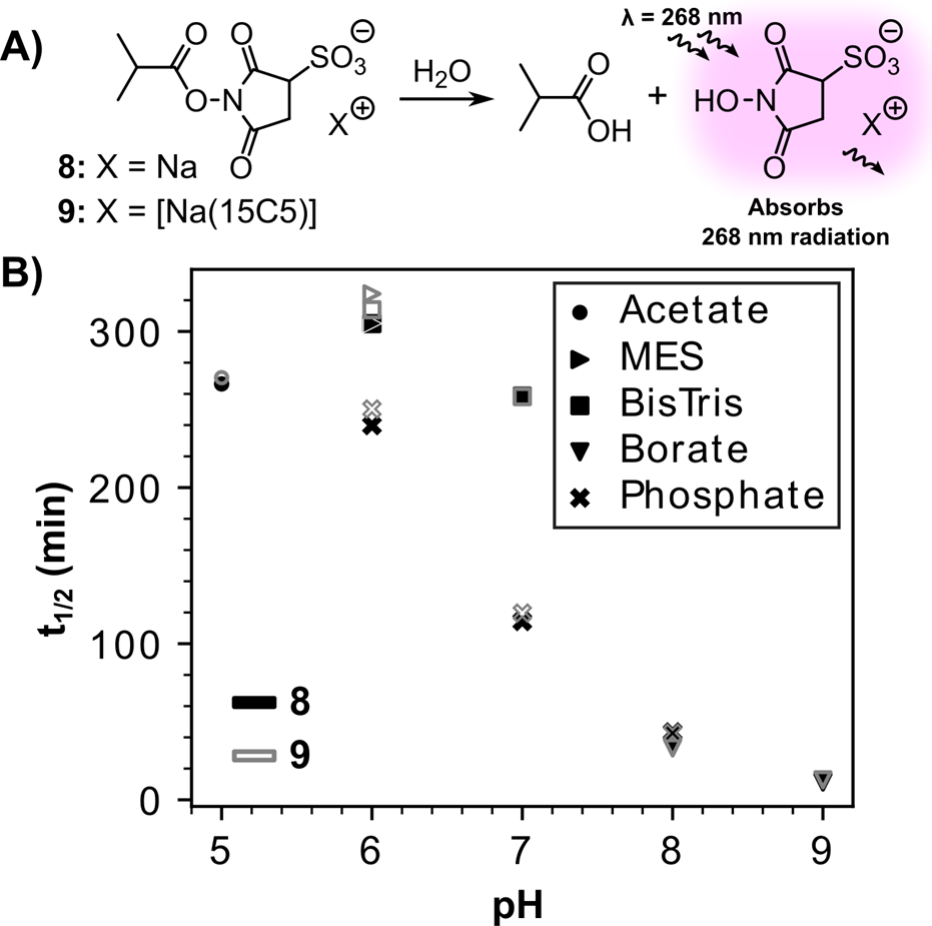
(A) The hydrolysis of Sulfo and C-Sulfo-NHS esters **8** and **9** can be observed by monitoring the UV–vis absorption at 268 nm. (B) The half-lives of esters **8** and **9** in a range of buffered aqueous solutions were at 22 °C.

While it is known that thiol groups can react with NHS-type esters during bioconjugation reactions to produce thioesters, surface-exposed thiol functionalities are relatively scarce in proteins. In addition to this, the propensity of thioesters to either hydrolyze or exchange with amine nucleophiles means that NHS-esters can be used to selectively generate amide-conjugated products, even in the presence of thiols.^5,18^ In order to address point (2) above and demonstrate that C-Sulfo-NHS esters also display selectivity for amine residues over thiol residues, competition experiments between *N*-acetyl lysine and *N*-acetyl cysteine for either **8** or **9** were conducted at both pH 8.3 and pH 7.5 (Figure S74). Subsequent analysis of these reaction solutions via both ^1^H NMR and LC-MS found the product distributions to be comparable when either **8** or **9** was used and showed the efficiency and selectivity for conjugation to lysine was greater at pH 8.3 than at pH 7.5 (Tables S2–S3, Figures S74–S79, see **SI** for Methods). Thus, the presence of 15-crown-5 has no effect on Lys versus Cys selectivity.

Having thus validated that **3** can be used to prepare water-soluble C-Sulfo-NHS esters from hydrophobic carboxylic acids (Figure 1) and that C-Sulfo-NHS esters have comparable reactivity, selectivity and hydrolytic stability to their “uncrowned” counterparts (Figure 3), we sought to apply the method in a typical application–the appending of an otherwise hydrophobic fluorophore to a protein that contains 10 lysine residues and an *N*-terminal amine. To this end, the fluorescent and hydrophobic carboxylic acid 1-pyrenebutyric acid, **10**, was selected (Figure 4). Pyrene is not only a popular fluorophore, its ability to pi-stack onto graphitic carbon surfaces means that this functional group is frequently appended to biomolecules and designer polymers in order to fabricate electrochemical sensors and devices.^19−23^ Notably, the Sulfo-NHS-ester of 1-pyrenebutyric acid is not commercially available.

**Figure 4:**
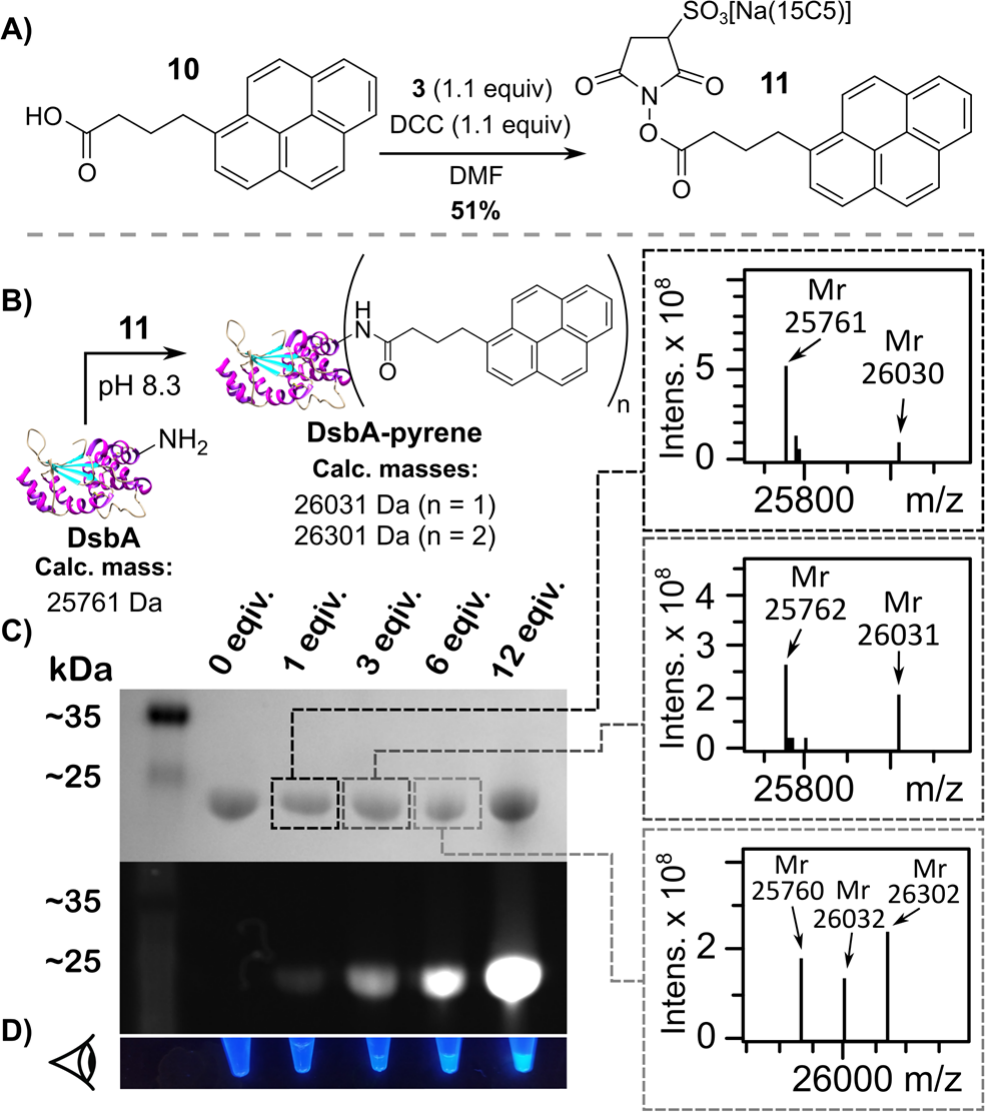
(A) The preparation of the C-Sulfo-NHS ester of 1-pyrenebutyric acid, **11**, was from **10** and **3**. (B) The preparation of fluorescent, pyrene-conjugated DsbA occurred via the reaction of the protein amine motifs with **11**. (C) Validation of the successful bioconjugation of DsbA to 1-pyrenebutyric acid motifs via SDS-PAGE gel analysis and protein mass spectrometry. (D) Photograph of the purified pyrene-labeled DsbA samples under irradiation with 365 nm UV-light.

The esterification reaction between **3** and **10** proceeded in a respectable yield, and the resultant water-soluble C-Sulfo-NHS ester **11** was used to label the amine residues of a mutant DsbA protein (a bacterial thiol disulfide oxidoreductase). Successful bioconjugation was readily verified via SDS-PAGE gel and mass spectrometry analyses (Figure 4, Figure S82).

A range of labeled products were observed via mass spectrometry, as would be expected when forming amide bonds to a protein which contains 10 lysine residues (Figure 4C). When 1 or 3 equiv of **11** were delivered, only unlabeled and singly labeled protein species were discernible via mass spectrometry, whereas when using 6 equiv of **11** a distribution of unlabeled, singly labeled and dual labeled protein products were observed. Visual inspection of the fluorescent bands of the SDS-PAGE gel supports the MS data, clearly demonstrating that the intensity of fluorescence, and thus the degree of labeling, increases with the number of equivalents of **11** delivered to the protein sample (Figure 4).

When 12 equiv of **11** were used to react with DsbA, the mass spectrum obtained was of too great a complexity to permit accurate deconvolution; this would be consistent with the presence of an even broader distribution in the range of labeled products. It is also clear from the intensity of the fluorescence (Figure 4) that an increased degree of protein labeling has been achieved when a greater number of equivalents of **11** have been used. This demonstrates that, even when attempting multivalent labeling of a single protein possessing multiple amine residues, the water solubility of C-Sulfo-NHS esters is unlikely to be a limiting factor. This is an important consideration, as it is well understood that creating an optimal conjugation product using NHS-type esters requires the careful optimizing of reaction conditions, including the adjustment of both the molar ratios and concentrations of the NHS-type ester and the target molecule.^5^

In conclusion, we present a simple, low-cost, and scalable method that allows water-soluble Sulfo-NHS-type esters to be produced conveniently in high yields from water-insoluble carboxylic acids. The critical reagent, C-Sulfo-NHS (**3**) can be accessed by simply treating commercially available Sulfo-NHS (**2**) with 15-crown-5. After synthesis in organic solvents, C-Sulfo-NHS esters derived from **3** remain soluble in aqueous media and undergo classical amide-bond formation when reacted with protein amine residues, demonstrating reaction efficiencies, hydrolytic stabilities, and selectivities comparable to those of their classic Sulfo-NHS ester counterparts.

It has long been appreciated that there is a degree of nuance surrounding the selection of the most appropriate carbodiimide reagent (DCC, DIC or EDC) for a given esterification or amide-bond formation reaction, with experimentalists choosing their reagent by considering its solubility and the ease of product purification.^4^ We suggest that the same consideration should be applied when selecting the NHS-type reagent to be used in the preparation of an activated ester, with experimentalists considering the properties of **1**, **2** and **3** before selecting the most appropriate reagent for their system. While the creation of any bioconjugate product using NHS-type esters requires the careful optimization of reaction conditions, we hope that the use of **3** could make accessing certain NHS-type esters less arduous.

## Supporting Information Available

Experimental details; UV–vis, NMR, FI-IR, and mass spectrometry data (PDF)

## ABBREVIATIONS

NHS: *N*-hydroxysuccinimide
Sulfo-NHS: *N*-hydroxysulfosuccinimide sodium salt
15C5: 15-crown-5
C-Sulfo-NHS: *N*-hydroxysulfosuccinimide [Na(15-crown-5)] salt
