# Supplementary material for: Crossing the Solubility Rubicon: 15-Crown-5 Facilitates the Preparation of Water-Soluble Sulfo-NHS Esters in Organic Solvents
Source: Bioconjug Chem. 2023 Dec 12;35(1):22–7. doi: 10.1021/acs.bioconjchem.3c00396 (PMC10797585; doi:10.1021/acs.bioconjchem.3c00396)
Supplement: Supplementary file 1 — bc3c00396_si_001.pdf [file bc3c00396_si_001.pdf]

Supporting Information

Crossing the Solubility Rubicon: 15-Crown-5 Facilitates the Preparation of Water-Soluble Sulfo-NHS Esters in Organic Solvents

Nicholas D. J. Yates,[a,*] Connor G. Miles,[a] Christopher D. Spicer,[a] Martin A. Fascione, [a] Alison Parkin[a,*]

[a] Department of Chemistry, University of York, York, North Yorkshire, YO10 5DD, United Kingdom

* Email: nicholas.yates@york.ac.uk

* Email: alison.parkin@york.ac.uk

Contents

General considerations **4**

Solubility measurements **5**

Synthesis and characterisation of molecules **6**

N-Hydroxysulfosuccinimide [Na(15-crown-5)] salt **3** 6

Methyl 4-azidobenzoate **S1** 12

Methyl (E)-4-((1,3-dimesityl-1,3-dihydro-2H-imidazol-2-ylidene)triaz-1-en-1-yl)benzoate **S2** 14

(E)-4-((1,3-dimesityl-1,3-dihydro-2H-imidazol-2-ylidene)triaz-1-en-1-yl)benzoic acid **4** 19

General method of triazabutadiene NHS ester preparation **24**

2,5-dioxopyrrolidin-1-yl (E)-4-((1,3-dimesityl-1,3-dihydro-2H-imidazol-2-ylidene)triaz-1-en-1-yl)benzoate **5** 25

(E)-1-((4-((1,3-dimesityl-1,3-dihydro-2H-imidazol-2-ylidene)triaz-1-en-1-yl)benzoyl)oxy)-2,5-dioxopyrrolidine-3-sulfonate sodium salt **6** 30

(E)-1-((4-((1,3-dimesityl-1,3-dihydro-2H-imidazol-2-ylidene)triaz-1-en-1-yl)benzoyl)oxy)-2,5-dioxopyrrolidine-3-sulfonate sodium salt 15-crown-5 complex **7** 35

Derivatives of isobutyric acid **40**

Sodium 1-(isobutyryloxy)-2,5-dioxopyrrolidine-3-sulfonate **8** 40

Sodium 1-(isobutyryloxy)-2,5-dioxopyrrolidine-3-sulfonate 15-crown-5 complex **9** 45

*N*α-Acetyl-*Nε*-isobutyryl-L-lysine **S3** 51

(R)-2-acetamido-3-(isobutyrylthio)propanoic acid **S4** 56

2,5-dioxo-1-((4-(pyren-1-yl)butanoyl)oxy)pyrrolidine-3-sulfonate sodium salt 15-crown-5 complex **11** **61**

Protein preparation, molecular weight calculations and SDS page gel analysis **67**

Protein preparation and concentration quantification 67

Analysis of proteins via Sodium dodecyl sulfate polyacrylamide gel electrophoresis (SDS-PAGE) 67

Calculation of protein theoretical masses: 68

Bioconjugation of proteins **69**

Reaction of CjX183-D R51K with esters **5-7/7'** 69

Reaction of DsbA with **11** 69

Monitoring the rate of Sulfo-NHS-type ester hydrolysis via UV-vis spectroscopy **70**

The hydrolysis of Sulfo-NHS-type esters 70

Derivation of rate equations 70

Experimental setup data processing 73

Experimental data 75

Determining the selectivity of Sulfo-NHS-type esters 84

Competition experiment setup 84

Analysis via LC-MS 84

Analysis via ^{1}H-NMR 87

Additional Figures 89

Relative hydrolysis rates and half-lives of Sulfo and C-Sulfo-NHS esters **8** and **9** 89

Protein mass spectrometry of CjX183-D R51K bioconjugates 90

Protein mass spectrometry of DsbA bioconjugates 91

Crude ^{1}H-NMR spectra of esters derived from **4** 92

^{1}H-NMR and (ESI-HRMS) spectra of the urea side-product derived from **4** 95

^{1}H-NMR spectra from selectivity study of **8** and **9** 97

LC-MS traces of standard solutions of **S3** and **S4** 101

References 102

General considerations

Reagents were purchased from Sigma-Aldrich and used as supplied. Anhydrous solvents were dried over a PureSolv MD 7 Solvent Purification System. Anhydrous solvents were used in reactions unless otherwise stated. GPR-grade solvents were used for flash chromatography purposes. Solution-phase synthetic reactions were carried out using oven-dried glassware. All concentrations were performed *in vacuo* unless otherwise stated. Thin layer chromatography was carried out on Merck silica gel 60 F254 precoated aluminium foil sheets and these were visualized using UV light. Unless otherwise indicated, flash column chromatography was performed on Supelco® silica gel (particle size 35–75 µm, pore diameter 60 Å, 220-440 mesh) and the solvent system used is recorded in parentheses.

Proton and carbon nuclear magnetic resonance (^{1}H and ^{13}C NMR respectively) spectra were recorded on a Jeol ECX-400 (400 MHz) spectrometer. All chemical shifts are quoted on the δ scale in ppm using residual solvent as the internal standard. Coupling constants (J) are reported in Hz with the following splitting abbreviations: s = singlet, d = doublet, t = triplet, q = quartet, m = multiplet, app = apparent, br = broad. $m^{AA'BB}$ refers to the multiplet pattern observed for 1,4-disubsituted aryl systems.

Small-molecule high resolution mass spectrometry (HRMS) data were obtained at RT on a Bruker Daltonics microTOF mass spectrometer coupled to an Agilent 1200 series LC system at The University York Centre of Excellence in Mass Spectrometry (CoEMS). Nominal and exact m/z values are reported in Daltons. High Performance Liquid Chromatography-Electrospray Ionization Mass Spectrometry (LC-MS) of protein samples was performed using a Dionex UltiMate® 3000 Ci Rapid Separation LC system equipped with an UltiMate® 3000 photodiode array detector probing at 250–400 nm, coupled to a HCT ultra ETD II (Bruker Daltonics) ion trap spectrometer, using Chromeleon® 6.80 SR12 software (ThermoScientific), esquireControl version 6.2, Build 62.24 software (Bruker Daltonics), and Bruker compass HyStar 3.2-SR2, HyStar version 3.2, Build 44 software (Bruker Daltonics) at CoEMS. Protein samples were analysed without the use of a column at RT. Mass spectrometry data analysis was performed using ESI Compass 1.3 DataAnalysis, version 4.4 software (Bruker Daltonics). All mass spectrometry was conducted in positive ion mode unless stated otherwise, and the esquireControl “Compound Stability” setting was set at “100%” unless stated otherwise.

Solubility measurements

The solubility of compounds **2** and **3** in a variety of solvents were measured at 22 °C by first preparing saturated solutions of these compounds in the solvents of interest (i.e. adding compound **2** or **3** to the solvent with stirring until a substantial amount of undissolved material remained). The resultant solutions were then sonicated for 10 minutes, and undissolved material was then removed via filtration, passing the saturated solution into a pre-weighed vial. The eluates were then weighed (mass of solvent + dissolved solid = A). All solvent was then either removed *in vacuo*, or in the case of DMSO solutions, diluted to 5% v/v with dioxane, flash-frozen and lyophilized. The mass of compound **2** or **3** recovered was recorded (B). The molar quantity of recovered compound **2** or **3** was then calculated. By subtracting mass B from mass A, the mass of solvent in the saturated solution could be calculated. Using the densities of the solvents, it was then possible to calculate the volume of solvent removed and thus the solubility of compounds **2** and **3** in the solvents of interest.

A plot of the solubility of NHS-type alcohols **2** and **3** in a variety of solvents, ranked by their Snyder polarity index, can be found below.

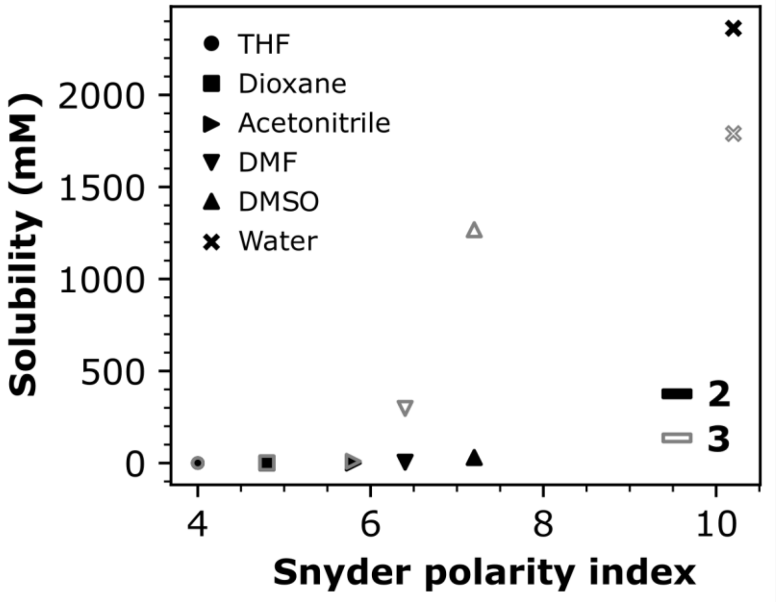

Figure S 1. The solubility of NHS-type alcohols **2** and **3** in a variety of solvents, ranked by their Snyder polarity index.[1]

Synthesis and characterisation of molecules

N-Hydroxysulfosuccinimide [Na(15-crown-5)] salt 3

To commercially available *N*-hydroxysulfosuccinimide sodium salt **2** (431 mg, 1.98 mmol) was added 15-crown-5 (0.437 g, 0.198 mmol). The resultant solution was then stirred, with heating at 50 °C until no more solid became dissolved (approx. 20 mins). The resultant mixture was then filtered, and the filtrate concentrated *in vacuo* to yield *N*-hydroxysulfosuccinimide [Na(15-crown-5)] salt **3** in quantitative yield.

^{1}H-NMR (400 MHz, DMF-d_7) δ_H 11.24 (br s, 1H), 3.86 (m, 1H), 3.00 (dd, *J* = 18.00, 8.50 Hz, 1H), 2.90 (dd, *J* = 18.00, 2.30 Hz, 1H).

^{13}C-NMR (101 MHz, DMF-d_7): δ_c 172.5, 169.8, 70.2, 57.8, 32.0.

FT-IR (ATR) (υmax/cm^{-1}): 2927 (O-H stretch), 1706 (C=O stretch), 1087 (C-O stretch, ether).

(ESI)HRMS: (Negative mode) Found 193.9764, $C_4H_4NO_6S^-$ requires 193.9765. (Positive mode) Found 243.1201, $C_{10}H_{20}NaO_5^+$ requires 243.1203.

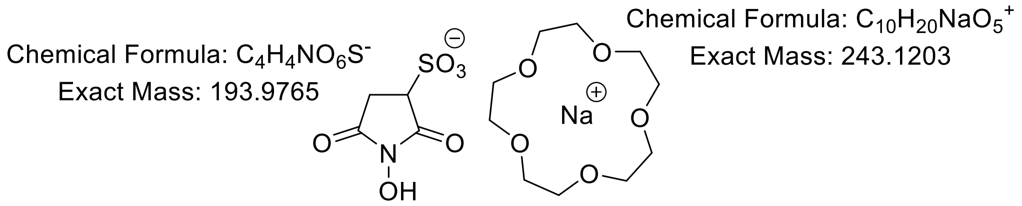

Figure S 2. The structure of *N*-hydroxysulfosuccinimide [Na(15-crown-5)] salt **3**.

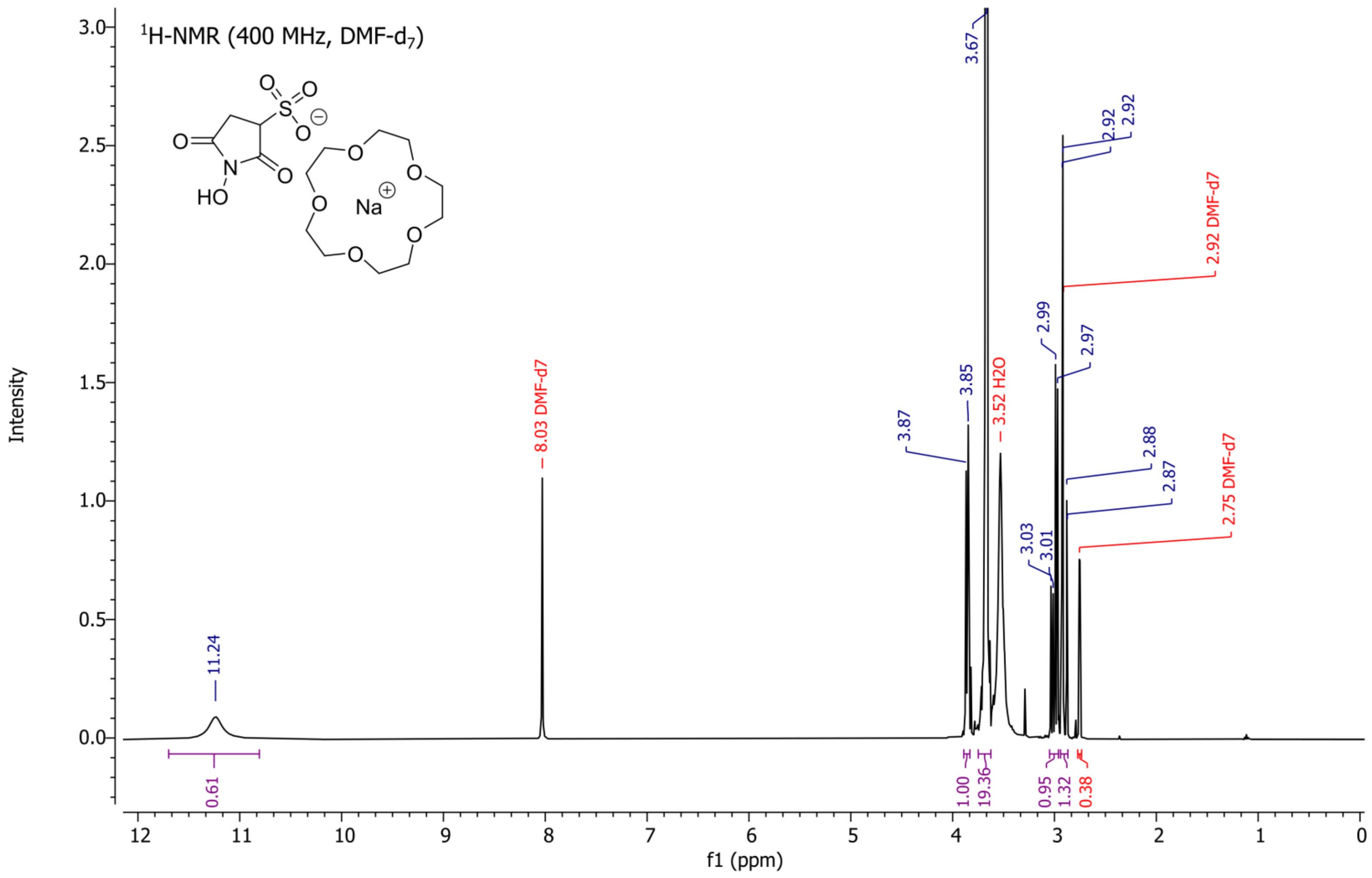

Figure S 3. ^{1}H-NMR spectrum of *N*-hydroxysulfosuccinimide [Na(15-crown-5)] salt **3**.

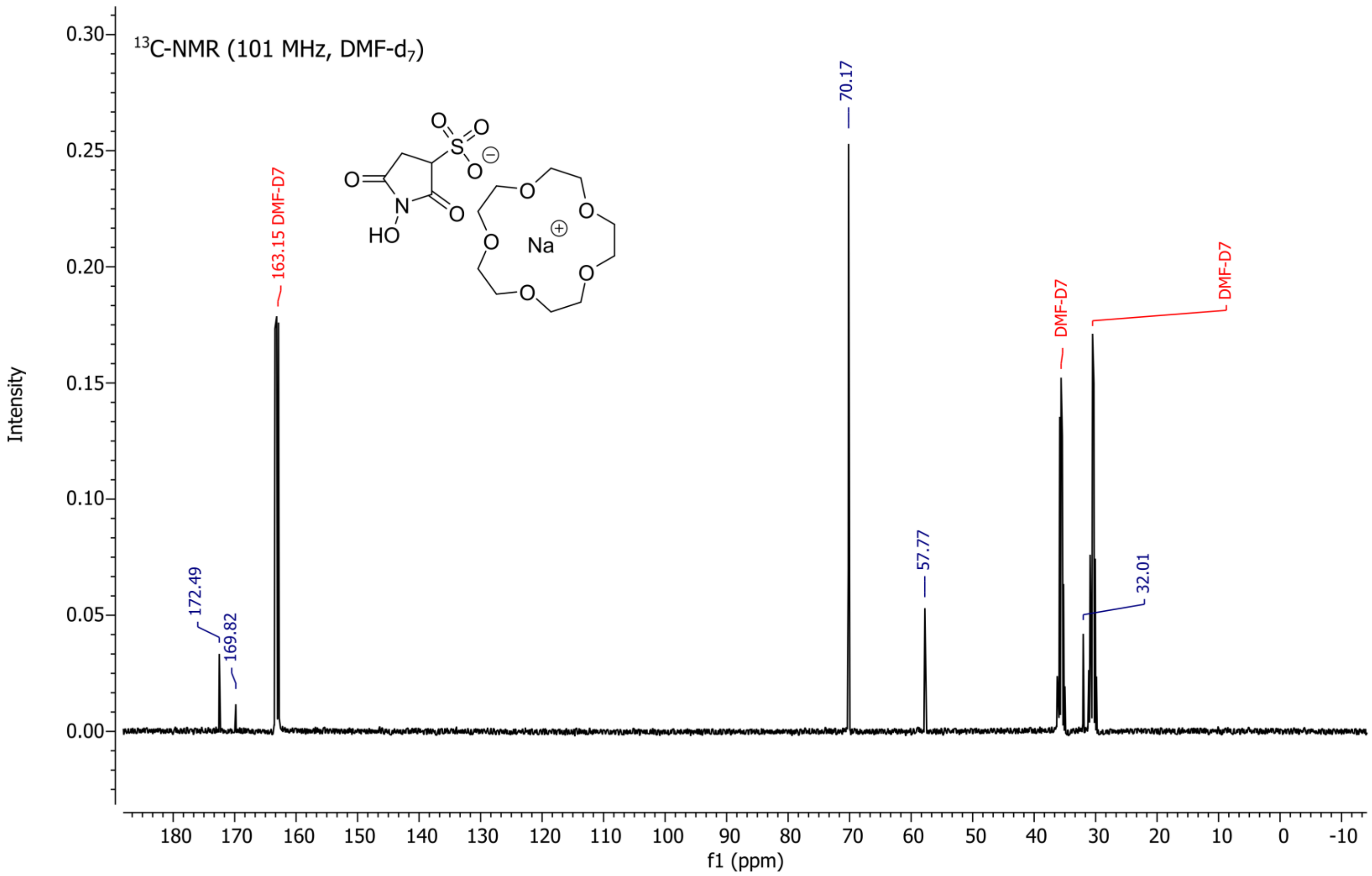

Figure S 4. ^{13}C-NMR spectrum of *N*-hydroxysulfosuccinimide [Na(15-crown-5)] salt **3**.

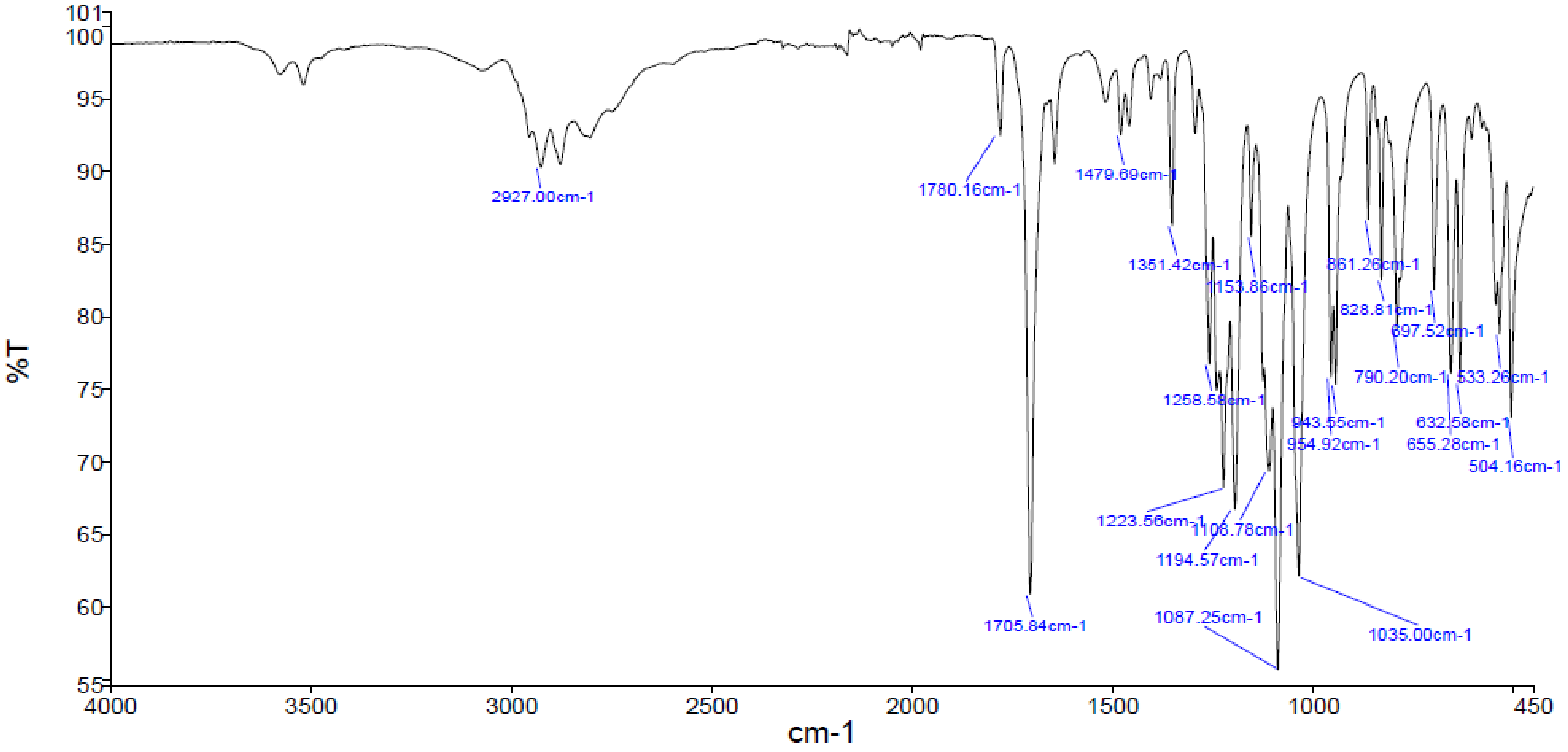

Figure S 5. FT-IR (ATR) spectrum of *N*-hydroxysulfosuccinimide [Na(15-crown-5)] salt **3**.

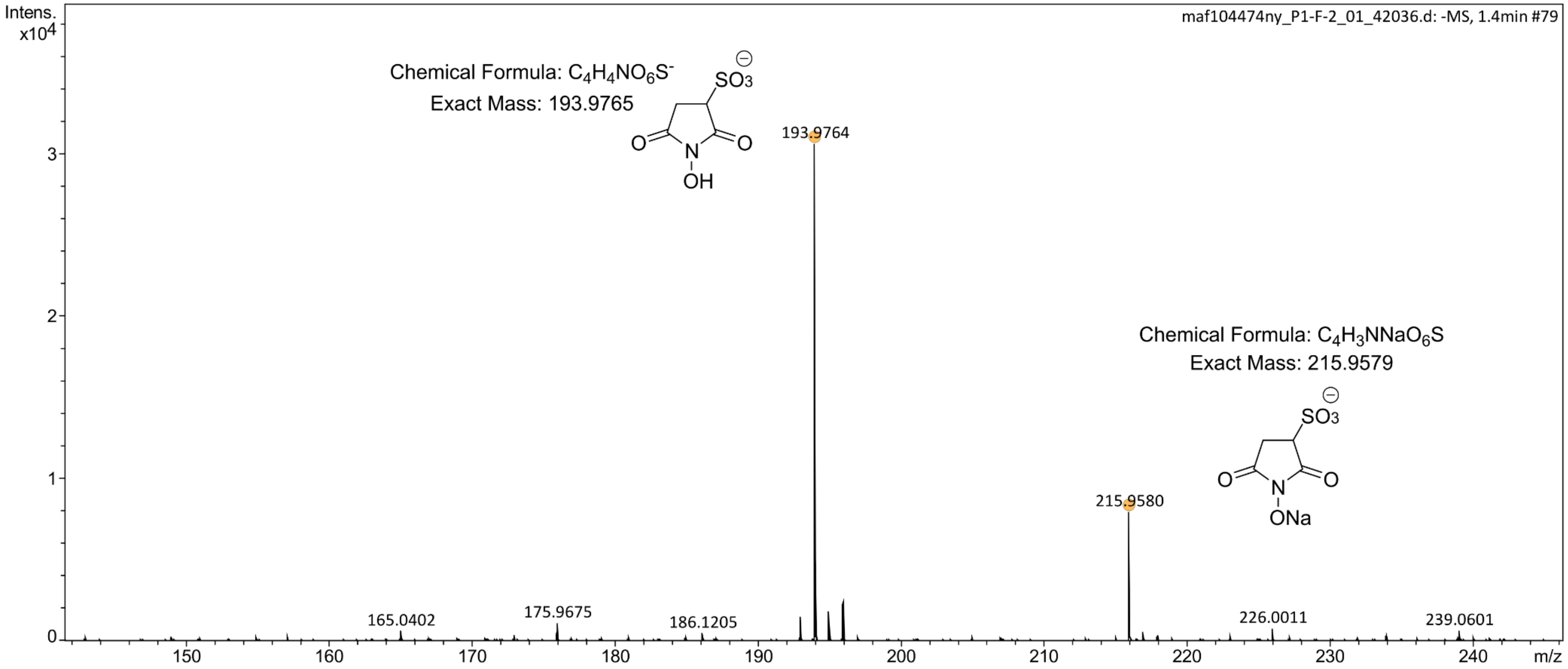

Meas. m/z	#	Ion Formula	m/z	err [ppm]	err [mDa]	mSigma	Mean err [ppm]
193.9764	1	C4H4NO6S	193.9765	0.2	0.0	12.6	-0.8
215.9580	1	C4H3NNaO6S	215.9584	2.0	0.4	10.4	-0.5

Figure S 6. Negative-mode (ESI)HRMS of *N*-hydroxysulfosuccinimide [Na(15-crown-5)] salt **3**.

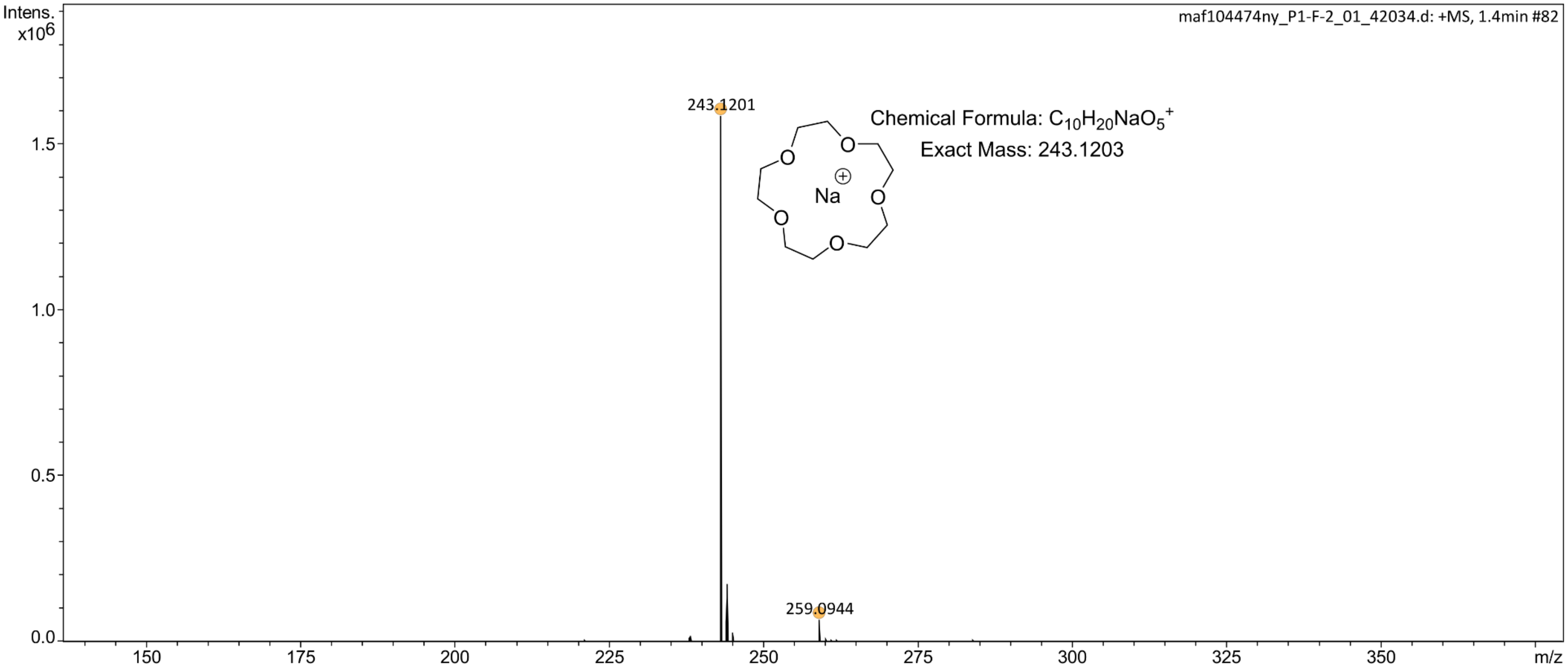

Meas. m/z	#	Ion Formula	m/z	err [ppm]	err [mDa]	mSigma	Mean err [ppm]
243.1201	1	C10H20NaO5	243.1203	0.6	0.2	1.0	1.0
259.0944	1	C10H20KO5	259.0942	-0.5	-0.1	8.5	0.7

Figure S 7. Positive-mode (ESI)HRMS of *N*-hydroxysulfosuccinimide [Na(15-crown-5)] salt **3**.

Methyl 4-azidobenzoate S1

To a solution of methyl 4-aminobenzoate (1.06 g, 7.00 mmol) in water (40 mL) was added 4.2 M hydrochloric acid (12 mL). The resultant solution was then cooled to 0 °C. A solution of $NaNO_2$ (0.531 g, 7.70 mmol, 1.1 equiv) in water (10 mL) and then added dropwise to the reaction mixture, which was then stirred for 20 minutes at 0 °C. Diethyl ether (20 mL) was then added, forming a biphasic system. NaN_3 (0.546 g, 8.4 mmol, 1.2 equiv) dissolved in the minimum volume of water was added, whereupon gas was emitted from the aqueous layer. The reaction mixture was stirred for 1 hour at room temperature, whereafter the resultant mixture was transferred to a separating funnel. The diethyl ether layer was collected, and the aqueous layer was further washed with diethyl ether (2 x 40 mL). All organic extractions were then combined and were washed with sodium bicarbonate (100 mL), water (100 mL) and brine (100 mL). The organic layer was then dried over $MgSO_4$ and concentrated *in vacuo* to yield **S1** as a yellow powder (1.27 g, 95%). ^{1}H-NMR was in agreement with that of the literature.[2]

^{1}H-NMR (400 MHz, $CDCl_3$): δ_H 8.04 (m, 2H), 7.08 (m, 2H), 3.92 (s, 1H).

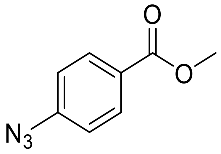

Figure S 8. The structure of methyl 4-azidobenzoate **S1**.

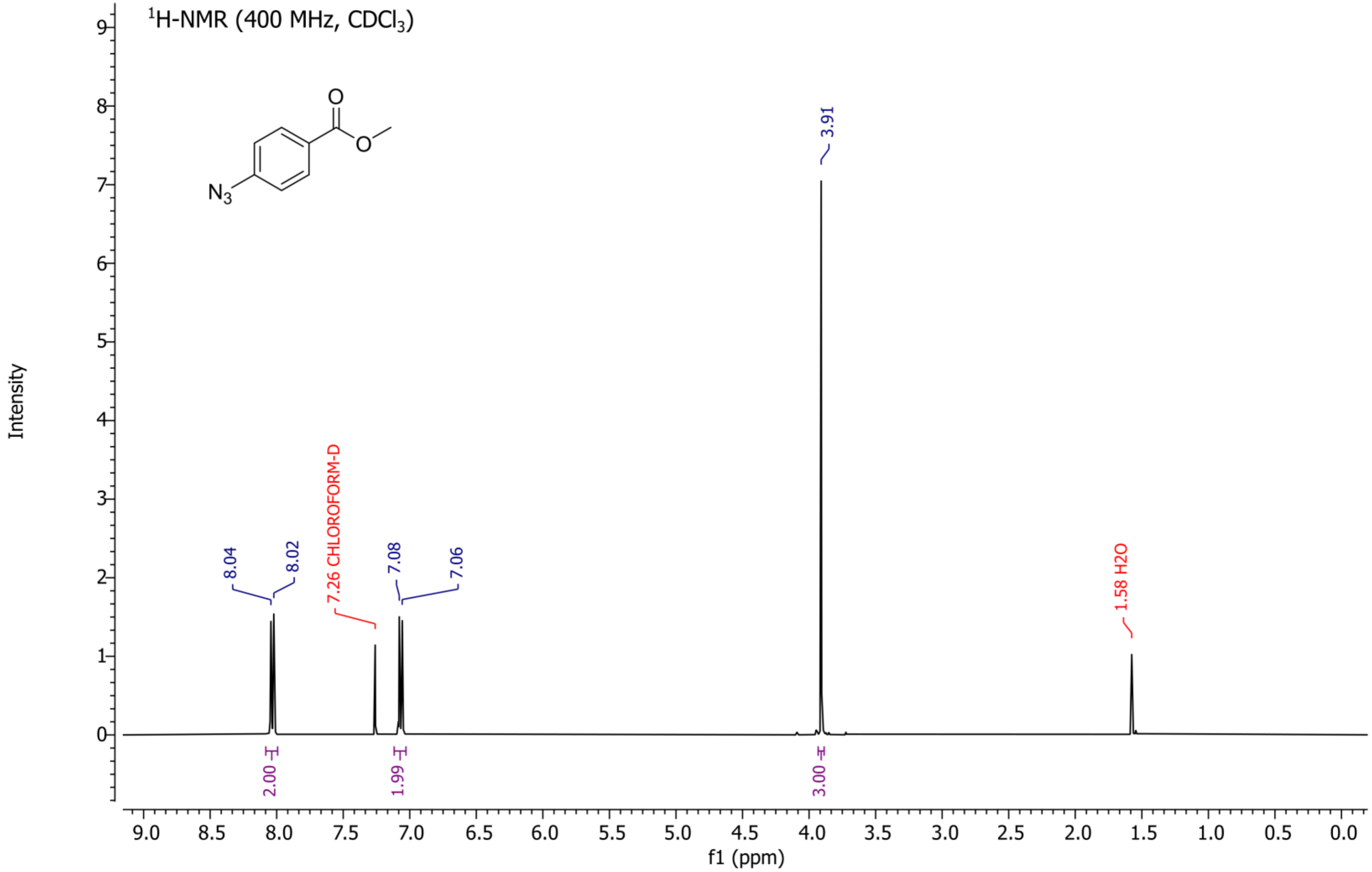

Figure S 9. ^{1}H-NMR spectrum of **S1**.

Methyl (E)-4-((1,3-dimesityl-1,3-dihydro-2H-imidazol-2-ylidene)triaz-1-en-1-yl)benzoate S2

To a solution of **S1** (68 mg, 0.383 mmol) and 1,3-bis(2,4,6-trimethylphenyl)imidazolium chloride (130 mg, 0.383 mmol) dissolved in the minimum volume of anhydrous DMSO was added potassium *tert*-butoxide (43 mg, 0.383 mmol). The resultant solution was stirred overnight in the dark under N_2, after which time DCM (40 mL) was added. The resultant solution was then transferred to a separating funnel, and DMSO was removed via washing with water (3 × 40 mL), while retaining the organic layer. The organic layer was then dried over $MgSO_4$ and concentrated *in vacuo*. The crude residue was then purified via flash column chromatography (hexane → ethyl acetate to yield **S2** as a yellow/orange foamy solid (72 mg, 39%). Characterisations were in agreement with those in the literature.[3]

^{1}H-NMR (400 MHz, $CDCl_3$): δ_H 7.69 ($m^{AA'BB'}$, 2H), 7.00 (s, 4H), 6.63 (s, 2H), 6.57 ($m^{AA'BB'}$, 2H), 3.85 (s, 3H), 2.37 (s, 6H), 2.15 (s, 12H).

^{13}C-NMR (101 MHz, $CDCl_3$): δ_C 167.4, 155.4, 151.7, 139.1, 135.0, 134.0, 129.9, 129.5, 126.4, 121.0, 117.4, 51.9, 21.2, 18.1.

FT-IR (ATR) (υmax/cm^{-1}): 3058 (C-H stretch, aromatic), 2858 (C-H stretch, alkyl), 1705 (C=O stretching), 1525 (C-C stretch, aromatic), 1358 (C-N stretching), 1176 (C-N stretching), 853 (C-H bend, aromatic), 773 (C-H bend, aromatic).

(ESI)HRMS: Found $[M+H]^+$ 482.2560, $C_{29}H_{32}N_5O_2$ requires 482.2551.

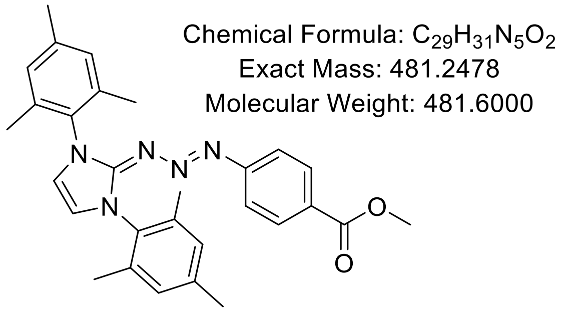

Figure S 10. The structure of methyl (E)-4-((1,3-dimesityl-1,3-dihydro-2H-imidazol-2-ylidene)triaz-1-en-1-yl)benzoate **S2**.

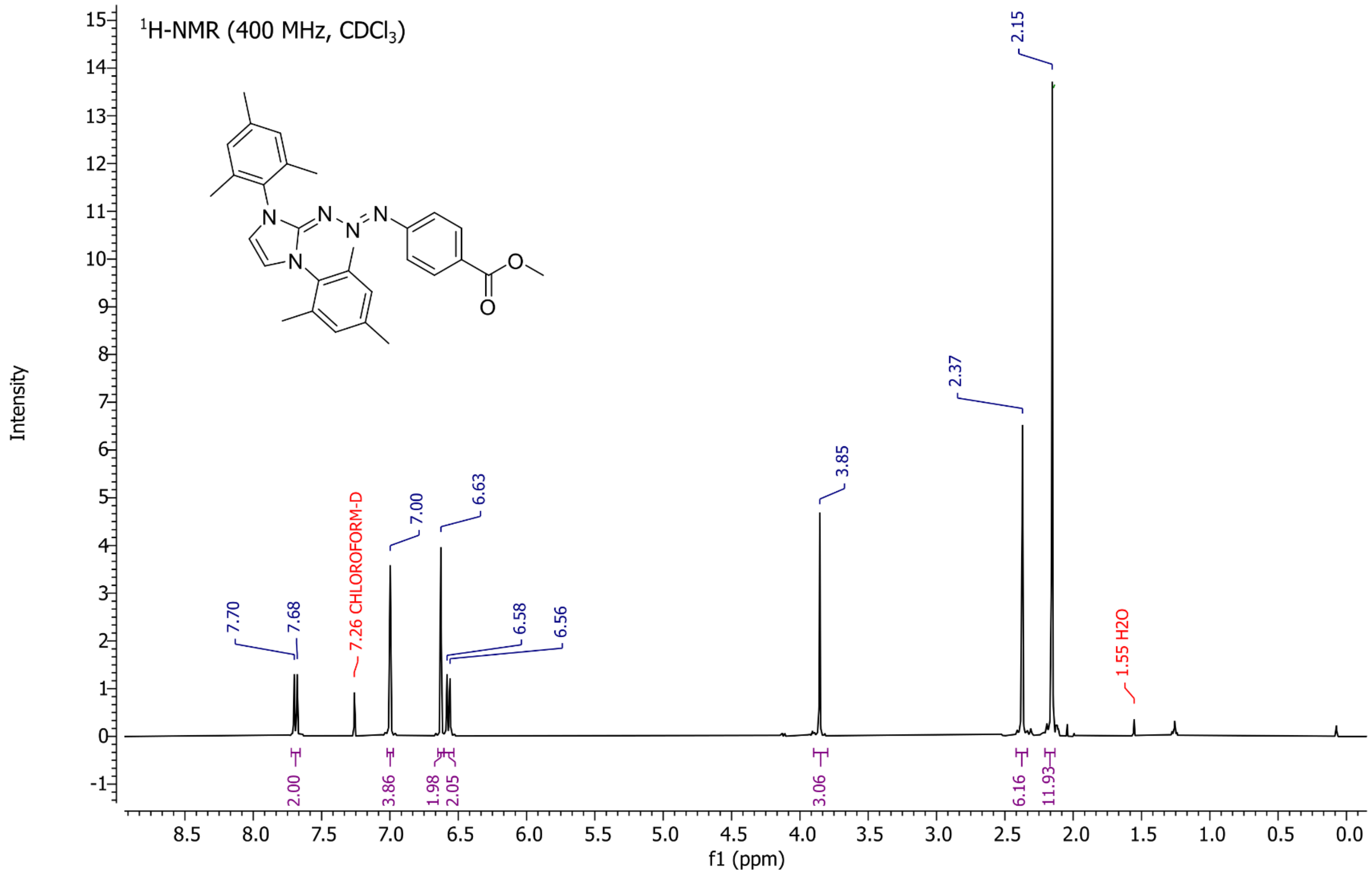

Figure S 11. ^{1}H-NMR spectrum of **S2**.

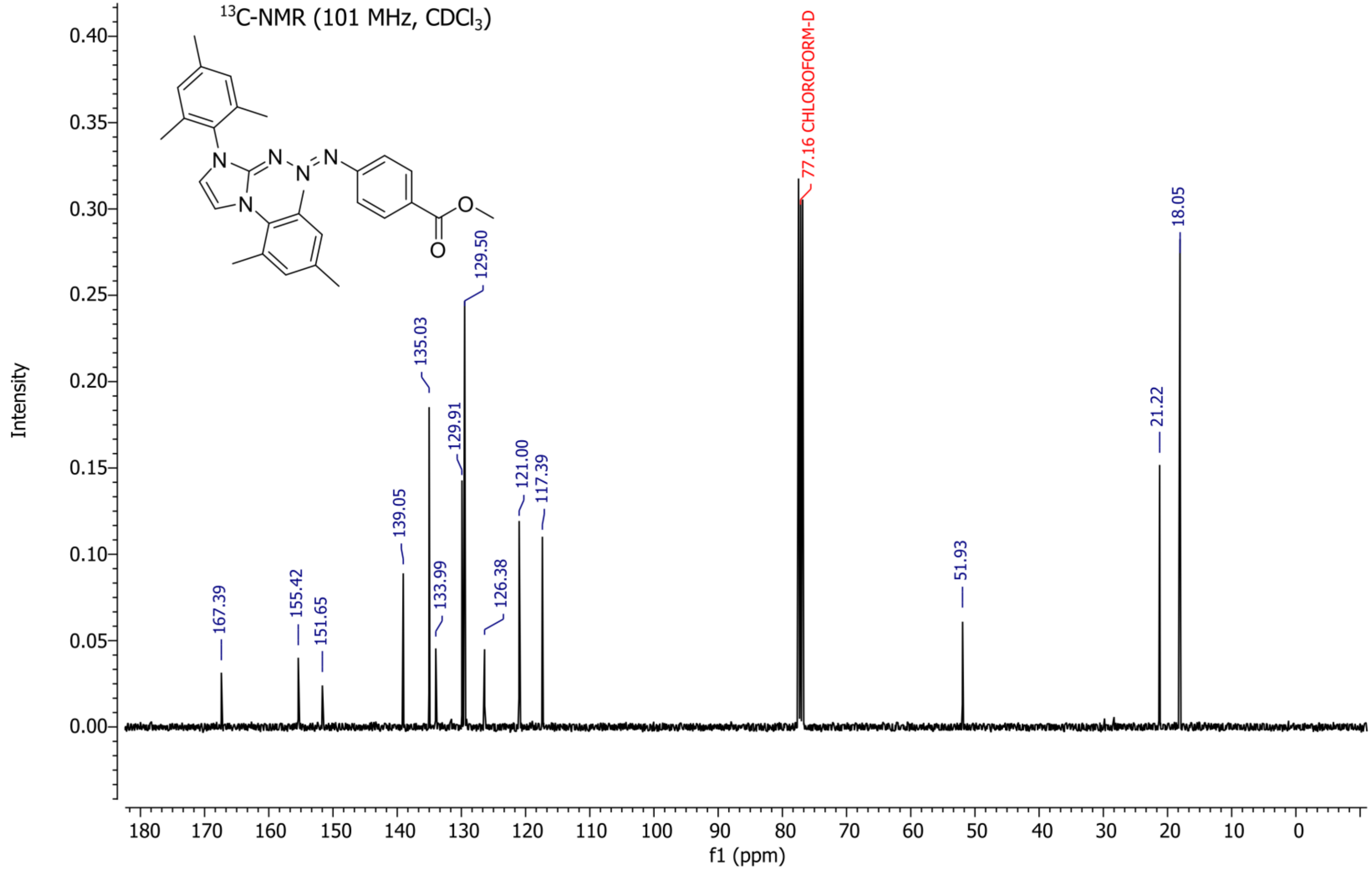

Figure S 12. ^{13}C-NMR spectrum of **S2**.

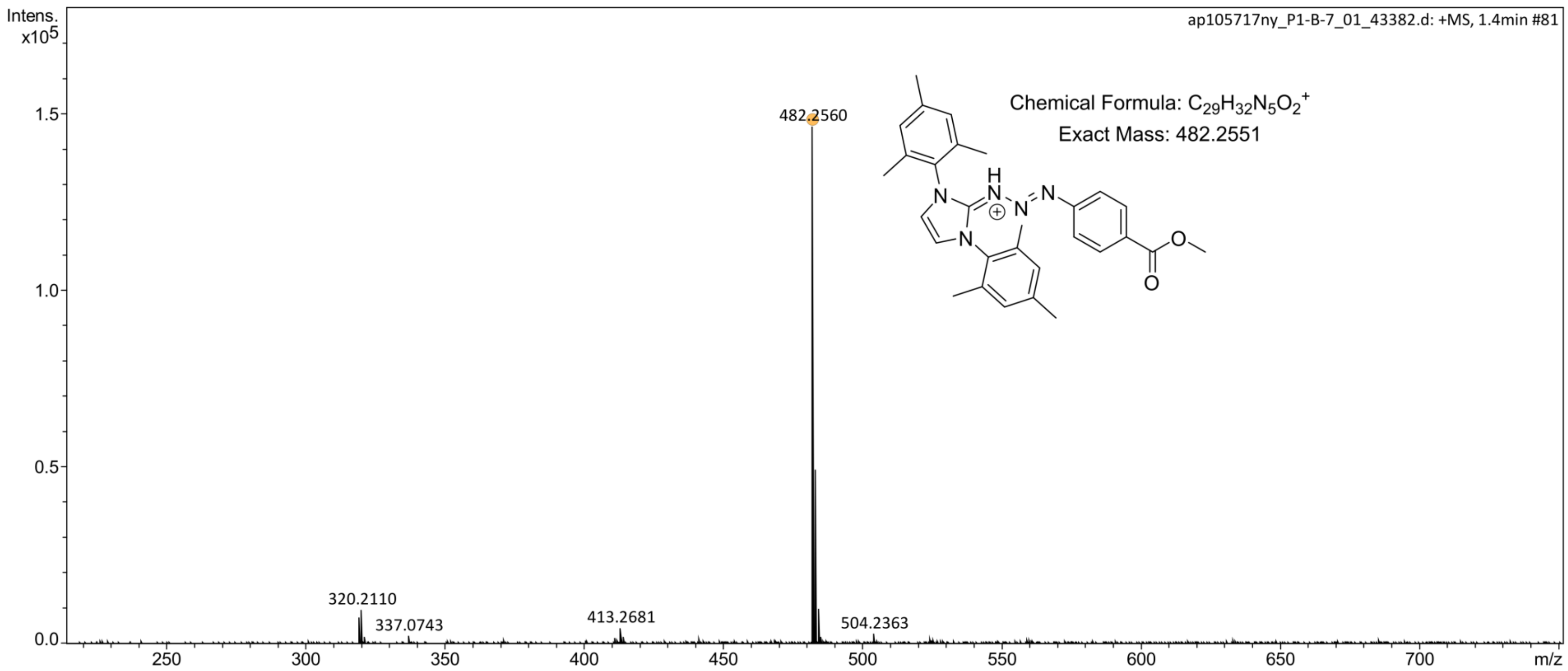

Meas. m/z	#	Ion Formula	m/z	err [ppm]	err [mDa]	mSigma	Mean err [ppm]
482.2560	1	C29H32N5O2	482.2551	-2.1	-1.0	4.0	-3.5

Figure S 13. (ESI)HRMS of **S2**.

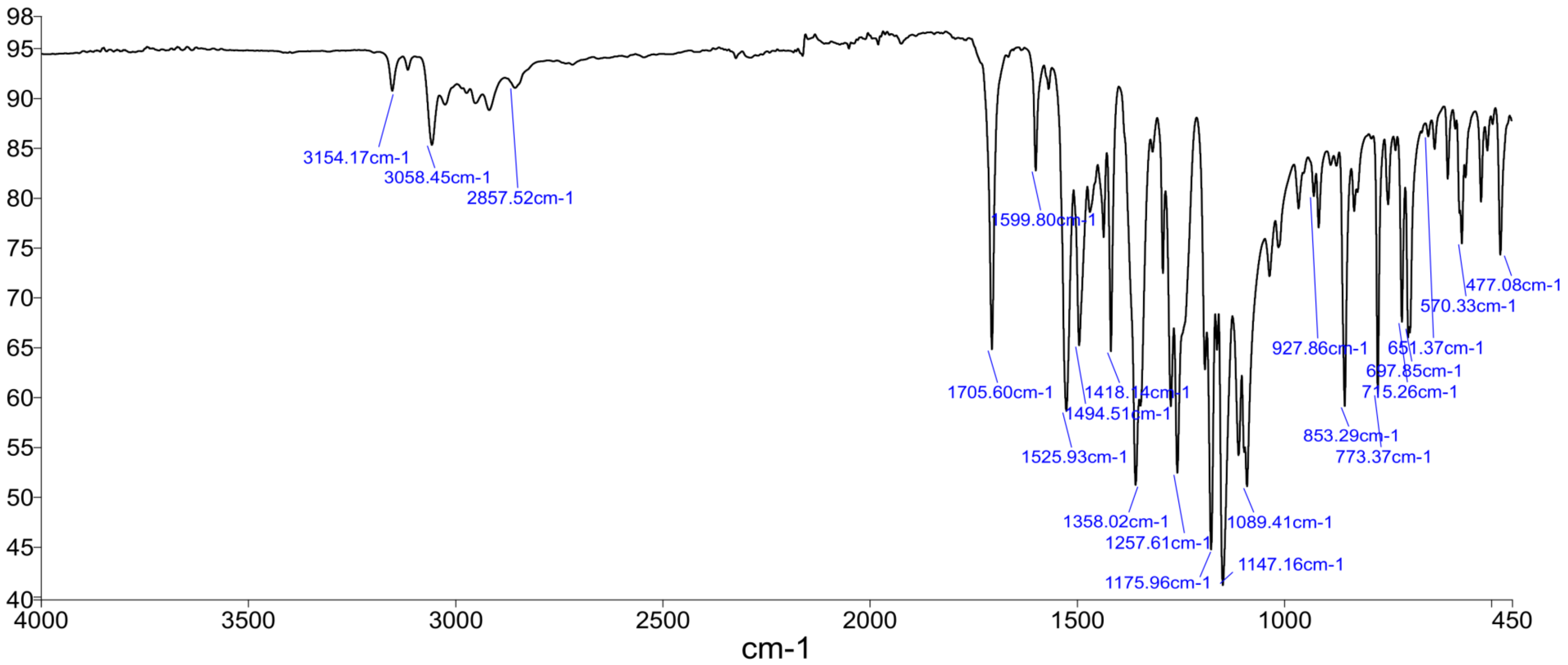

Figure S 14. FT-IR (ATR) spectrum of **S2**.

(E)-4-((1,3-dimesityl-1,3-dihydro-2H-imidazol-2-ylidene)triaz-1-en-1-yl)benzoic acid 4

To **S2** (0.621 g, 1.30 mmol) was added a solution of KOH (1.22 g, 21.8 mmol) in 35 mL methanol. The resulting solution was then refluxed at 80 °C until TLC showed the reaction to be complete (3 hours). The reaction solution was then poured into water (200 mL) and the pH of the aqueous solution was adjusted to pH 4 via the dropwise addition of 2 M HCl (aq) with vigorous stirring, whereupon a yellow precipitate formed. The precipitate was removed via filtration and was then dissolved in EtOAc. The resultant solution was then dried over $MgSO_4$ and was concentrated *in vacuo* to yield **4** as a yellow solid (0.589 g, 97%).

^{1}H-NMR (400 MHz, DMSO-d_6): δ_H 7.57 ($m^{AA'BB'}$, 2H), 7.29 (s, 2H), 7.11 (s, 4H), 6.36 (s, 2H), 2.35 (s, 6H), 2.07 (s, 12H).

^{13}C-NMR (101 MHz, DMSO-d_6): δ_C 167.2, 155.2, 150.5, 138.3, 134.5, 133.9, 129.7, 129.0, 126.6, 120.1, 118.3, 20.7, 17.4.

FT-IR (ATR) (υmax/cm^{-1}): 3086 (C-H stretch, aromatic), 2918 (C-H stretch, alkyl), 1707 (C=O stretching), 1524 (C-C stretch, aromatic), 1359 (C-N stretching), 1176 (C-N stretching), 861 (C-H bend, aromatic), 777 (C-H bend, aromatic).

(ESI)HRMS: Found $[M+H]^+$ 468.2389, $C_{28}H_{29}N_5O_2$ requires 468.2394.

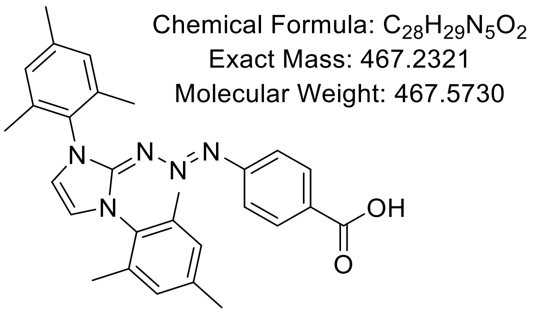

Figure S 15. The structure of (E)-4-((1,3-dimesityl-1,3-dihydro-2H-imidazol-2-ylidene)triaz-1-en-1-yl)benzoic acid **4**.

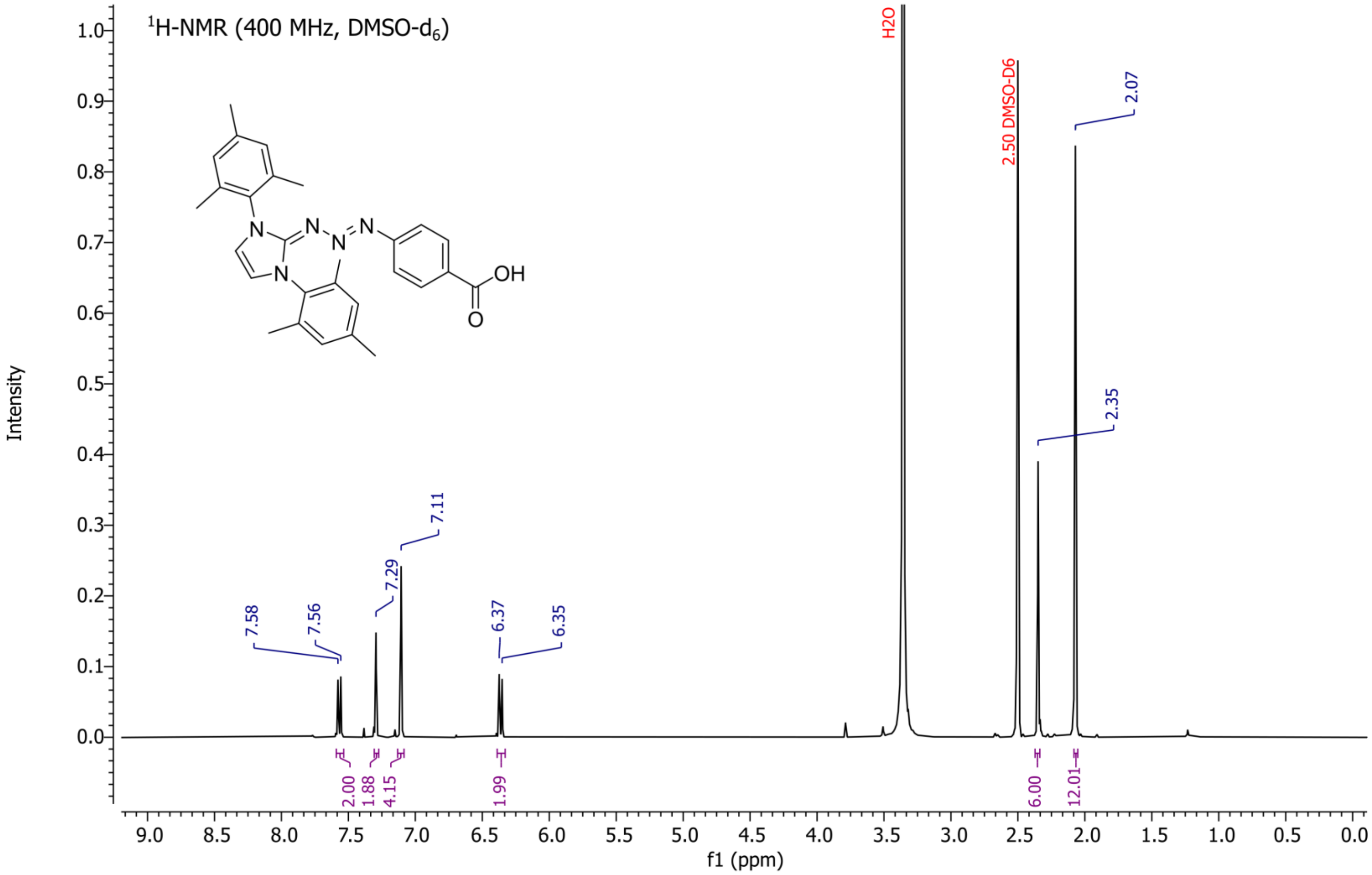

Figure S 16. ^{1}H-NMR spectrum of **4**.

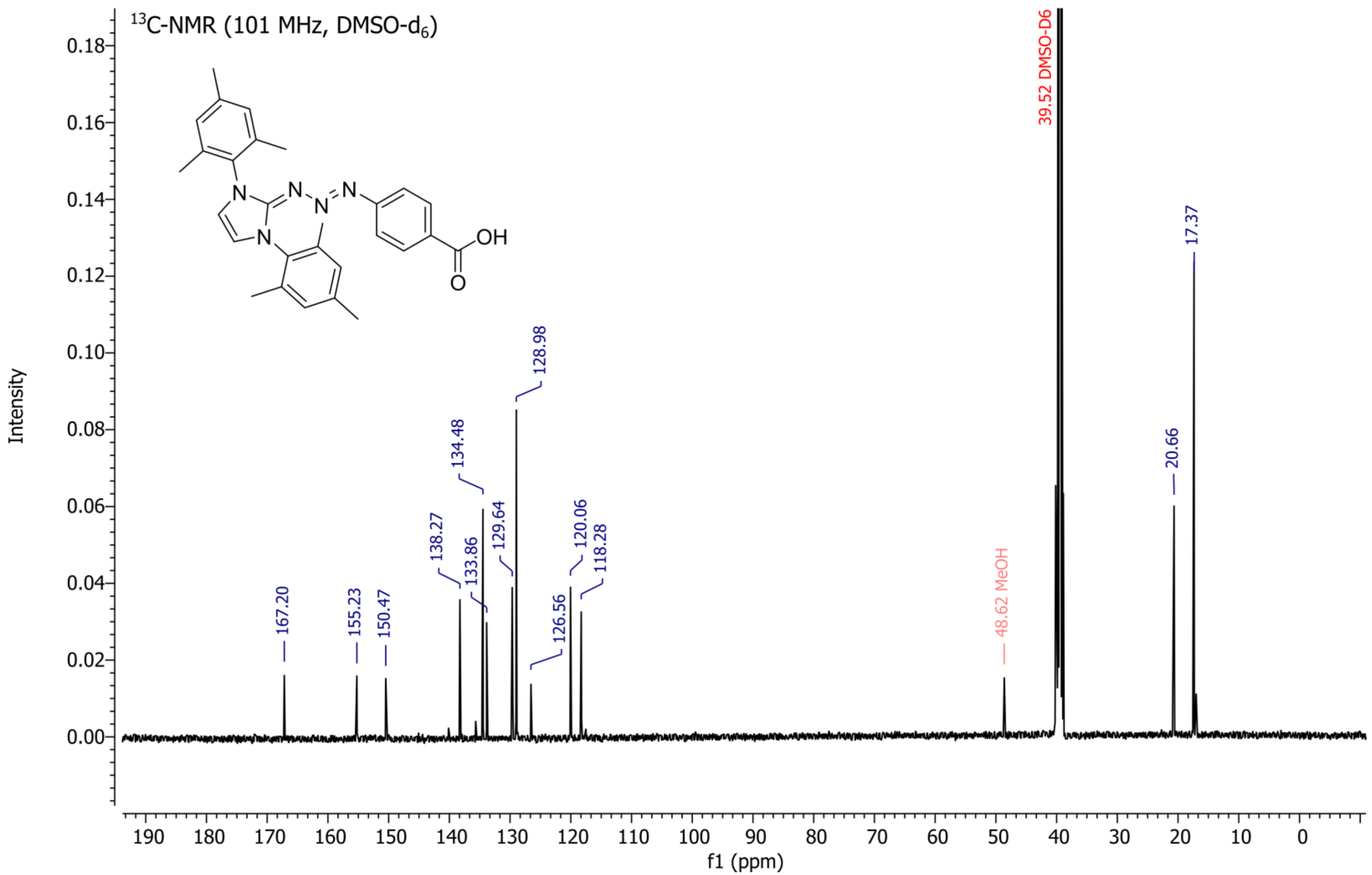

Figure S 17. ^{13}C-NMR spectrum of **4**.

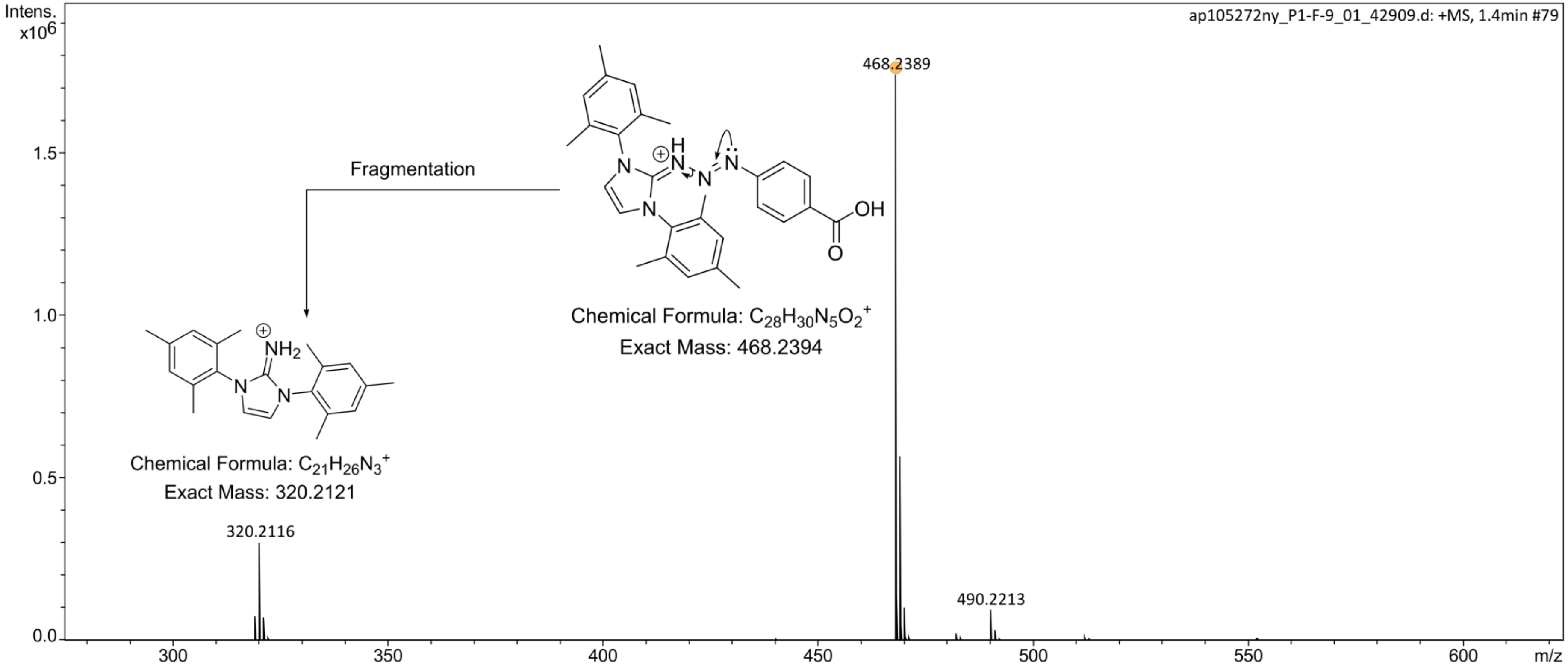

Meas. m/z	#	Ion Formula	m/z	err [ppm]	err [mDa]	mSigma	Mean err [ppm]
468.2389	1	C28H30N5O2	468.2394	1.0	0.5	1.2	0.1

Figure S 18. (ESI)HRMS of **4**.

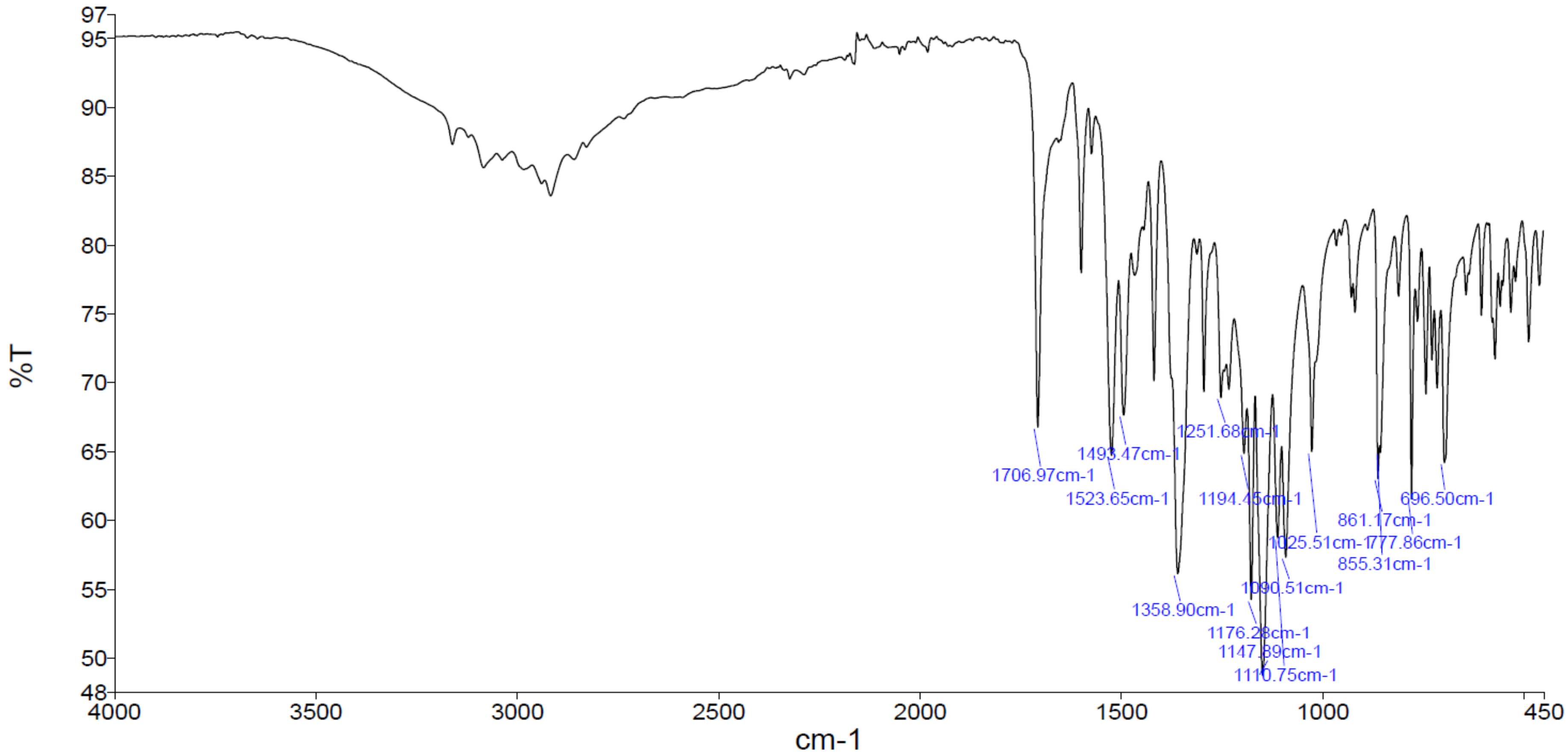

Figure S 19. FT-IR (ATR) spectrum of **4**.

General method of triazabutadiene NHS ester preparation

To a vessel containing **4** (50 mg, 0.107 mmol) and NHS reagent **1**, **2** or **3** (0.11 mmol) was added a solution of DCC (23 mg, 0.111 mmol) in anhydrous DMF (1 mL). The resultant reaction mixture was stirred overnight at rt in a sealed vessel. Insoluble material was then removed via filtration (pushed through a cotton wool plug inside a pipette) and the eluate was concentrated *in vacuo* and a crude NMR was recorded.

The crude material was then further purified via flash silica column chromatography to yield a purified NHS ester product. In cases where triazabutadiene-derived impurity **X** was detected the relative integrals of signals from this impurity compared to those from the desired product were used to calculate how much impurity **X** was present, and this would then be accounted for when calculating the yield of the NHS ester product.

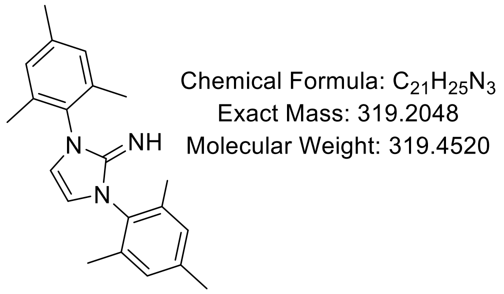

Figure S 20. The structure of triazabutadiene-derived impurity **X**.

2,5-dioxopyrrolidin-1-yl (E)-4-((1,3-dimesityl-1,3-dihydro-2H-imidazol-2-ylidene)triaz-1-en-1-yl)benzoate 5

Prepared using NHS reagent **1**. Silica flash column chromatography was performed using a gradient from hexane → ethyl acetate, yielding **5** as a yellow/orange foamy solid (0.349 g, 69%). Spectral characterisations were consistent with those which we have previously reported.[5]

^{1}H-NMR (400 MHz, DMF-d$_7$): δ_H 7.79 (m$^{AA'BB'}$, 2H), 7.43 (s, 2H), 7.16 (s, 4H), 6.60 (m$^{AA'BB'}$, 2H), 3.00 (s, 4H), 2.41 (s, 6H), 2.17 (s, 12H).

^{13}C-NMR (101 MHz, DMF-d$_7$): δ_c 170.8, 162.2, 158.5, 151.3, 139.1, 135.1, 134.4, 131.0, 129.6, 121.3, 120.2, 119.0, 26.0, 20.6, 17.4.

FT-IR (ATR) (υmax/cm^{-1}): 2920 (C-H stretch, alkyl), 1760 (C=O stretching), 1736 (C=O stretching), 1597 (C-C stretch, aromatic), 1523 (C-C stretch, aromatic), 1353 (C-N stretching), 1183 (C-O stretch, ester), 1142 (C-N stretching), 855 (C-H bend, aromatic).

(ESI)HRMS: Found [M+H]$^+$ 565.2562, $C_{32}H_{33}N_6O_4$ requires 565.2558.

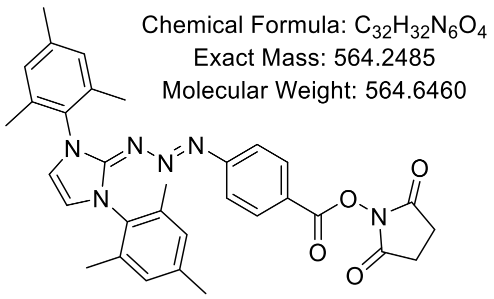

Figure S 21. The structure of 2,5-dioxopyrrolidin-1-yl (E)-4-((1,3-dimesityl-1,3-dihydro-2H-imidazol-2-ylidene)triaz-1-en-1-yl)benzoate **5**.

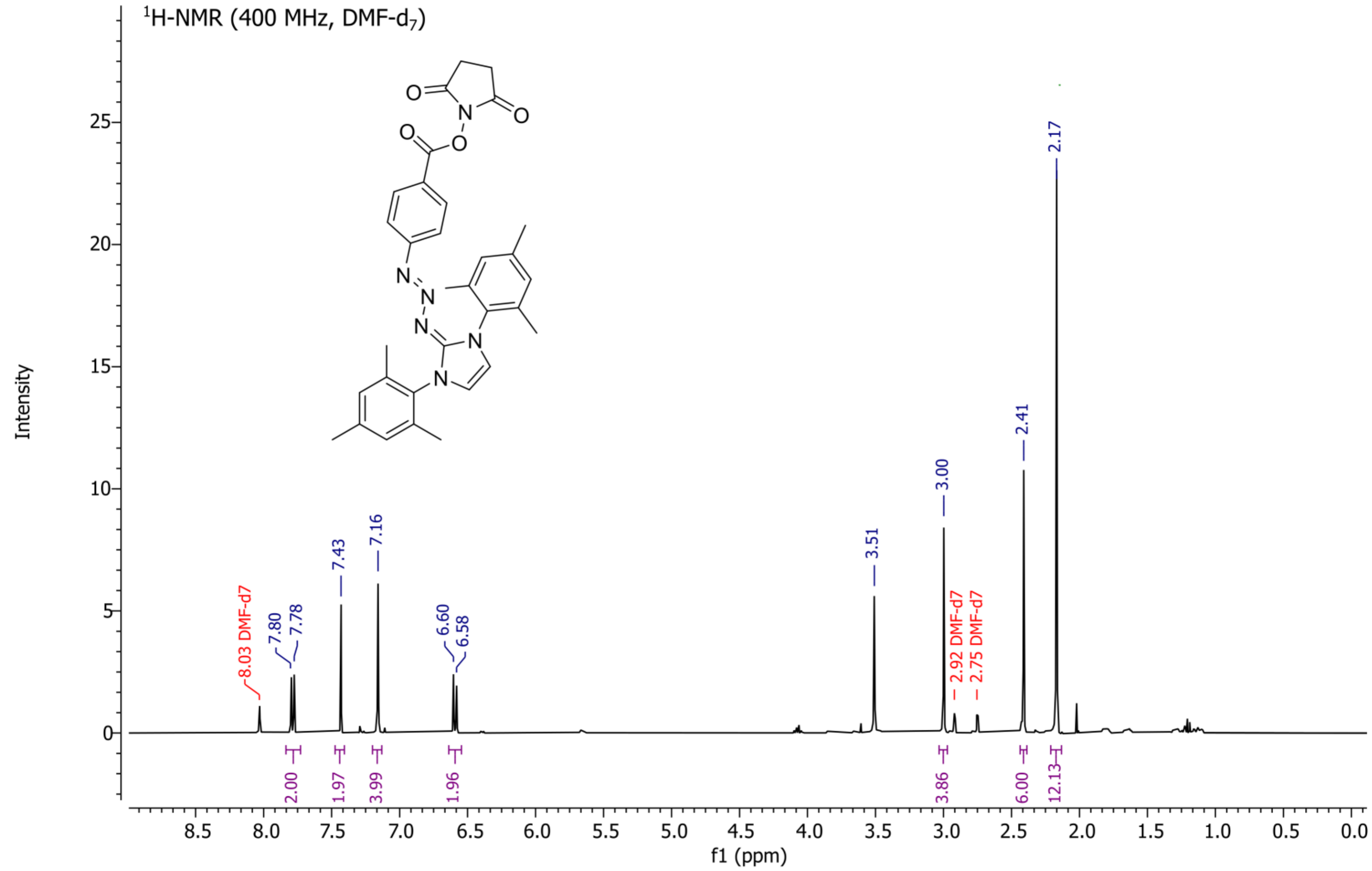

Figure S 22. ^{1}H-NMR spectrum of **5**.

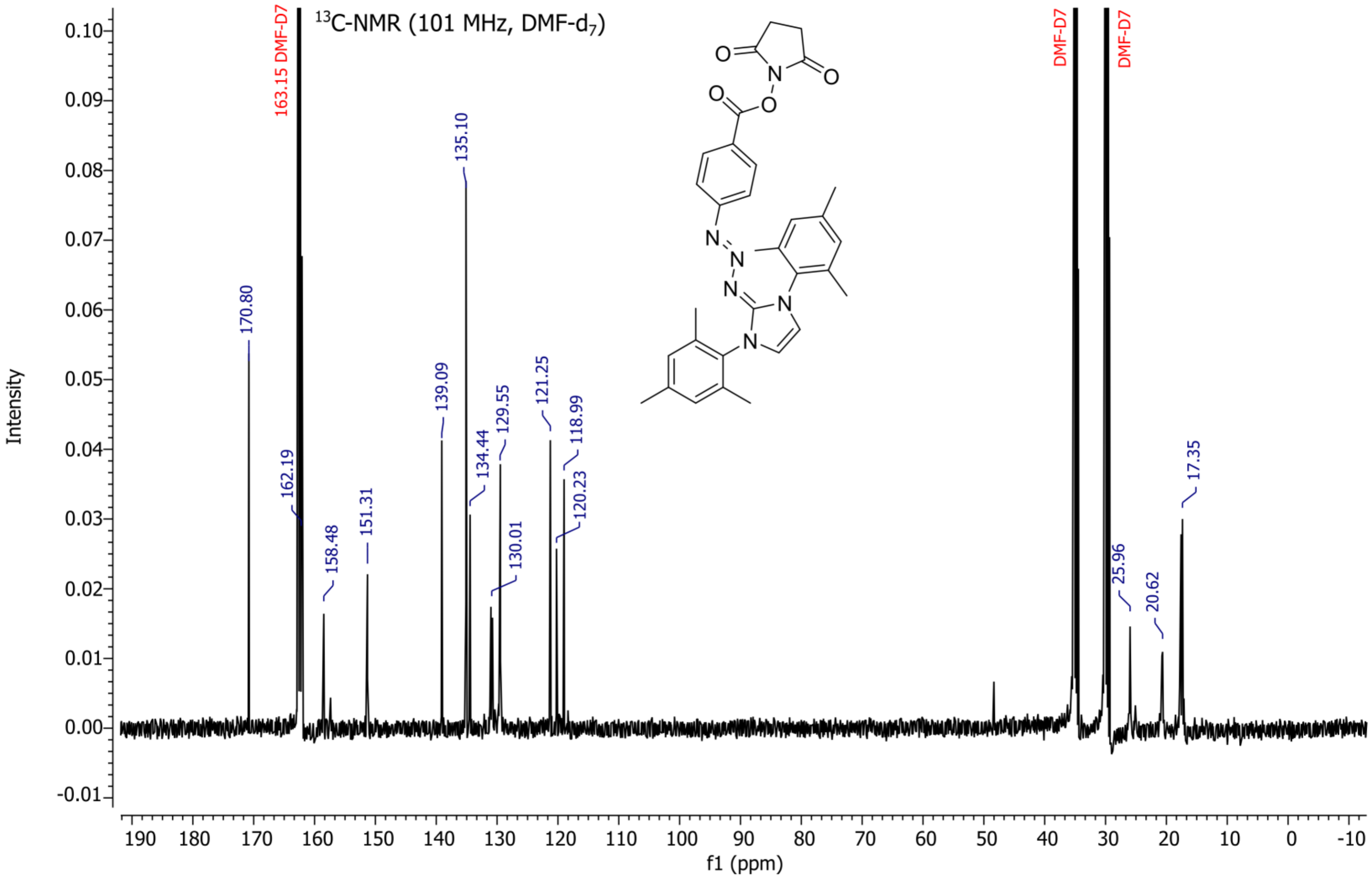

Figure S 23. ^{13}C-NMR spectrum of **5**.

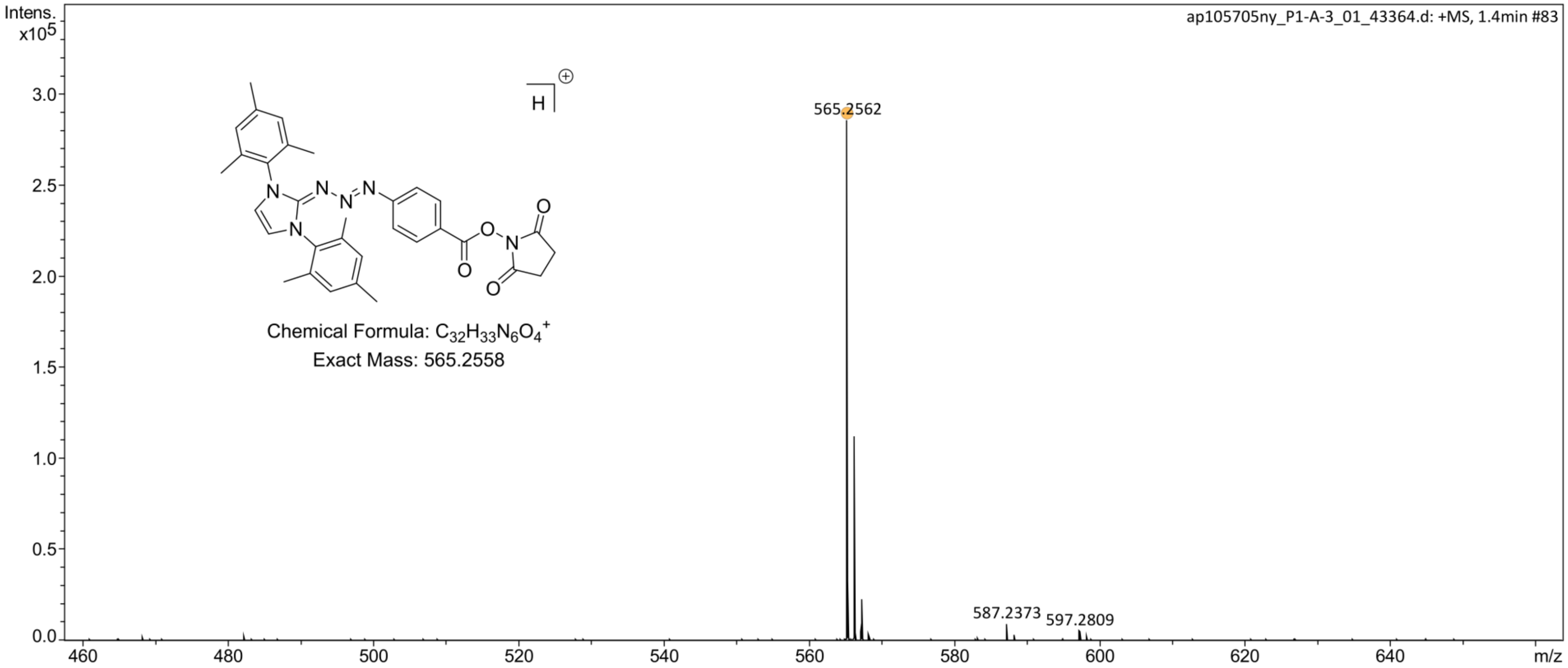

Meas. m/z	#	Ion Formula	m/z	err [ppm]	err [mDa]	mSigma	Mean err [ppm]
565.2562	1	C32H33N6O4	565.2558	-0.8	-0.4	9.4	-0.6

Figure S 24. (ESI)HRMS of **5**.

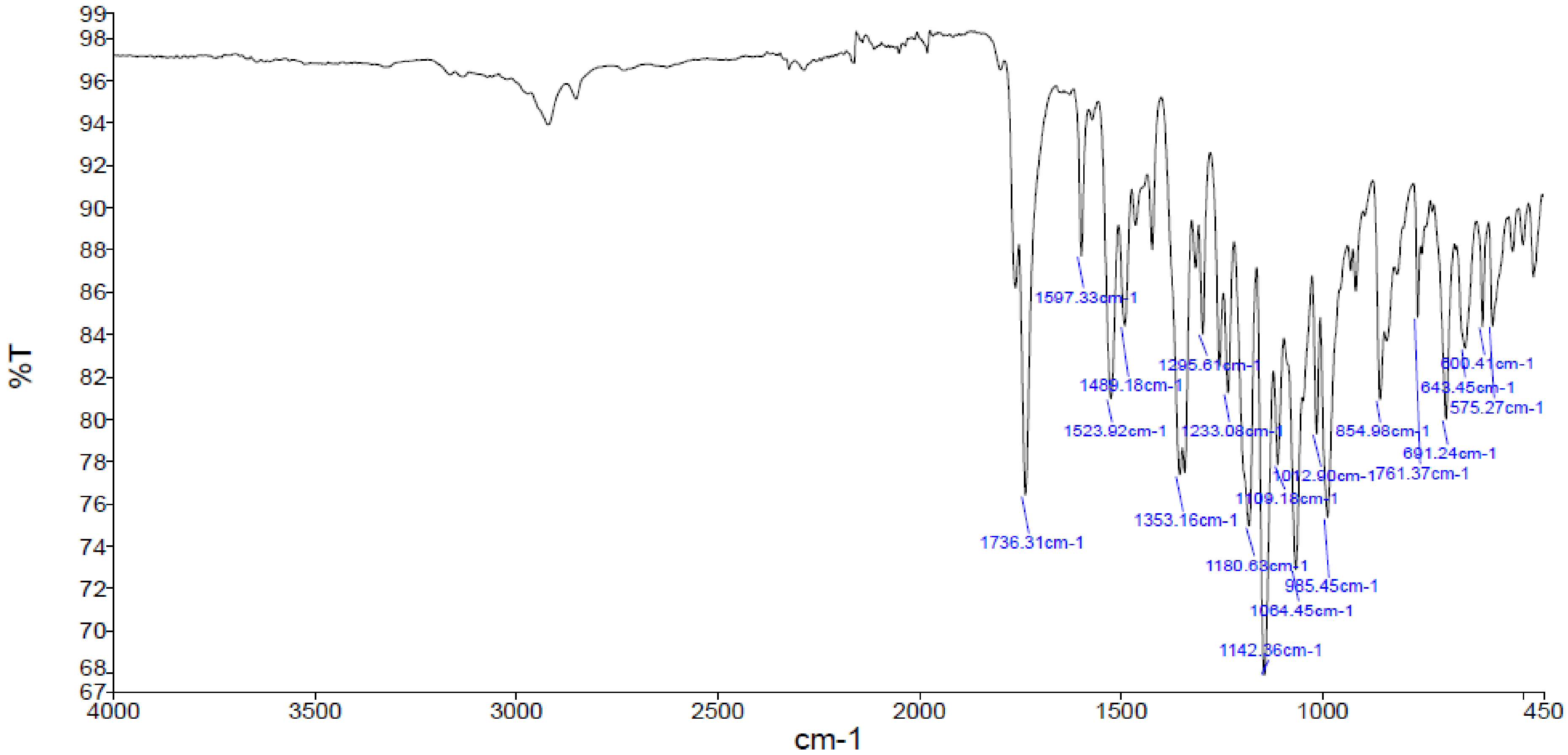

Figure S 25. FT-IR (ATR) spectrum of **5**.

(E)-1-((4-((1,3-dimesityl-1,3-dihydro-2H-imidazol-2-ylidene)triaz-1-en-1-yl)benzoyl)oxy)-2,5-dioxopyrrolidine-3-sulfonate sodium salt 6

Prepared using NHS reagent **2**. Silica flash column chromatography was performed using a gradient from DCM → 40% MeOH, yielding an orange solid (16.5 mg). NMR of this solid showed it to be primarily comprised of the desired Sulfo-NHS ester **6**, but peaks attributable to triazabutadiene derived impurity **X** were also visible. It could be calculated from the ratio of the ^{1}H-NMR peak integrals that the isolated material contained around 13.5 mg of the desired ester product and around 2.9 mg of triazabutadiene-derived impurity. The yield of **6** was thus ~13.5 mg, (~19%).

^{1}H-NMR (400 MHz, DMF-d$_7$): δ_H 7.81 (m$^{AA'BB'}$, 2H), 7.50 (s, 2H), 7.17 (s, 4H), 6.61 (m$^{AA'BB'}$, 2H), 4.11 (dd, *J* = 8.60, 2.50 Hz, 1H), 3.31 (dd, *J* = 18.20, 8.60 Hz, 1H), 3.16 (dd, *J* = 18.20, 2.50 Hz, 1H), 2.40 (s, 6H), 2.17 (s, 12H).

^{13}C-NMR (101 MHz, DMF-d$_7$): δ_C 170.4, 167.2, 158.0, 151.5, 140.0, 135.8, 135.0, 131.8, 130.3, 121.6, 120.1, 118.7, 57.9, 32.5, 21.4, 18.2.

FT-IR (ATR) (υmax/cm^{-1}): 2922 (C-H stretch, alkyl), 1739 (C=O stretching), 1598 (C-C stretch, aromatic), 1521 (C-C stretch, aromatic), 1355 (C-N stretching), 1187 (C-O stretch, ester), 1146 (C-N stretching), 855 (C-H bend, aromatic).

(ESI)HRMS: Found [M-H]$^-$ 643.1980, $C_{32}H_{31}N_6O_7S^-$ requires 643.1982.

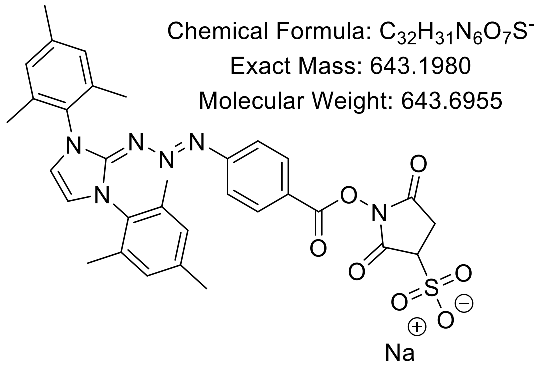

Figure S 26. The structure of (E)-1-((4-((1,3-dimesityl-1,3-dihydro-2H-imidazol-2-ylidene)triaz-1-en-1-yl)benzoyl)oxy)-2,5-dioxopyrrolidine-3-sulfonate sodium salt **6**.

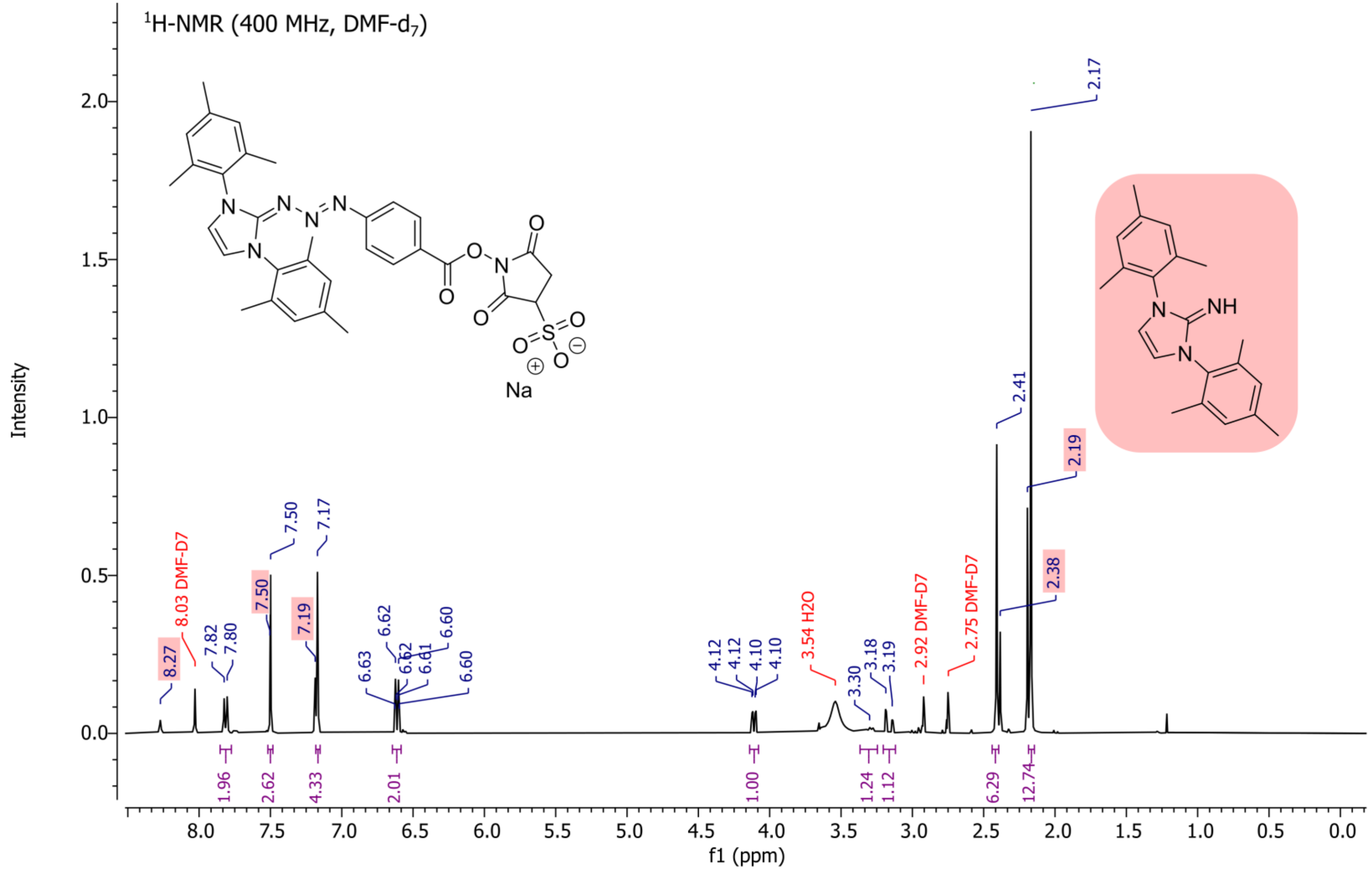

Figure S 27. ^{1}H-NMR spectrum of **6**.

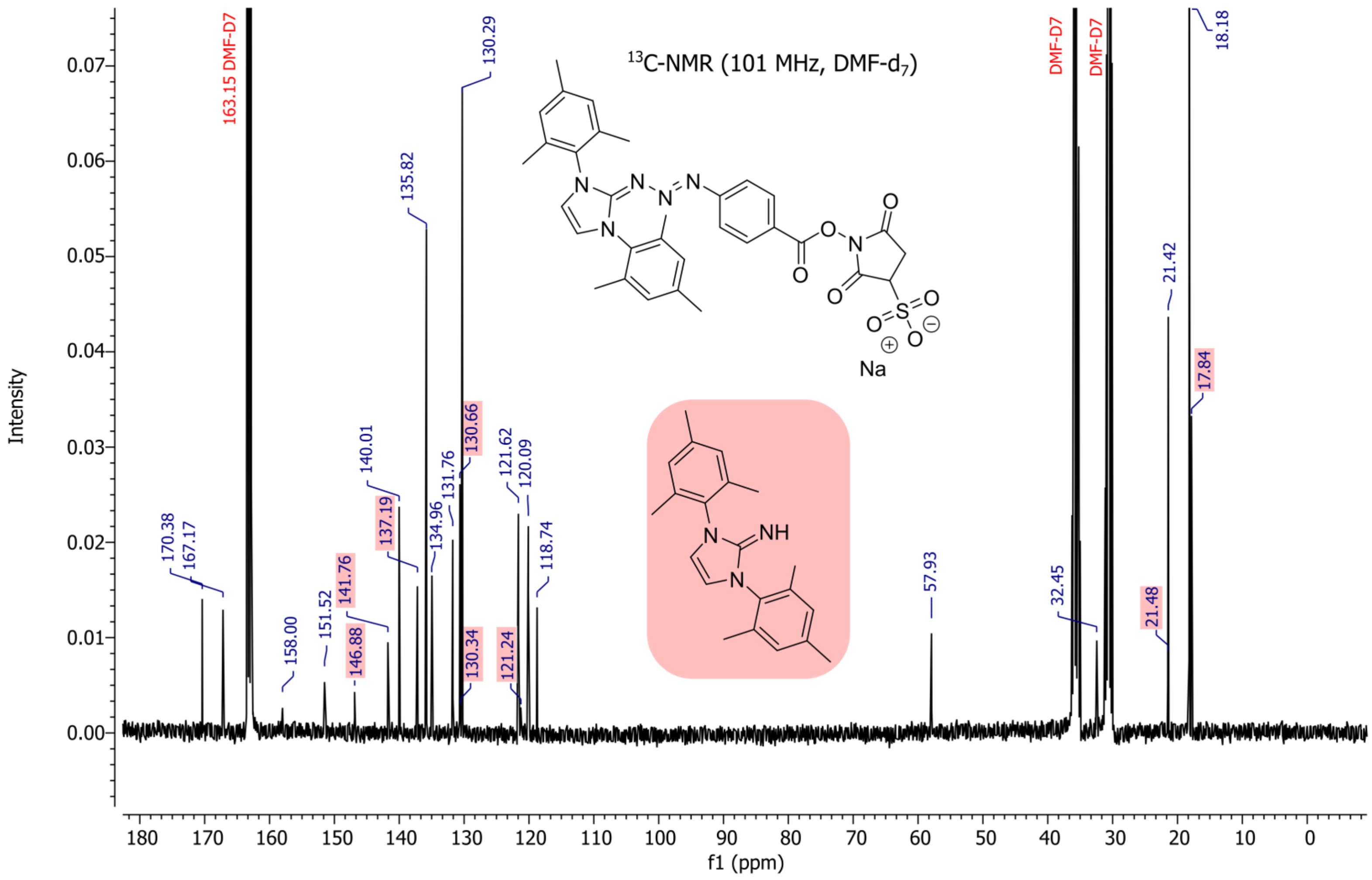

Figure S 28. ^{13}C-NMR spectrum of **6**.

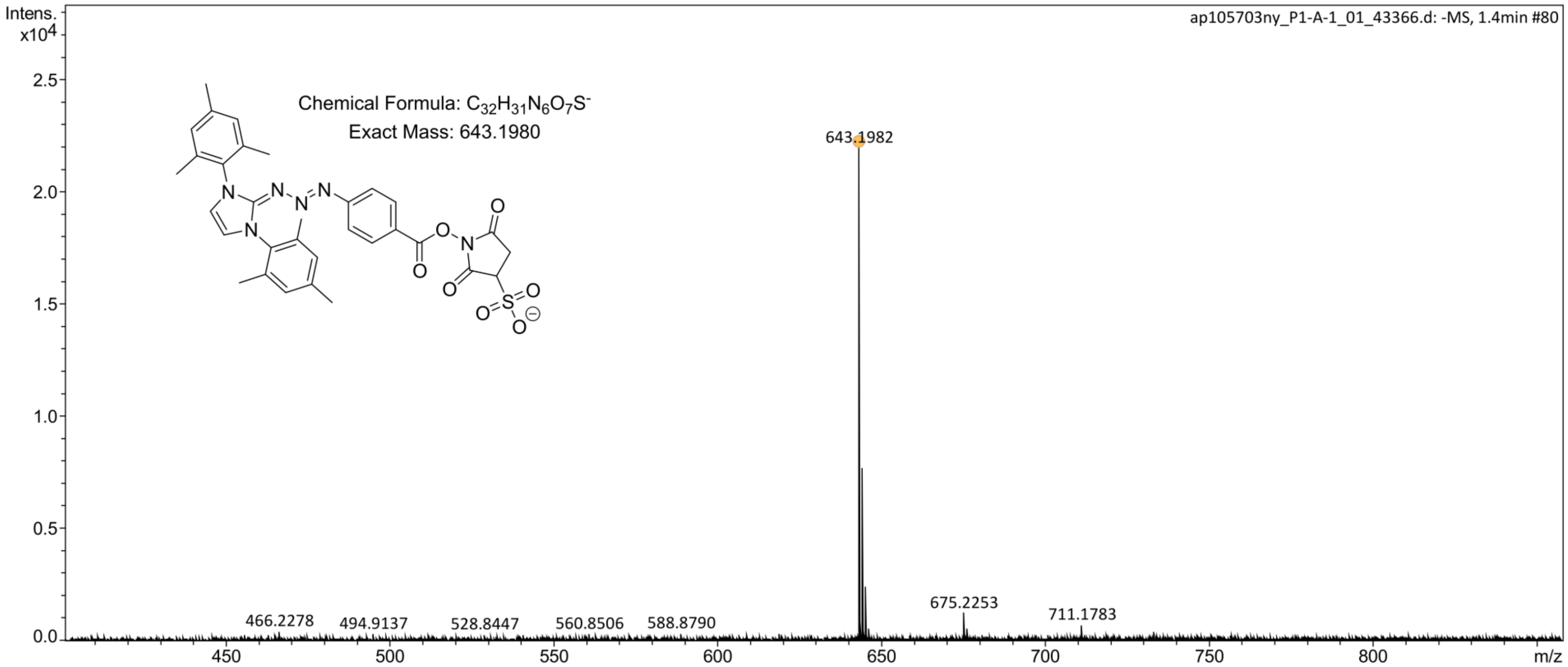

Meas. m/z	#	Ion Formula	m/z	err [ppm]	err [mDa]	mSigma	Mean err [ppm]
643.1982	1	C32H31N6O7S	643.1980	-0.2	-0.1	19.2	-0.8

Figure S 29. (ESI)HRMS of **6**.

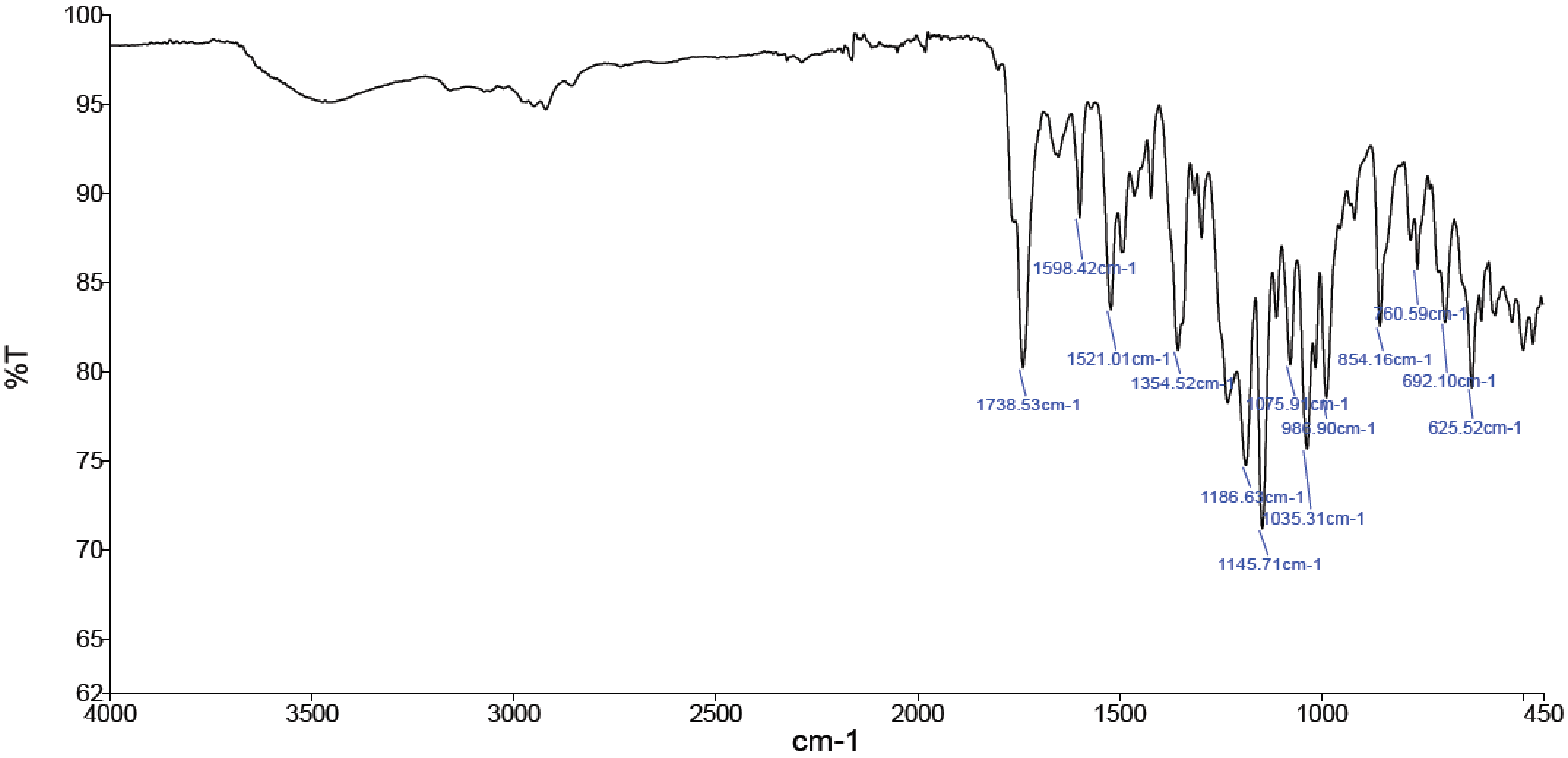

Figure S 30. FT-IR (ATR) spectrum of **6**.

(E)-1-((4-((1,3-dimesityl-1,3-dihydro-2H-imidazol-2-ylidene)triaz-1-en-1-yl)benzoyl)oxy)-2,5-dioxopyrrolidine-3-sulfonate sodium salt 15-crown-5 complex 7

Prepared using NHS reagent **3**. Silica flash column chromatography was performed using a gradient from DCM → 40% MeOH, yielding an orange oil that solidified over time (59 mg). NMR of this solid showed it to be primarily comprised of the desired Na Sulfo-NHS ester 15-crown-5 complex **7**, but a very small amount of triazabutadiene-derived impurity **X** was also detected. Using the relative integrals in the ^{1}H-NMR spectrum it could be calculated that the yield of Na Sulfo-NHS ester 15-crown-5 complex **7** was 54 mg (57%).

^{1}H-NMR (400 MHz, DMF-d$_7$): δ_H 7.81 (m$^{AA'BB'}$, 2H), 7.46 (s, 2H), 7.16 (s, 4H), 6.61 (m$^{AA'BB'}$, 2H), 4.13 (dd, *J* = 8.80, 2.40 Hz, 1H), 3.67 (s, 20H), 3.32 (dd, *J* = 18.30, 8.80 Hz, 1H), 3.16 (dd, *J* = 18.30, 2.40 Hz, 1H), 2.41 (s, 6H), 2.17 (s, 12H).

^{13}C-NMR (101 MHz, DMF-d$_7$): δ_C 170.4, 167.2, 158.6, 151.7, 139.9, 135.8, 135.0, 131.7, 130.3, 121.8, 119.9, 118.7, 57.9, 32.4, 21.4, 18.2.

FT-IR (ATR) (υmax/cm^{-1}): 2918 (C-H stretch, alkyl), 1739 (C=O stretching), 1598 (C-C stretch, aromatic), 1521 (C-C stretch, aromatic), 1352 (C-N stretching), 1187 (C-O stretch, ester), 1145 (C-N stretching), 856 (C-H bend, aromatic).

(ESI)HRMS: Found 667.1973, $C_{32}H_{32}NaN_6O_7S^+$ requires 667.1945. Found 243.1208, $C_{10}H_{20}NaO_5^+$ requires 243.1203.

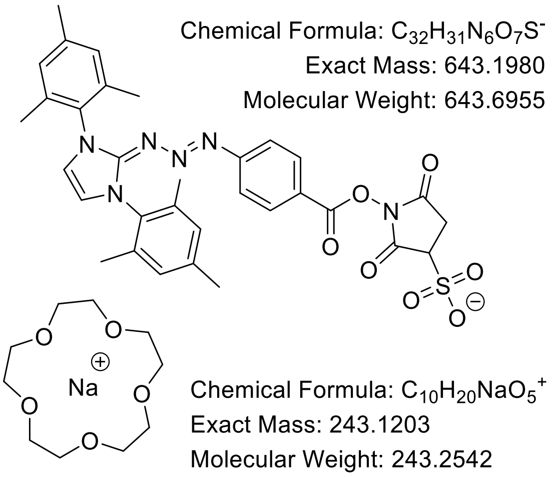

Figure S 31. The structure of (E)-1-((4-((1,3-dimesityl-1,3-dihydro-2H-imidazol-2-ylidene)triaz-1-en-1-yl)benzoyl)oxy)-2,5-dioxopyrrolidine-3-sulfonate sodium salt 15-crown-5 complex **7**.

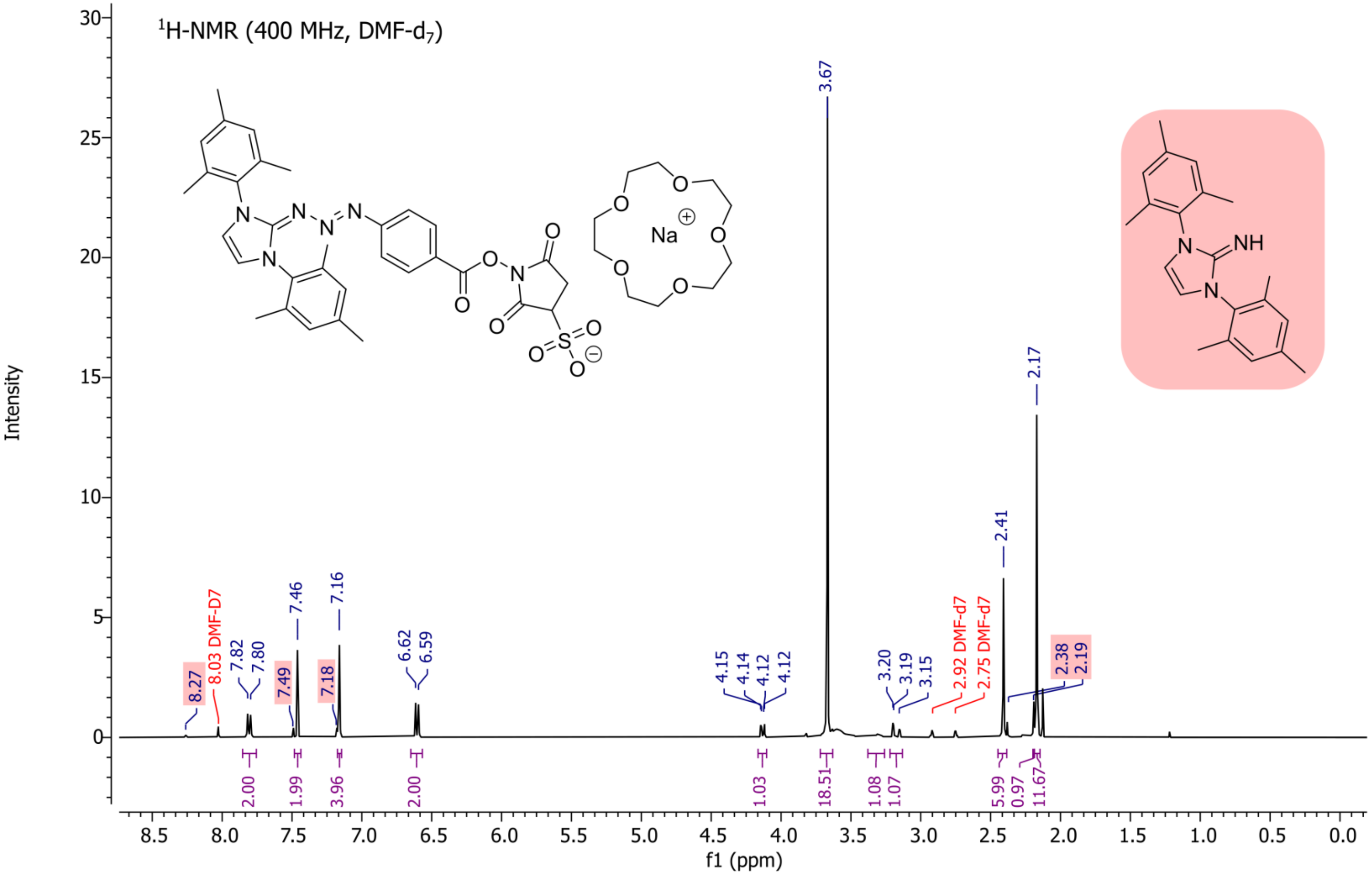

Figure S 32. ^{1}H-NMR spectrum of **7**.

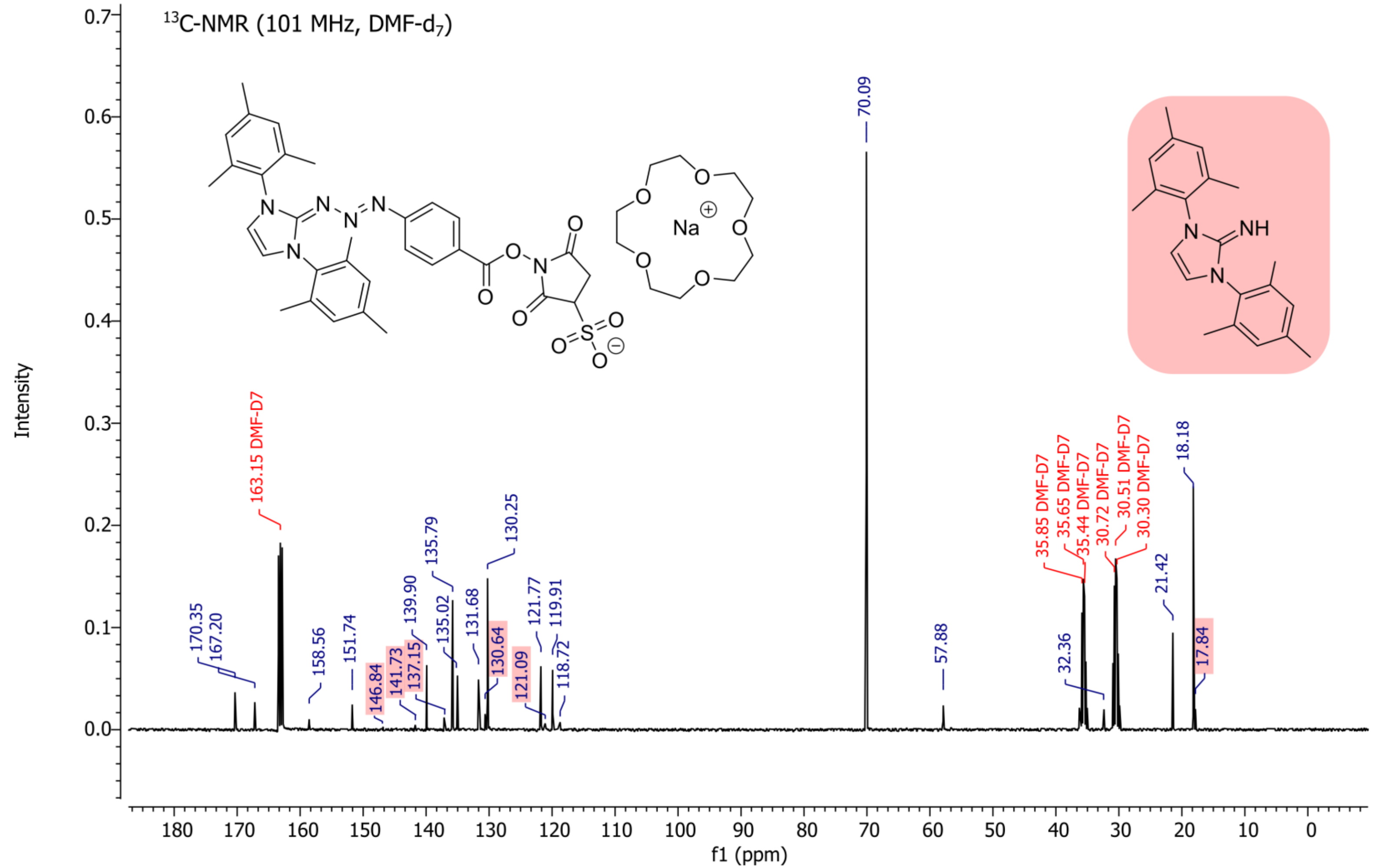

Figure S 33. ^{13}C-NMR spectrum of **7**.

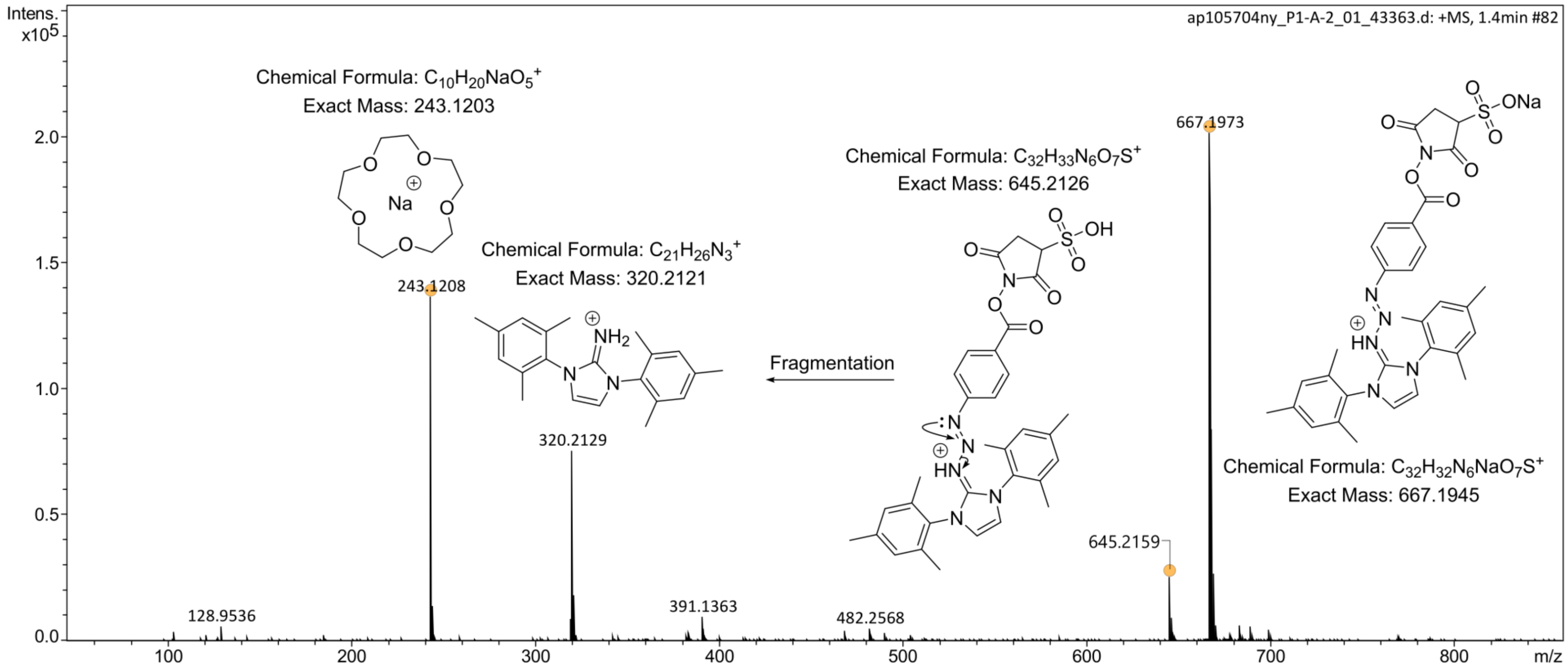

Meas. m/z	#	Ion Formula	m/z	err [ppm]	err [mDa]	mSigma	Mean err [ppm]
243.1208	1	C10H20NaO5	243.1203	-1.9	-0.5	6.4	-3.8
645.2159	1	C32H33N6O7S	645.2126	-5.1	-3.3	16.1	-4.3
667.1973	1	C32H32N6NaO7S	667.1945	-4.1	-2.7	17.7	-5.1

Figure S 34. (ESI)HRMS of **7**.

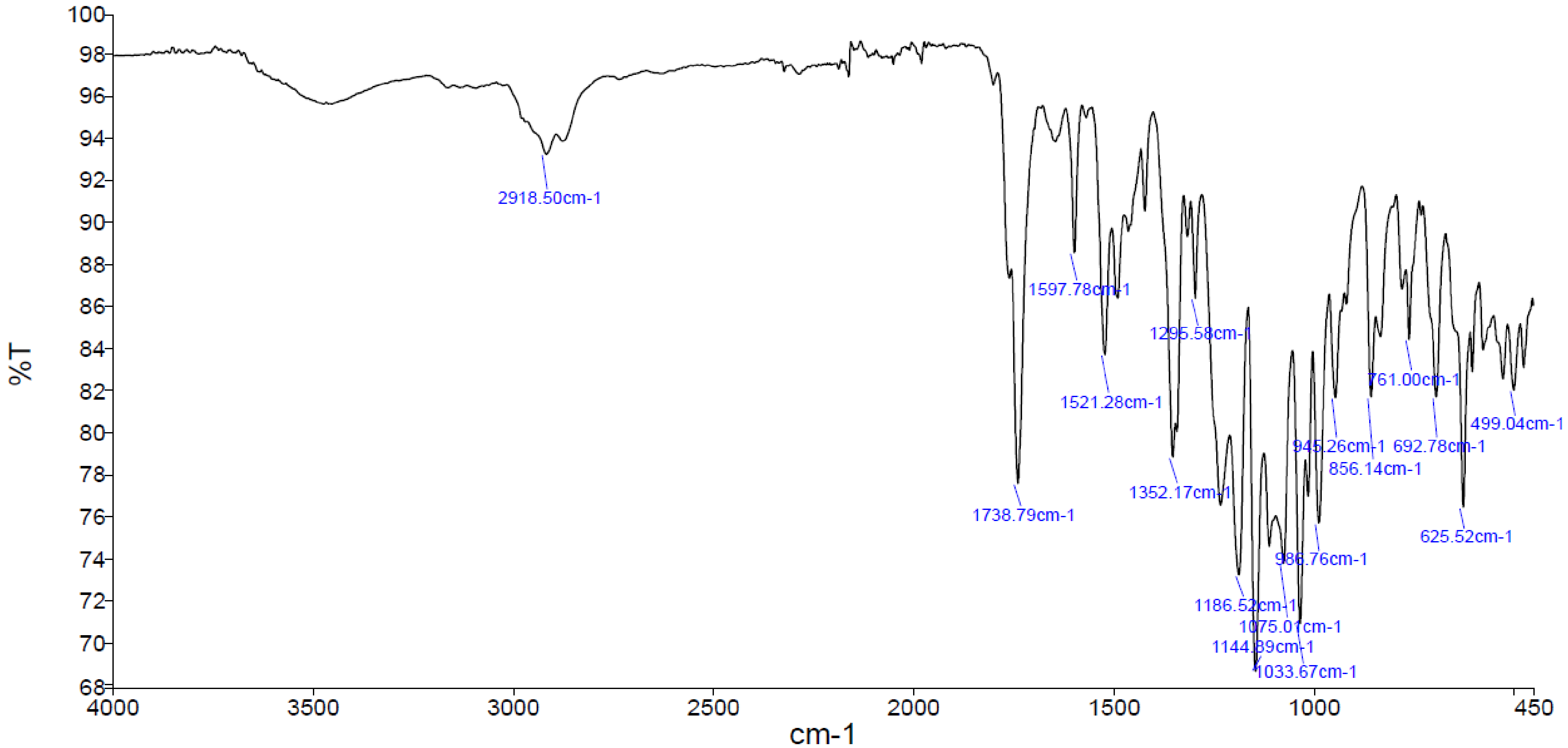

Figure S 35. FT-IR (ATR) spectrum of **7**.

Derivatives of isobutyric acid

Sodium 1-(isobutyryloxy)-2,5-dioxopyrrolidine-3-sulfonate 8

To a vessel containing commercially available *N*-hydroxysulfosuccinimide sodium salt **2** (0.108 g, 0.50 mmol) and isobutyric anhydride (0.5 mL, 3.00 mmol) was added DMSO (2.5 mL). The reaction mixture was heated to 90°C and agitated, whereupon the solid of **2** disappeared. The reaction solution was then incubated at to 90°C overnight. Upon reaction completion the reaction mixture was diluted with dioxane such that the DMSO content was less than 5% v/v. The resultant solution was flash-frozen and lyophilized which yielded a residue of **S4** and DMSO. In order to remove the residual DMSO, this residue was once again dissolved in dioxane, flash-frozen and lyophilized. This yielded **S4** as a white solid (0.119 g, 83%).

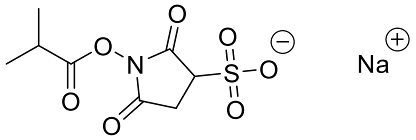

Chemical Formula: $C_8H_{10}NO_7S^-$
Exact Mass: 264.0183

Figure S 36. The structure of **8**.

¹H-NMR (400 MHz, DMSO-d$_6$): δ_H 3.96 (br d, *J* = 6.96 Hz, 1H), 3.55 (s, 20H), 3.25-3.04 (m, 1H), 2.93 (spet, *J* = 6.96 Hz, 1H), 2.84 (dd, *J* = 18.3, 2.24 Hz, 1H), 1.25-1.19 (m, 6H).

^{13}C-NMR (101 MHz, DMSO-d$_6$): δ_c 172.0, 168.8, 165.5, 56.3, 31.0, 30.9, 18.6.

FT-IR (ATR) (υmax/cm^{-1}): 2981 (C-H stretch, alkyl), 1813 (C=O stretching), 1786 (C=O stretching), 1731 (C=O stretching), 1218 (C-O stretch, ester).

(ESI)HRMS: Found 264.0171, $C_8H_{10}NO_7S^-$ requires 264.0183.

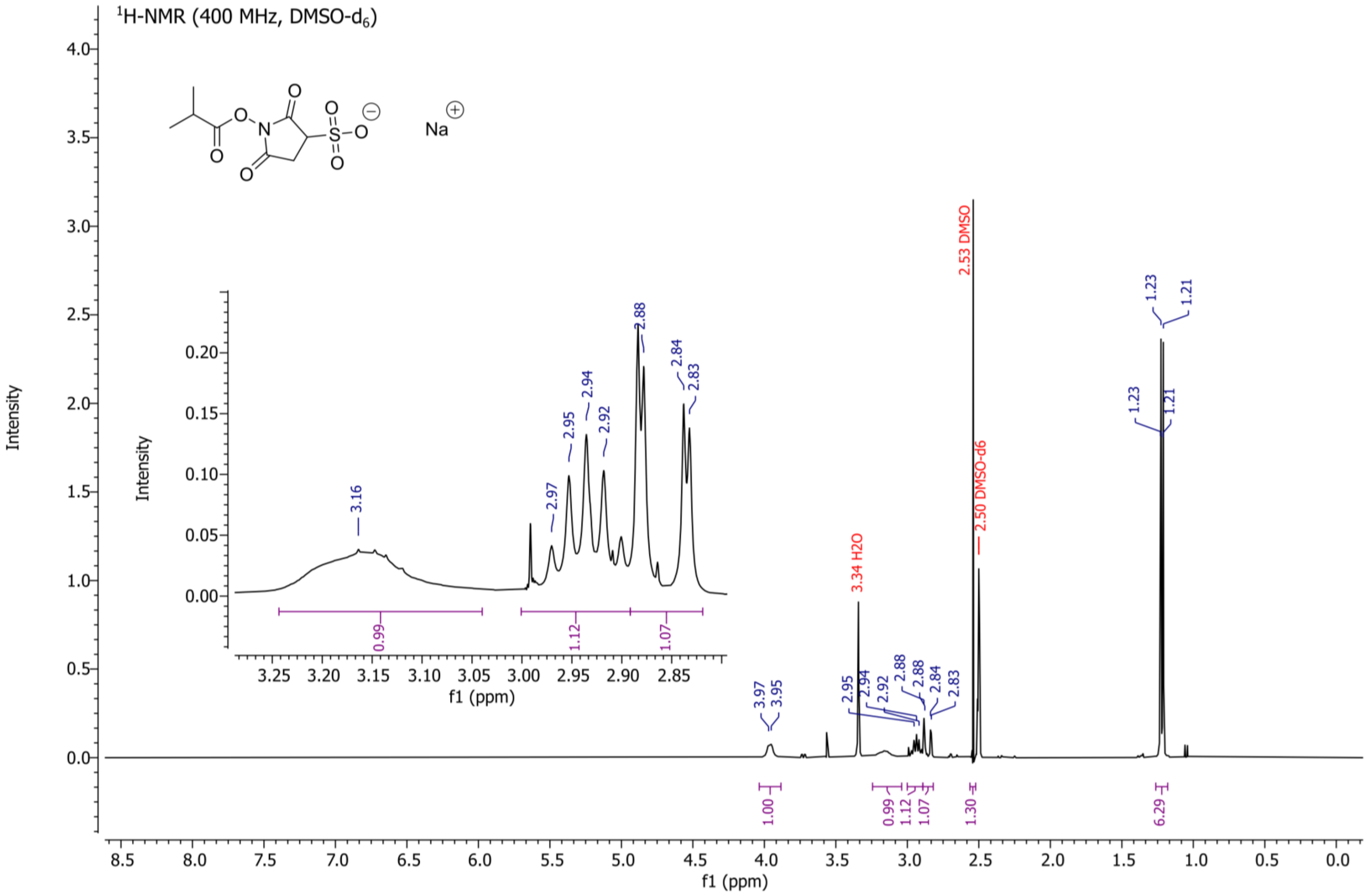

Figure S 37. ^{1}H-NMR spectrum of **8**.

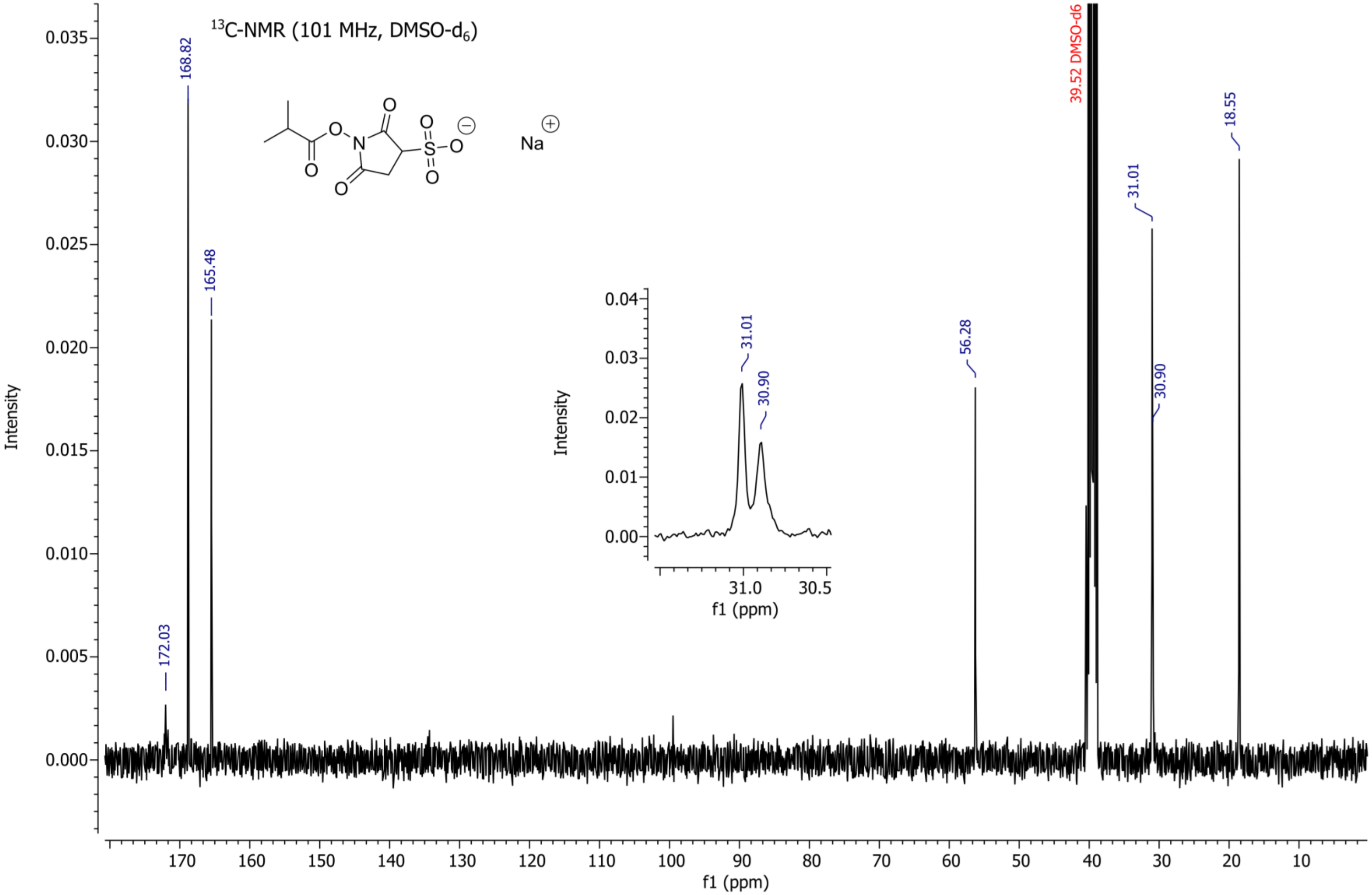

Figure S 38. ^{13}C-NMR spectrum of **8**.

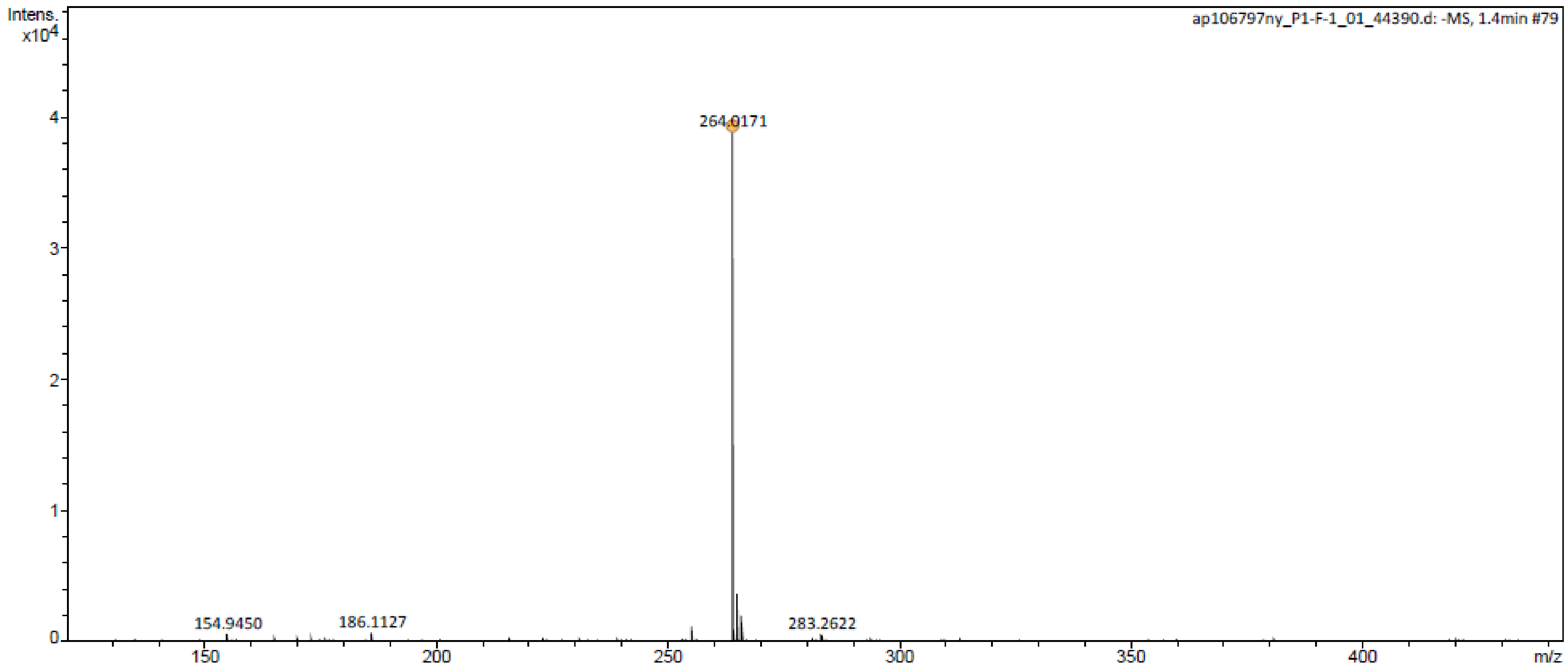

Meas. m/z	#	Ion Formula	m/z	err [ppm]	err [mDa]	mSigma	Mean err [ppm]
264.0171	1	C8H10NO7S	264.0183	4.8	1.3	8.8	3.3

Figure S 39. Negative-mode (ESI)HRMS of **8**.

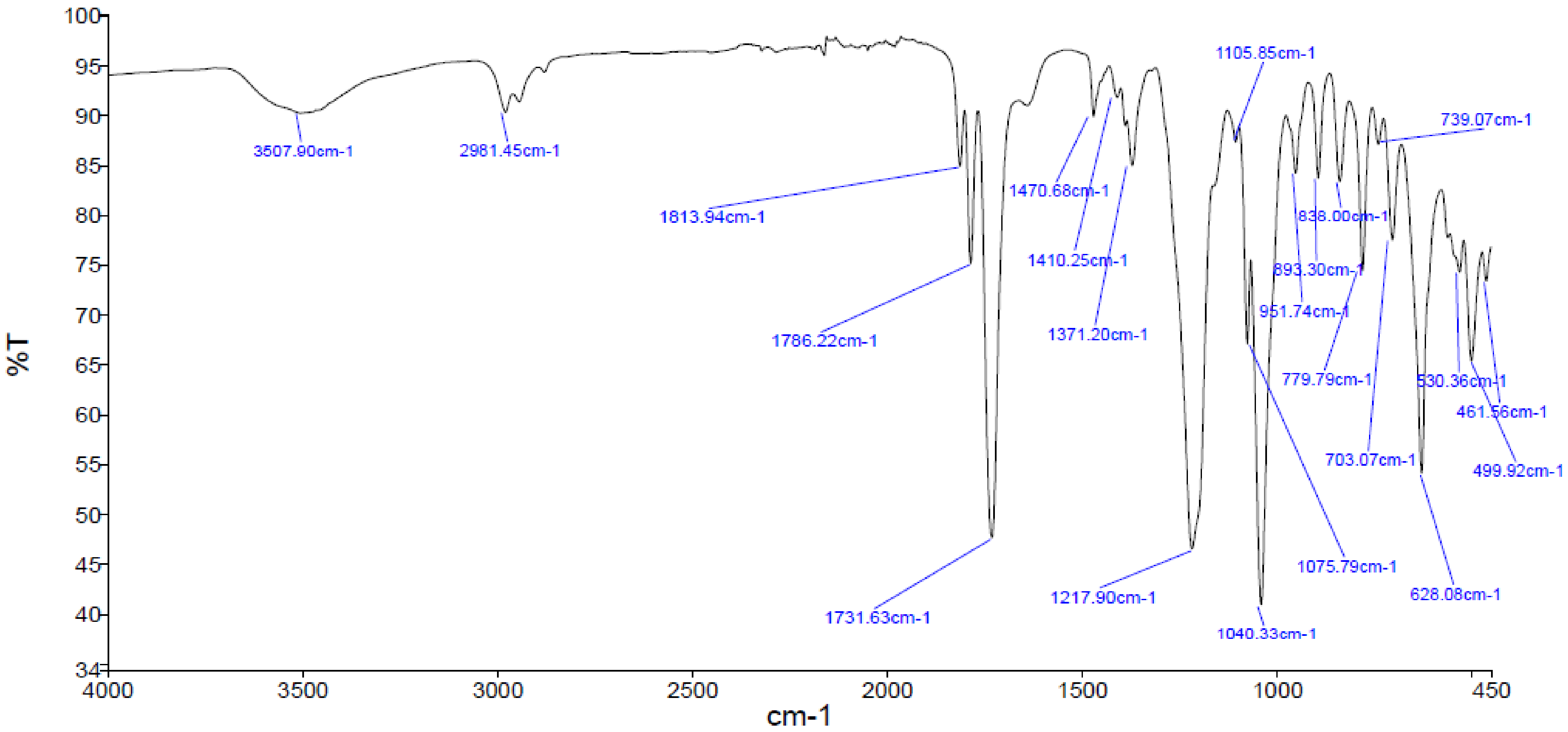

Figure S 40. FT-IR (ATR) spectrum of **8**.

Sodium 1-(isobutyryloxy)-2,5-dioxopyrrolidine-3-sulfonate 15-crown-5 complex 9

To a vessel containing **3** (0.120 g, 0.275 mmol) and isobutyric anhydride (0.25 mL, 1.51 mmol) was added DMF (2.5 mL). Shortly after the dissolution of the reaction components, the reaction mixture solidified. In order to ensure the reaction reached completion, the reaction mixture was heated at 90°C overnight, at which temperature it remained a liquid. Upon reaction completion the still-warm reaction mixture was swiftly added dropwise to a solution of diethyl ether, whereupon a white precipitate formed. This precipitate was isolated, and was subsequently dissolved/suspended in dioxane. The resultant solution was flash-frozen and lyophilized, which yielded **S3** as a white solid in quantitative yield.

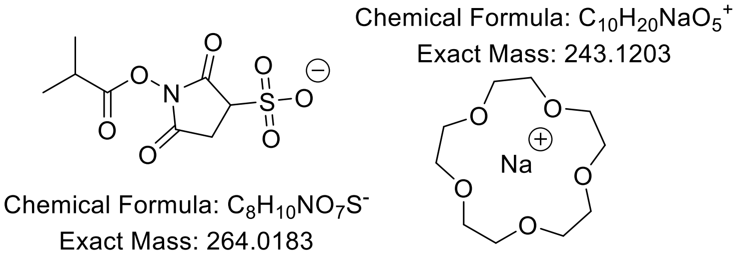

Figure S 41. The structure of **9**.

^{1}H-NMR (400 MHz, DMSO-d$_6$): δ_H 3.93 (br d, *J* = 5.97 Hz, 1H), 3.55 (s, 20H), 3.23-3.06 (m, 1H), 2.94 (spet, *J* = 7.01 Hz, 1H), 2.84 (dd, *J* = 18.3, 2.08 Hz, 1H), 1.25-1.19 (m, 6H).

^{13}C-NMR (101 MHz, DMSO-d$_6$): δ_c 172.0, 168.8, 165.4, 69.0, 56.3, 31.0, 30.9, 18.5.

FT-IR (ATR) (umax/cm^{-1}): 2981 (C-H stretch, alkyl), 2912 (C-H stretch, alkyl), 2884 (C-H stretch, alkyl), 1814 (C=O stretching), 1777 (C=O stretching), 1731 (C=O stretching), 1219 (C-O stretch, ester), 1092 (C-O stretch, ether).

(ESI)HRMS: Found 264.0191, $C_8H_{10}NO_7S^-$ requires 264.0183. Found 243.1199, $C_{10}H_{20}NaO_5^+$ requires 243.1203.

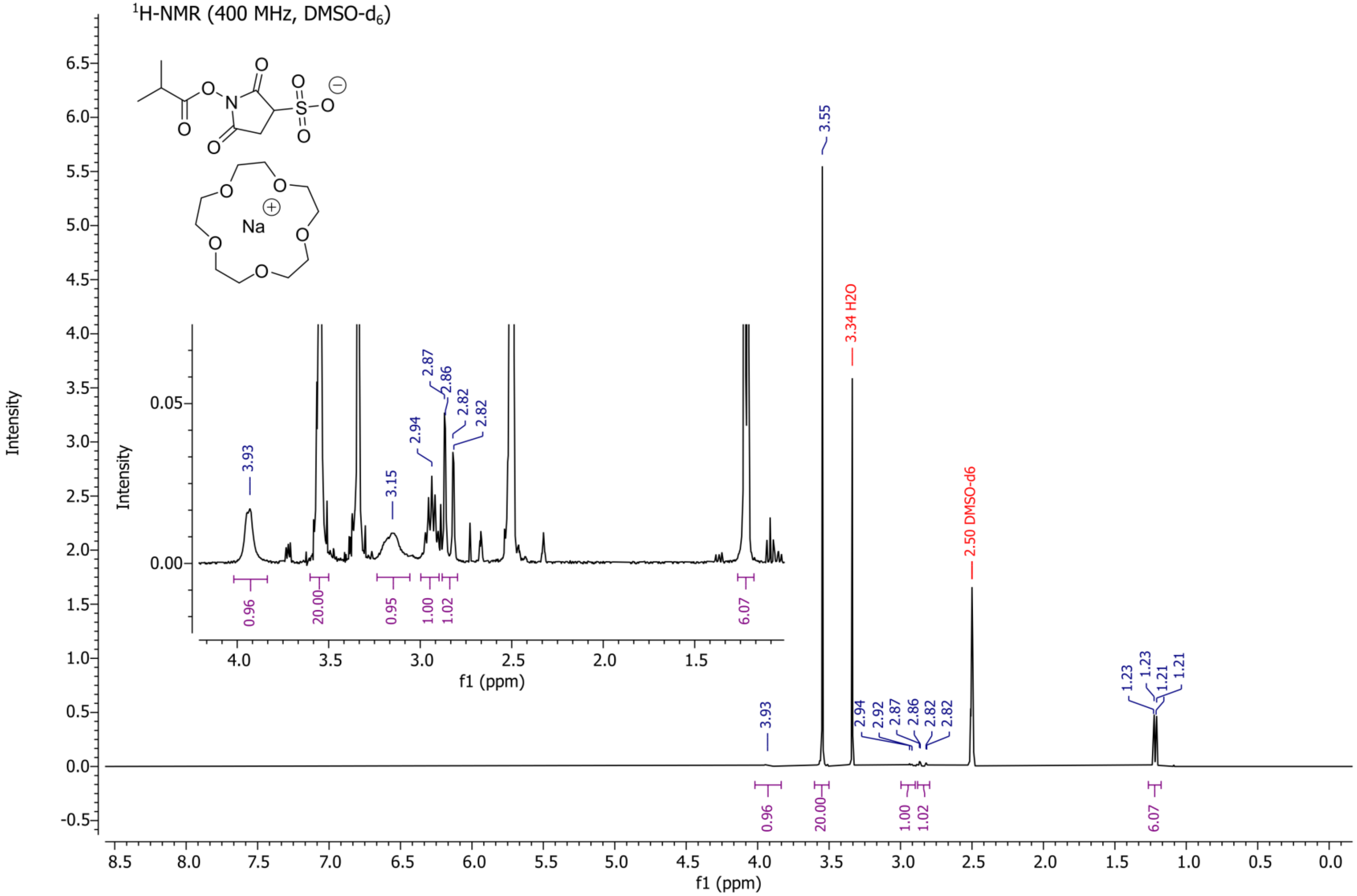

Figure S 42. ^{1}H-NMR spectrum of **9**.

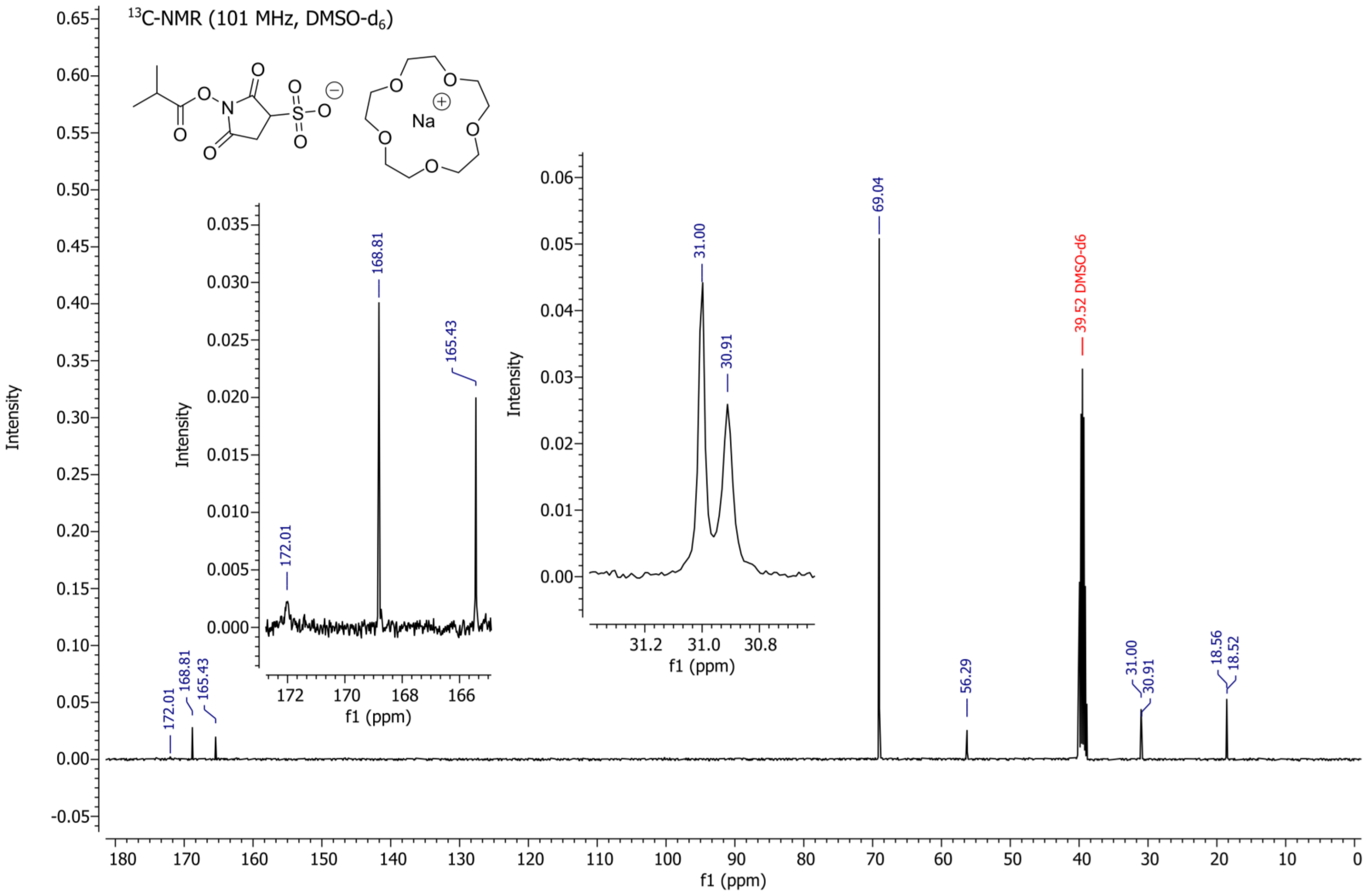

Figure S 43. ^{13}C-NMR spectrum of **9**.

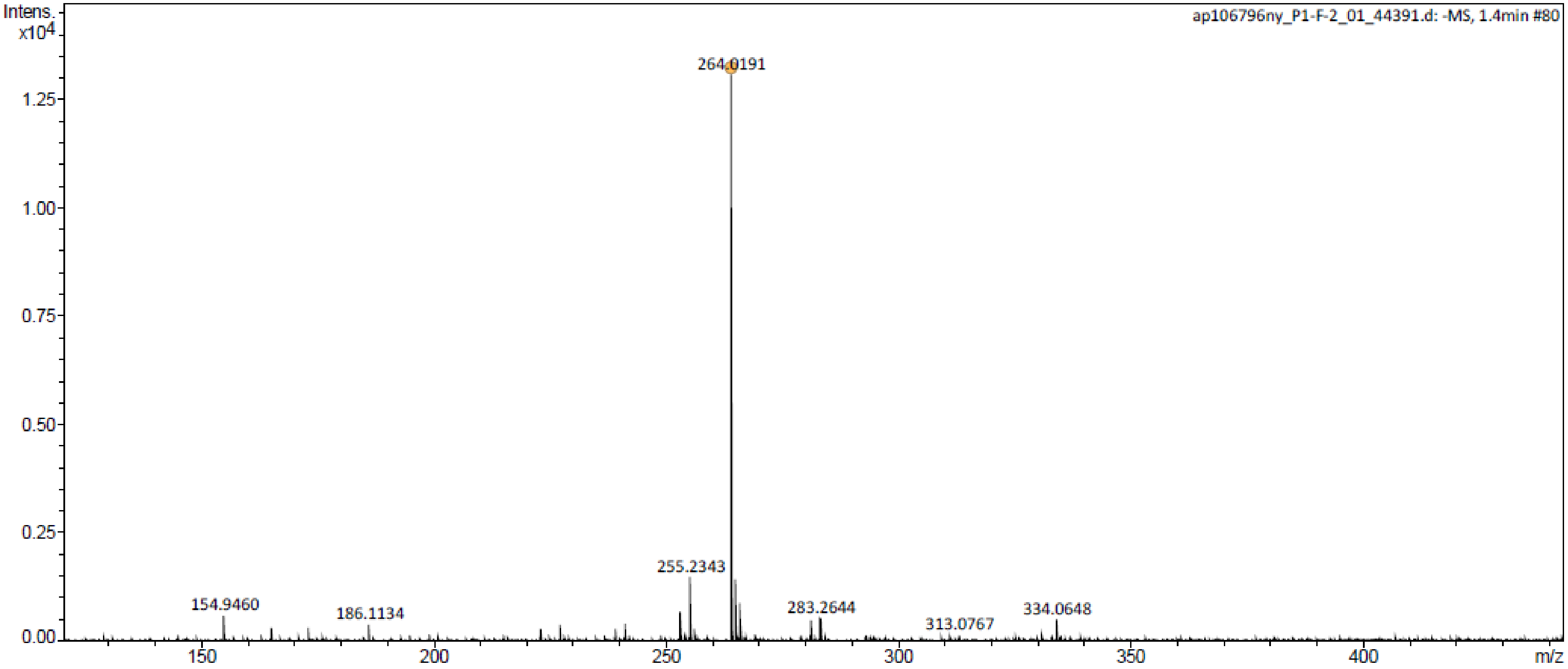

Meas. m/z	#	Ion Formula	m/z	err [ppm]	err [mDa]	mSigma	Mean err [ppm]
264.0191	1	C8H10NO7S	264.0183	-2.8	-0.7	4.8	0.7

Figure S 44. Negative-mode (ESI)HRMS of **9**.

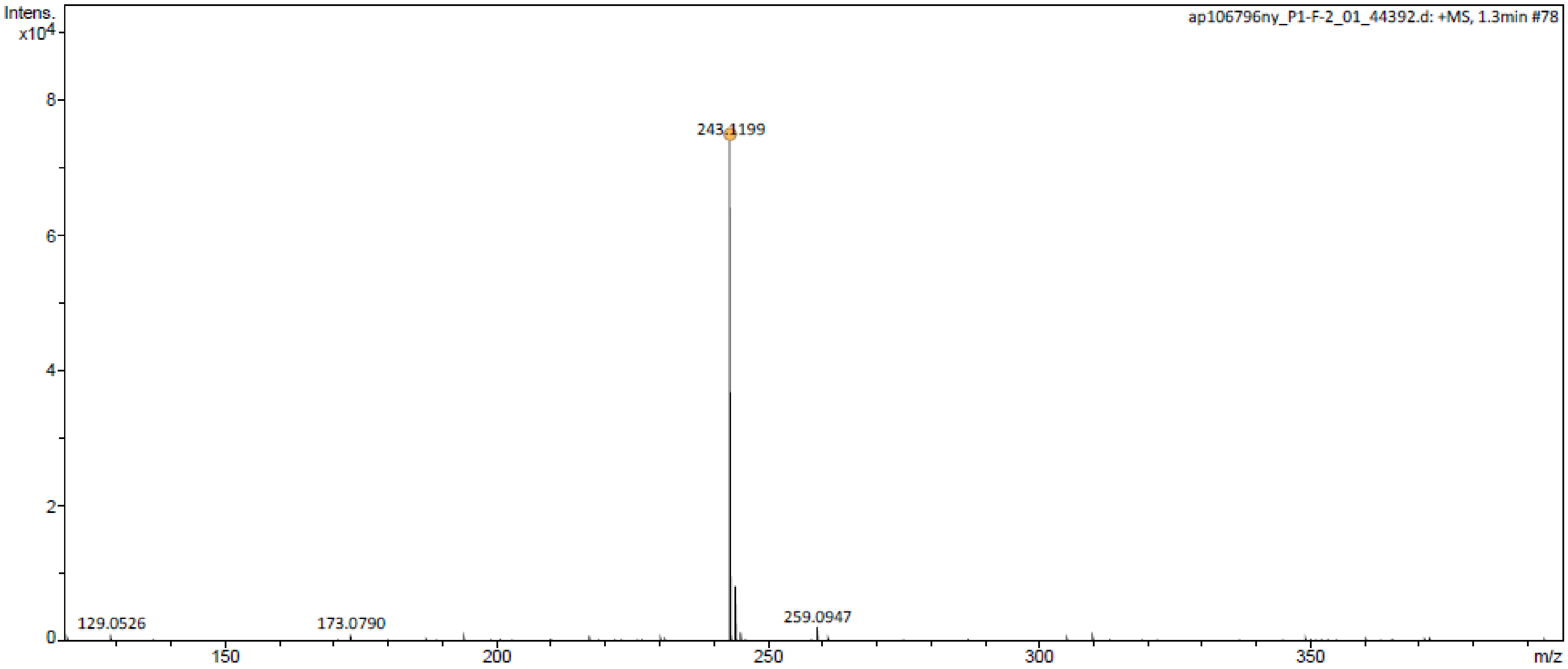

Meas. m/z	#	Ion Formula	m/z	err [ppm]	err [mDa]	mSigma	Mean err [ppm]
243.1199	1	C10H20NaO5	243.1203	1.6	0.4	1.0	3.1

Figure S 45. Positive-mode (ESI)HRMS of **9**.

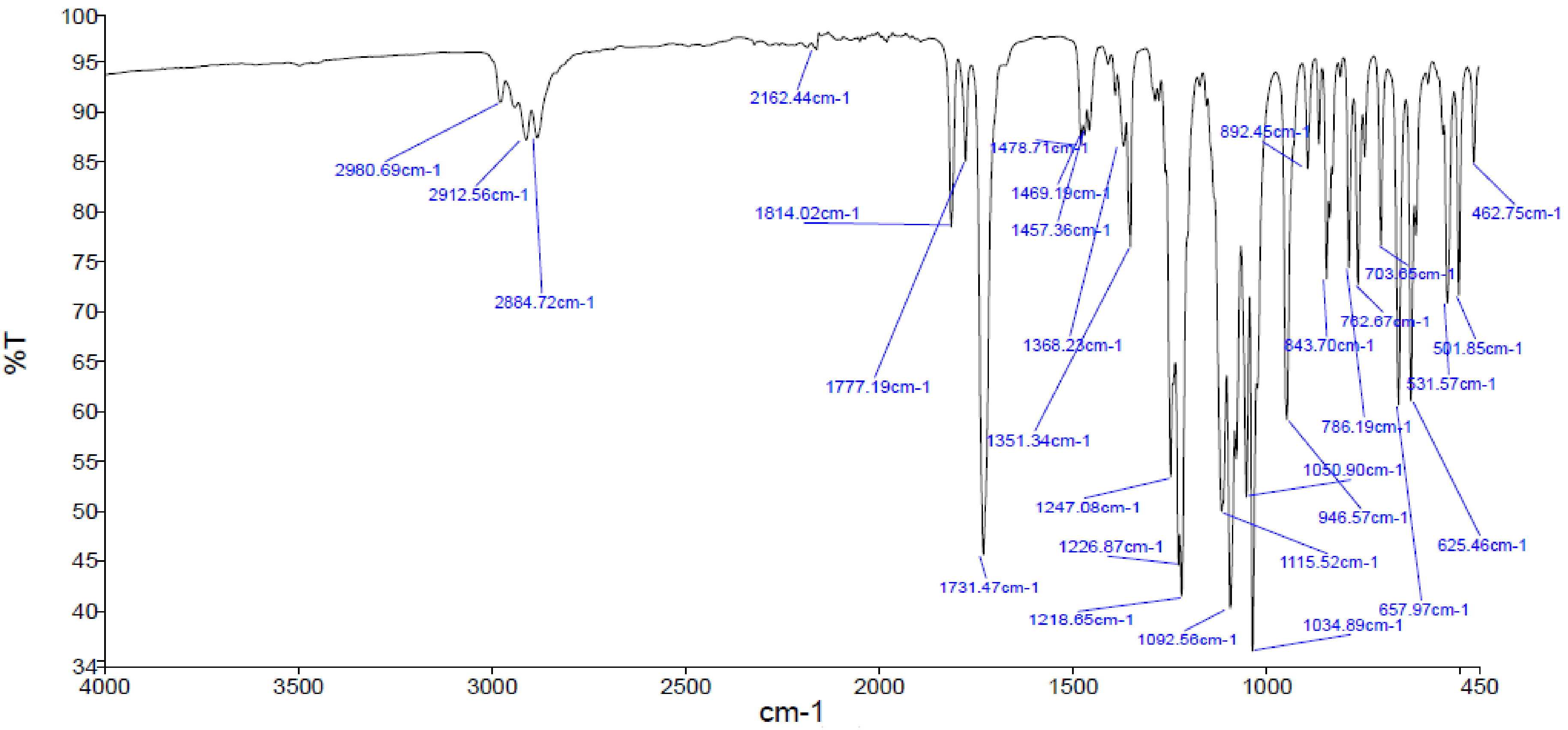

Figure S 46. FT-IR (ATR) spectrum of **9.**

***N*α-Acetyl-*N*ε-isobutyryl-L-lysine S3**

To *N*α-Acetyl-L-lysine (188 mg, 1.0 mmol) in DMSO (15 mL) was added isobutyric anhydride (166 µL, 1.0 mmol). The resultant mixture was heated until full dissolution of all reactants was observed. The resultant solution was then stirred overnight at 100°C.

The reaction solution was then diluted to below 5% v/v DMSO with water. The resultant mixture was then flash-frozen using liquid nitrogen and lyophilized. The residue yielded was then purified via silica column chromatography (DCM → DCM + 10% MeOH), to yield **S3** as a waxy residue that solidified into an off-white solid (0.132 g, 73%).

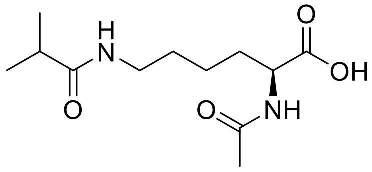

Figure S 47. The structure of **S3**.

^{1}H-NMR (400 MHz, Methanol-d$_4$): δ_H 4.34 (dd, *J* = 8.93, 4.94 Hz, 1H), 3.16 (t, *J* = 6.90 Hz, 2H), 2.41 (spet, *J* = 6.90 Hz, 1H), 1.99 (s, 3H), 1.91-1.80 (m, 1H), 1.75-1.63 (m, 1H), 1.60-1.46 (m, 2H), 1.46-1.32 (m, 2H), 1.10 (d, *J* = 6.90 Hz, 6H).

^{13}C-NMR (101 MHz, Methanol-d$_4$): δ_c 180.1, 175.5, 173.3, 53.7, 39.9, 36.3, 32.3, 30.0, 24.2, 22.3, 19.9.

FT-IR (ATR) (υmax/cm^{-1}): 3357 (N-H stretch), 3299 (N-H stretch), 2969 (C-H stretch, alkyl), 2933 (C-H stretch, alkyl), 2862 (C-H stretch, alkyl), 1720 (C=O stretch), 1637 (C=O stretch), 1583 (N-H bend), 1552 (N-H bend).

(ESI)HRMS: Found 281.1469, $C_{12}H_{22}N_2NaO_4^+$ requires 281.1472.

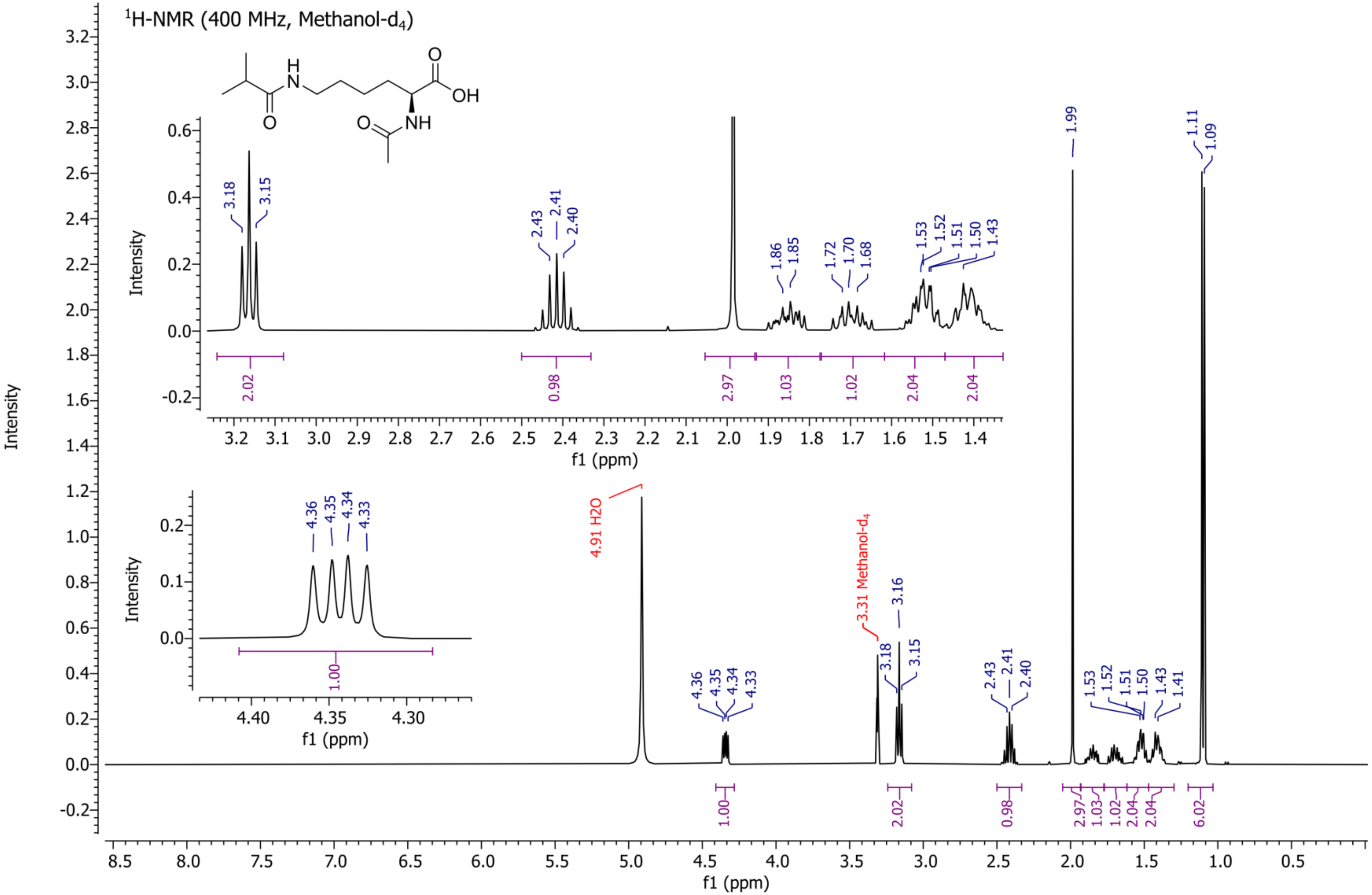

Figure S 48. ^{1}H-NMR spectrum of **S3**.

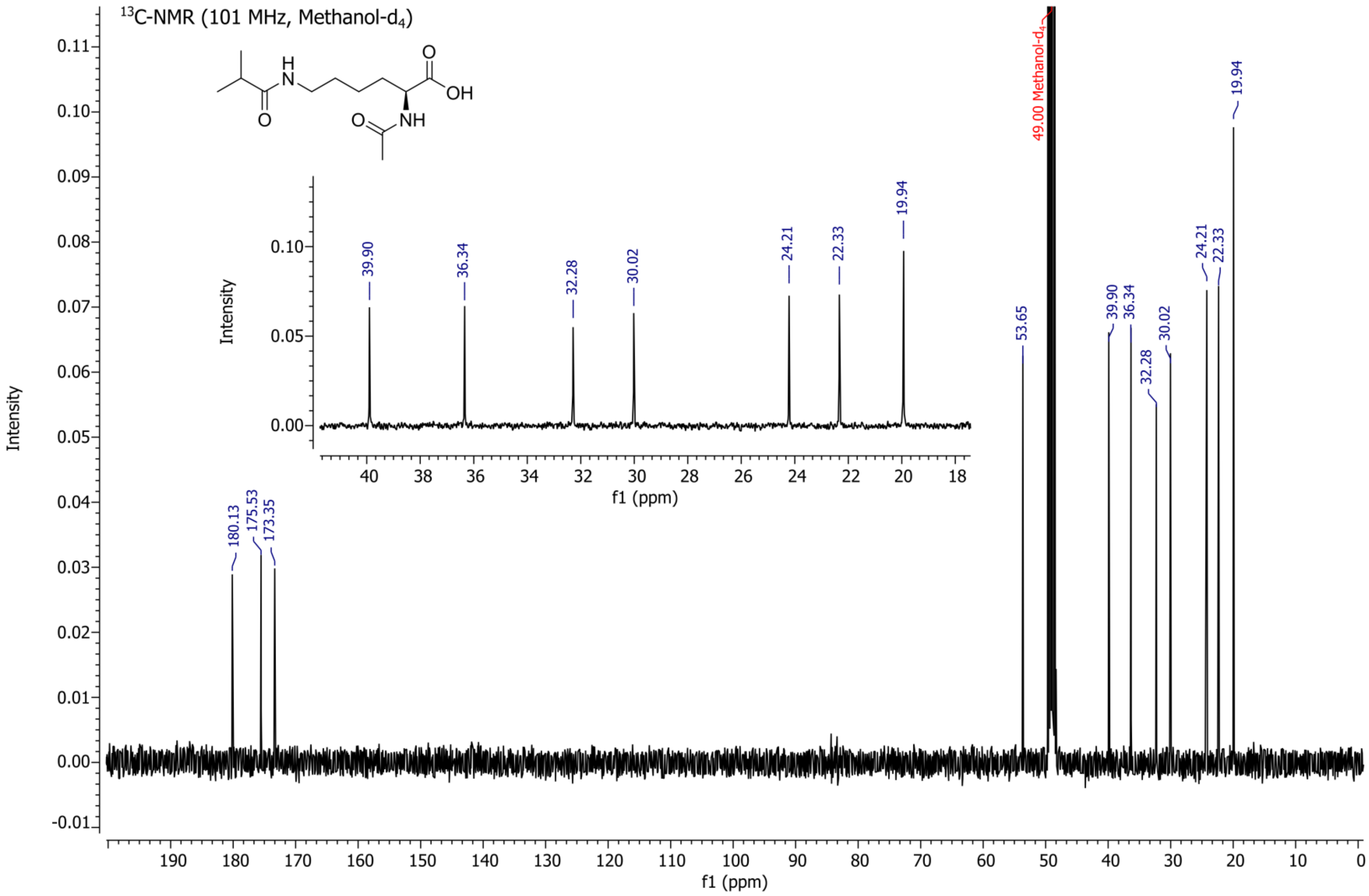

Figure S 49. ^{13}C-NMR spectrum of **S3**.

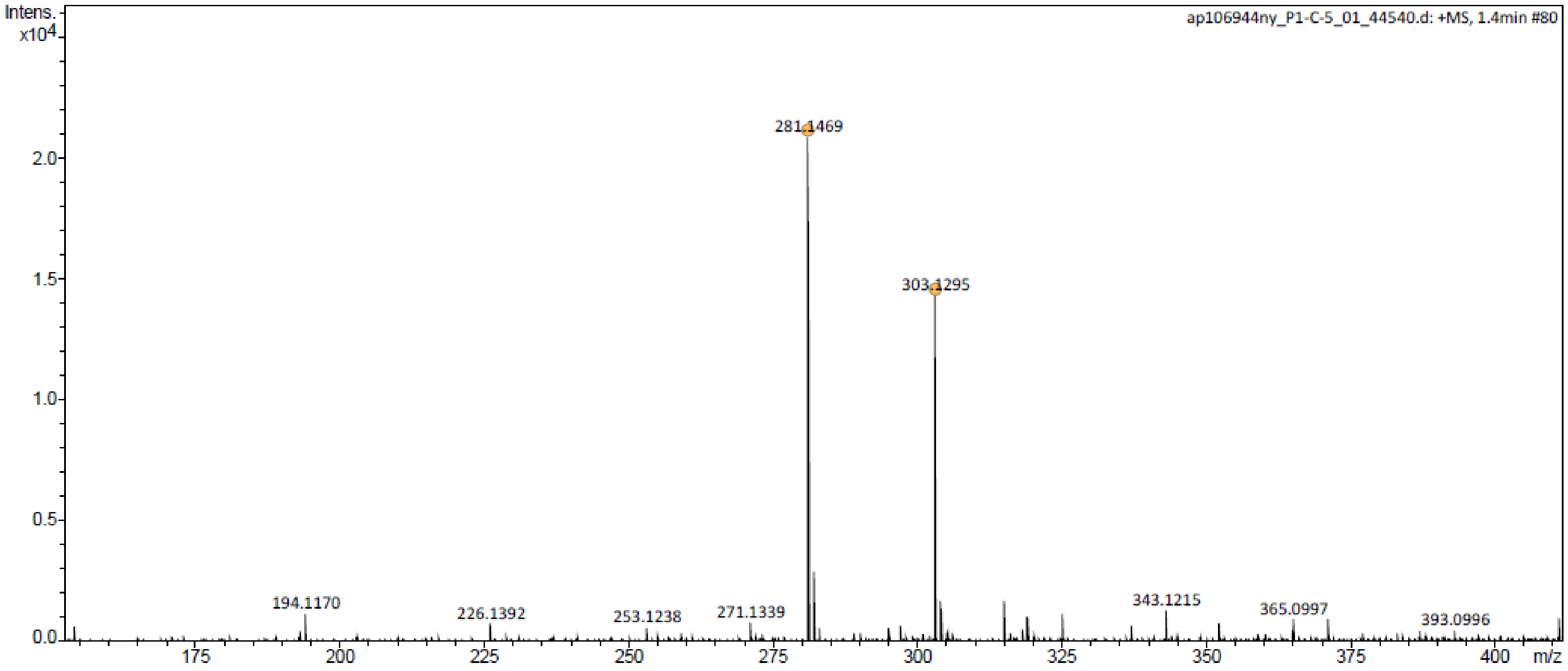

Meas. m/z	#	Ion Formula	m/z	err [ppm]	err [mDa]	mSigma	Mean err [ppm]
281.1469	1	C12H22N2NaO4	281.1472	1.2	0.3	5.1	-1.0
303.1295	1	C12H21N2Na2O4	303.1291	-1.1	-0.3	13.7	1.7

Figure S 50. (ESI)HRMS of **S3**.

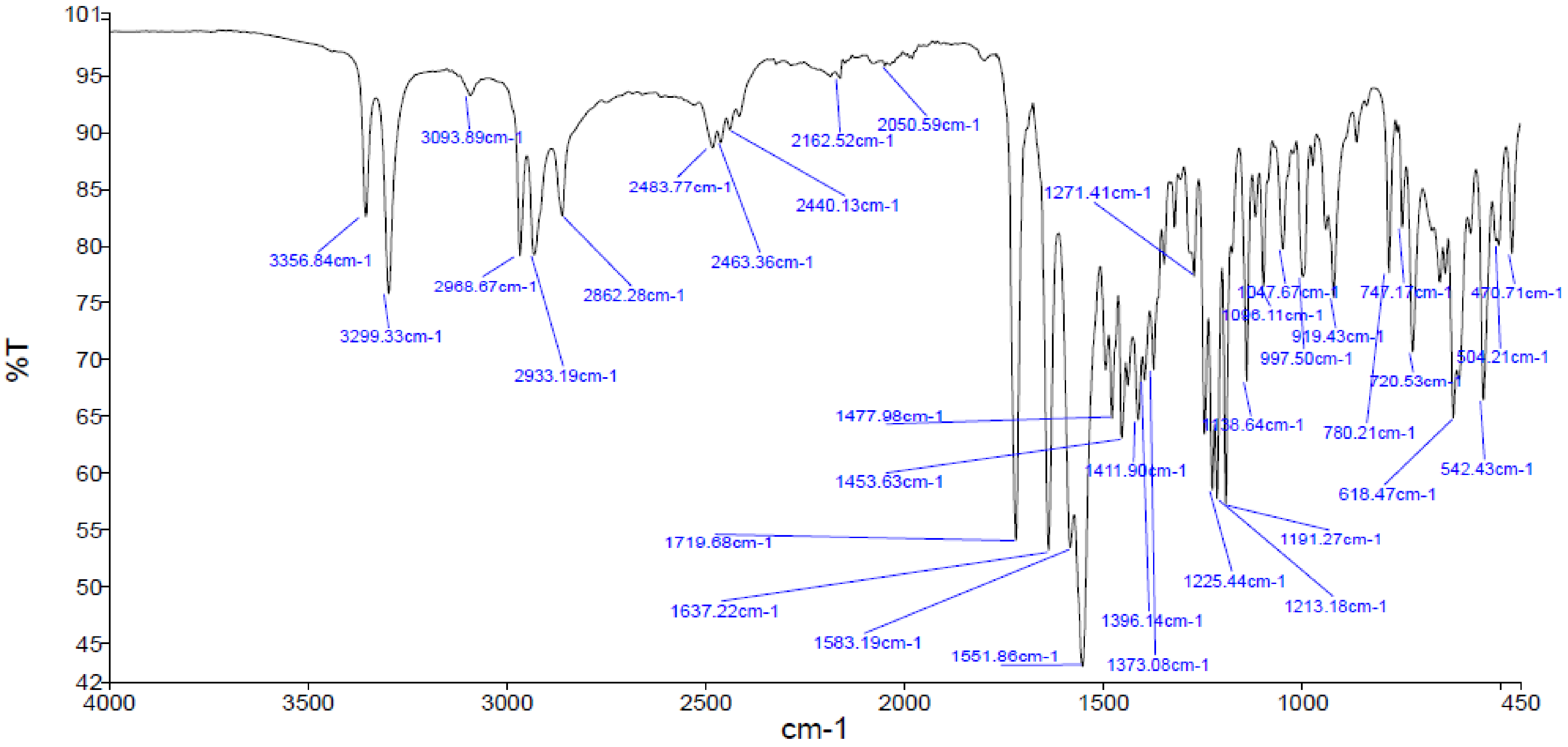

Figure S 51. FT-IR (ATR) spectrum of **S3**.

(R)-2-acetamido-3-(isobutyrylthio)propanoic acid S4

To *N*-Acetyl-L-cysteine (163 mg, 1.0 mmol) in DMF (10 mL) was added triethylamine (140 µL, 1.0 mmol) and isobutyric anhydride (166 µL, 1.0 mmol). The resultant mixture was then stirred overnight at overnight at 100°C.

The reaction solution was then concentrated *in vacuo* to remove the majority of the DMF, and the resultant residue was resuspended in EtOAc (40 mL) and acetic acid (2 mL) was added. The mixture was then transferred to a separating funnel and washed with eater (3 × 20 mL) and brine (20 mL). The organic layer was then dried over $MgSO_4$ and concentrated *in vacuo*. This crude residue was then then purified via silica column chromatography (DCM → DCM + 10% MeOH), to yield **S4** as a waxy residue that solidified into a white solid (0.124 g, 53%).

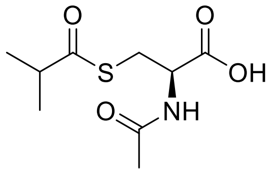

Figure S 52. The structure of **S4**.

^{1}H-NMR (400 MHz, Methanol-d$_4$): δ_H 4.58 (dd, *J* = 8.20, 4.68 Hz, 1H), 3.49 (dd, *J* = 13.8, 4.68 Hz, 1H), 3.13 (dd, *J* = 13.8, 8.20 Hz, 1H), 2.76 (spet, *J* = 6.90 Hz, 1H), 1.96 (s, 3H), 1.17 (d, *J* = 6.90 Hz, 6H).

^{13}C-NMR (101 MHz, Methanol-d$_4$): δ_C 204.5, 173.2, 173.1, 53.3, 44.2, 30.8, 22.3, 19.7, 19.6.

FT-IR (ATR) (υmax/cm^{-1}): 3332 (N-H stretch), 2973 (C-H stretch, alkyl), 2876 (C-H stretch, alkyl), 1703 (C=O stretch), 1677 (C=O stretch), 1610 (C=O stretch), 1560 (N-H bend).

(ESI)HRMS: Found 256.0623, $C_9H_{15}NNaO_4S^+$ requires 256.0614.

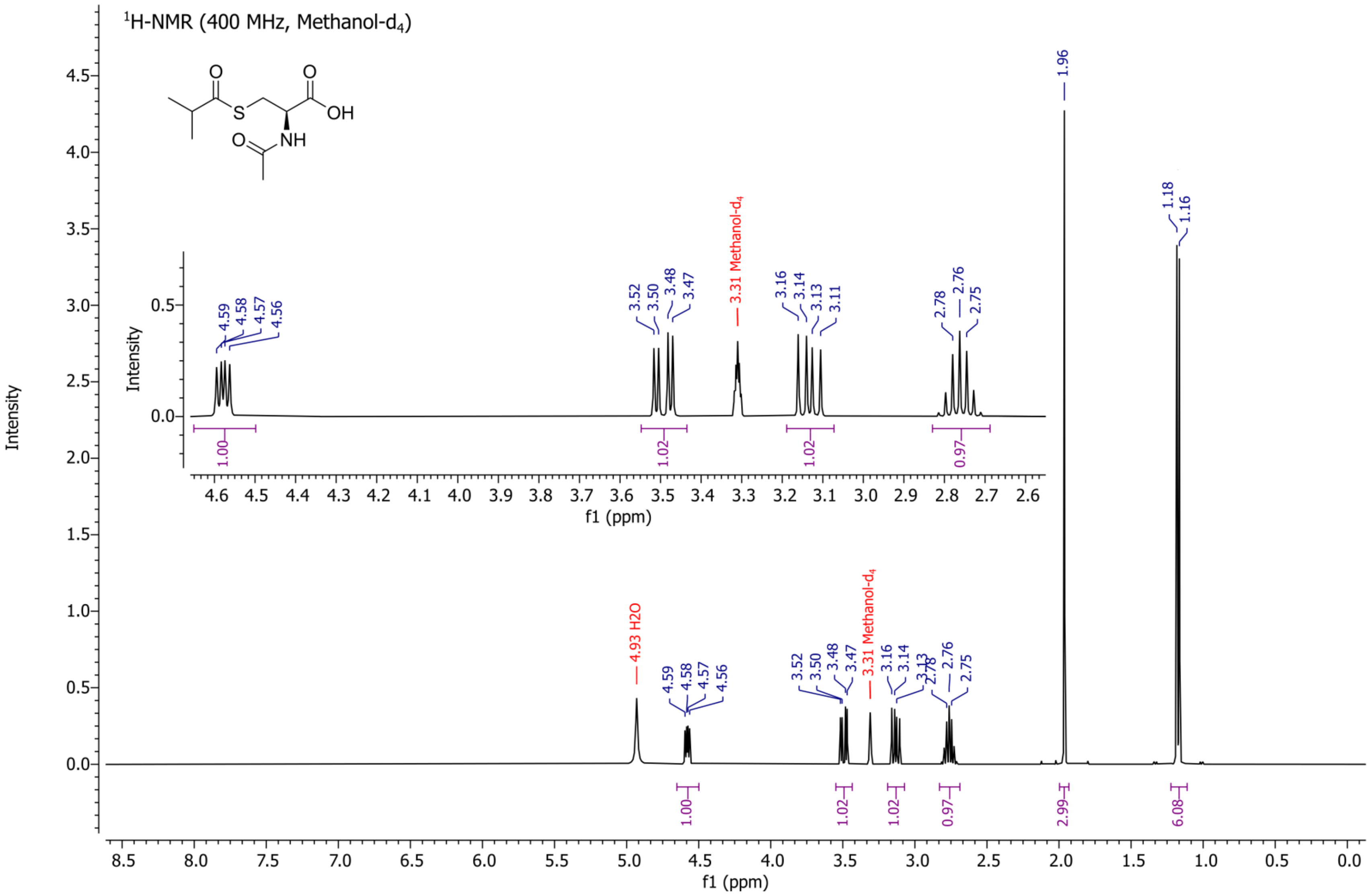

Figure S 53. ^{1}H-NMR spectrum of **S4**.

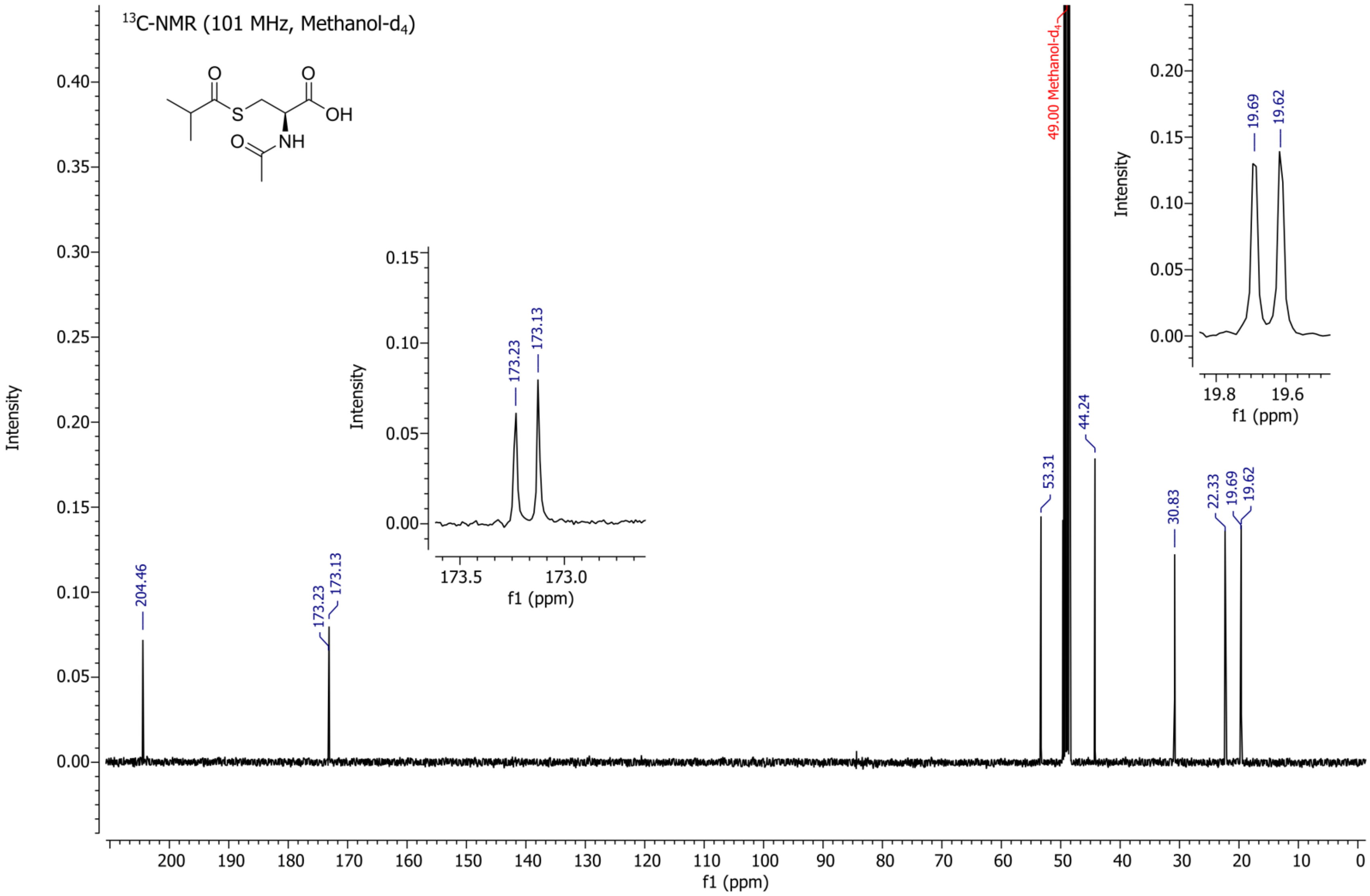

Figure S 54. ^{13}C-NMR spectrum of **S4**.

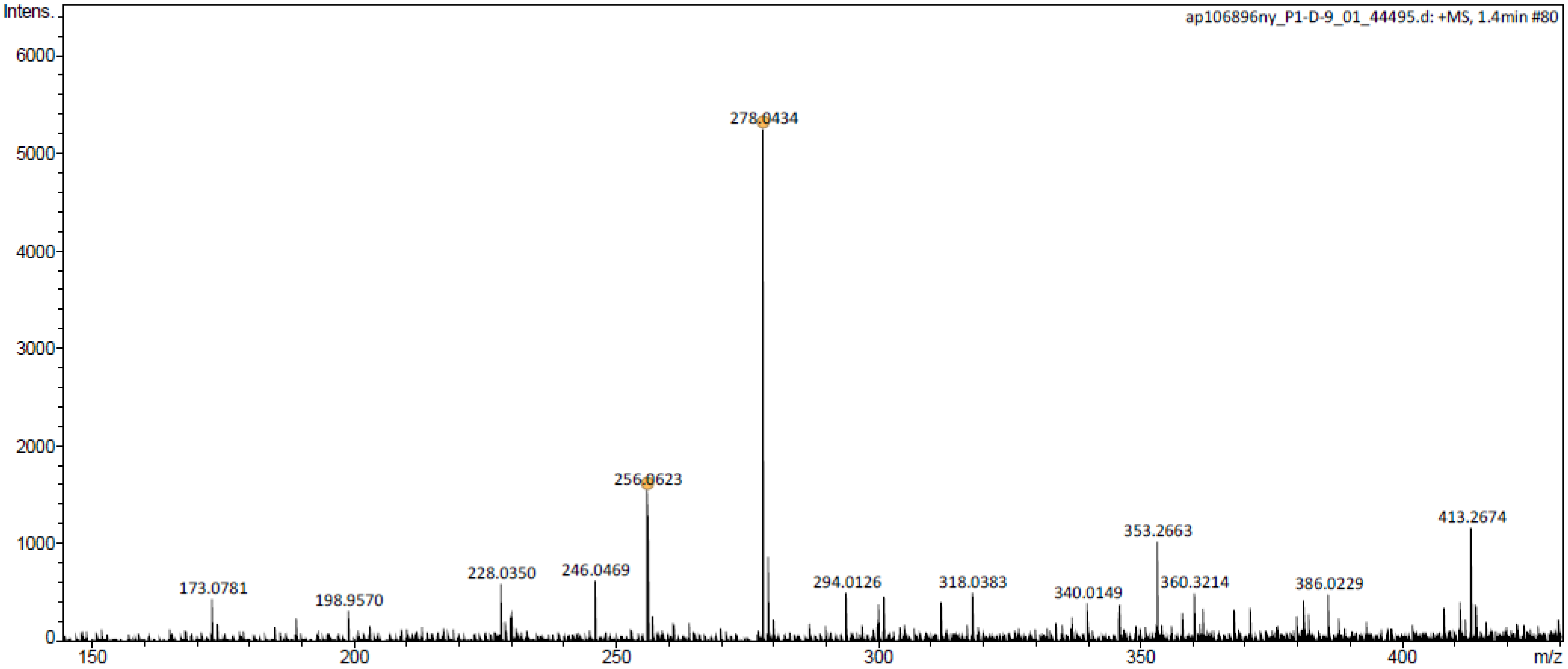

Meas. m/z	#	Ion Formula	m/z	err [ppm]	err [mDa]	mSigma	Mean err [ppm]
256.0623	1	C9H15NNaO4S	256.0614	-3.4	-0.9	41.3	-2.4
278.0434	1	C9H14NNa2O4S	278.0433	-0.3	-0.1	28.3	-1.1

Figure S 55. (ESI)HRMS of **S4**.

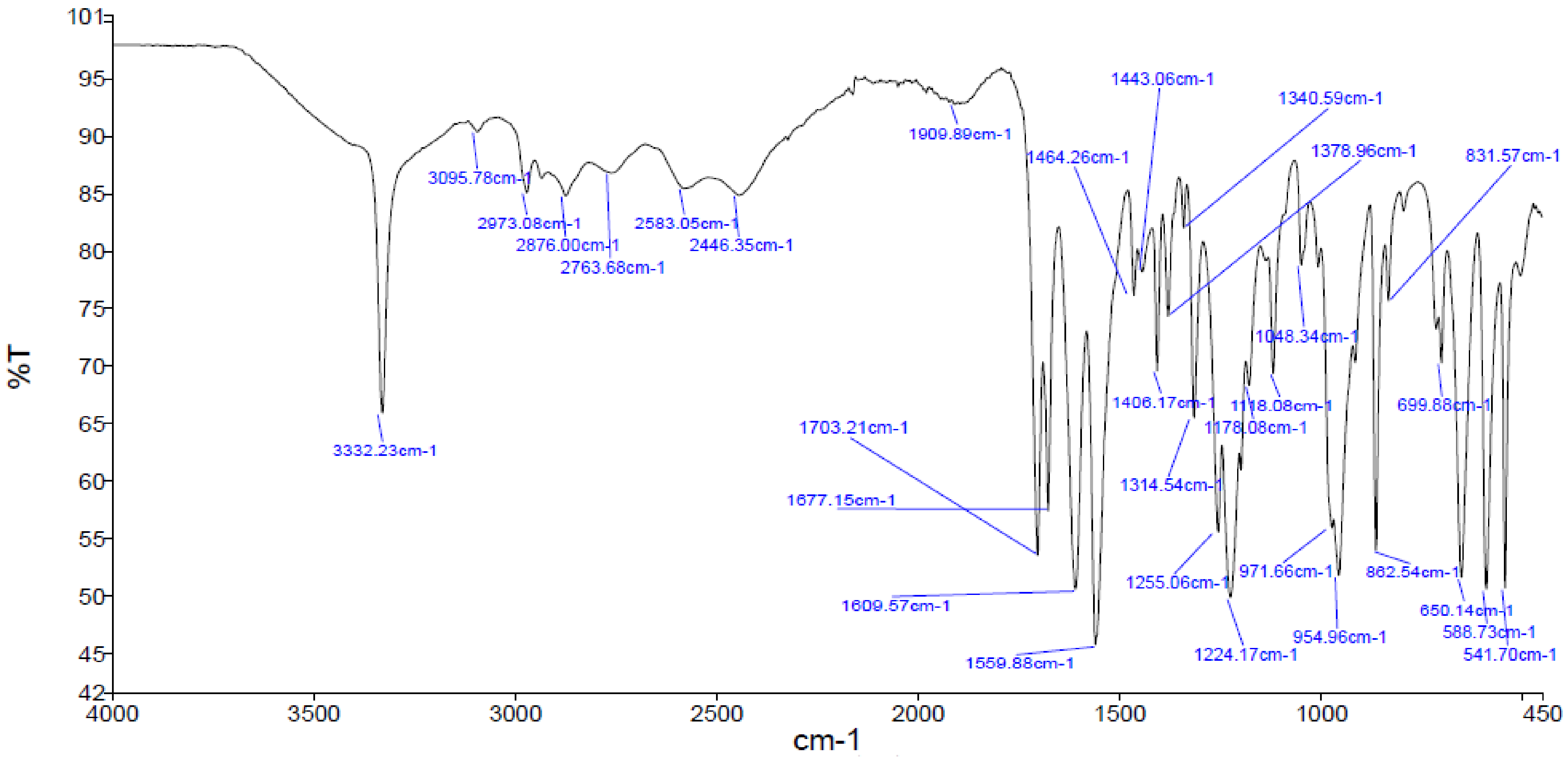

Figure S 56. FT-IR (ATR) spectrum of **S4**.

2,5-dioxo-1-((4-(pyren-1-yl)butanoyl)oxy)pyrrolidine-3-sulfonate sodium salt 15-crown-5 complex 11

1-Pyrenebutyric acid **10** (50.0 mg, 0.17 mmol) and **3** (87.5 mg, 0.20 mmol) were weighted into a sample vial and were dissolved in anhydrous DMF (2 mL). DCC (35.8 mg, 0.17 mmol) was then added, and the vial was sealed shut to prevent excessive exposure to moisture. The resultant reaction mixture was stirred overnight at room temperature in darkness. Insoluble material was then removed by filtration (using Pasteur pipette with a cotton wool plug) and the eluate was dry loaded onto silica and the product purified via flash column chromatography (hexane → DCM → 20% MeOH), yielding **11** as a light brown solid (62 mg, 51%).

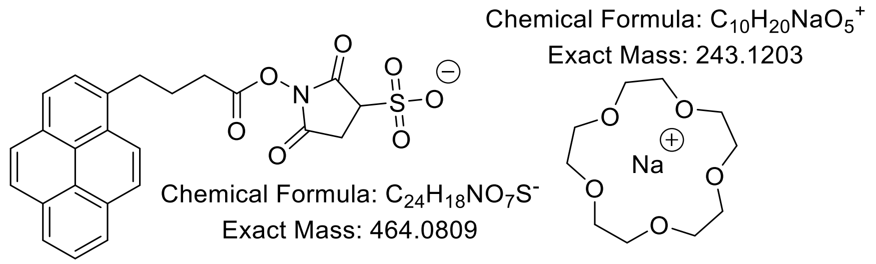

Figure S 57. The structure of **11**.

^{1}H-NMR (400 MHz, DMF-d$_7$): δ_H 8.52 (d, *J* = 9.30 Hz, 1H), 8.34-8.25 (m, 4H), 8.20-8.16 (m, 2H), 8.12 – 8.06 (m, 1H), 8.05 (d, *J* = 8.20 Hz, 1H), 4.16 (dd, *J* = 8.50, 2.20 Hz, 1H), 3.62 (s, 20H), 3.56-3.50 (m, 2H), 3.38-3.28 (m, 1H), 3.18 (dd, *J* = 18.10, 2.20 Hz, 1H), 2.97-2.90 (m, 2H), 2.31-2.20 (m, 2H).

^{13}C-NMR (101 MHz, DMF-d$_7$): δ_C 170.3, 167.1, 137.0, 132.4, 132.0, 131.1, 129.6, 128.7, 128.6, 127.8, 127.2, 126.2, 126.1, 126.0, 125.8, 125.7, 124.5, 70.0, 57.9, 32.8, 32.3, 28.1.

FT-IR (ATR) (υmax/cm^{-1}): 2874 (C-H stretch, alkyl), 1813 (C-H bend, aromatic), 1784 (C-H bend, aromatic), 1735 (C=O stretching), 1228 (C-O stretch, ester), 849 (C-H bend, aromatic).

(ESI)HRMS: Found 464.0821, $C_{24}H_{18}NO_7S^-$ requires 464.0809. Found 243.1199, $C_{10}H_{20}NaO_5^+$ requires 243.1203.

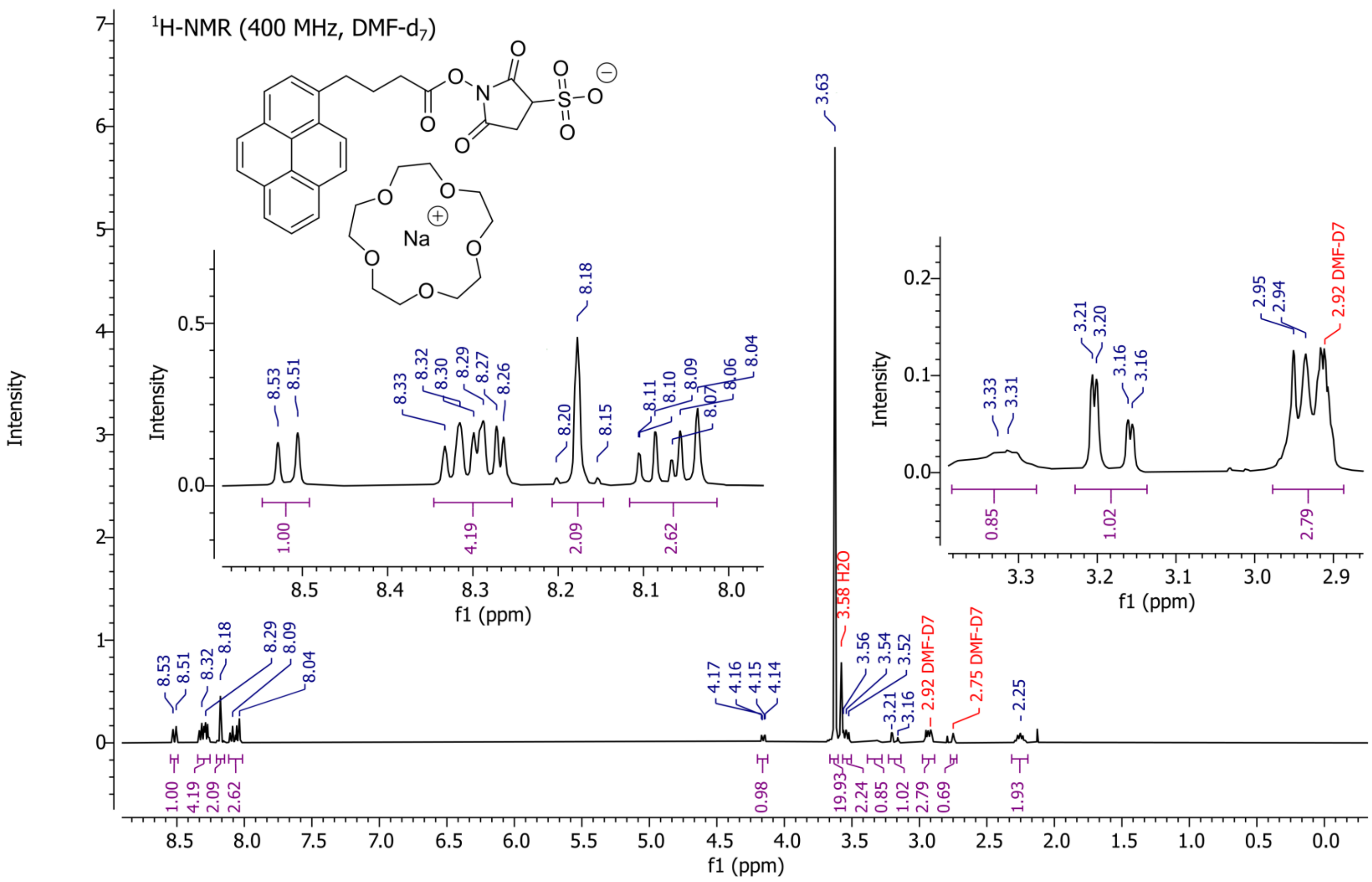

Figure S 58. ^{1}H-NMR spectrum of **11**.

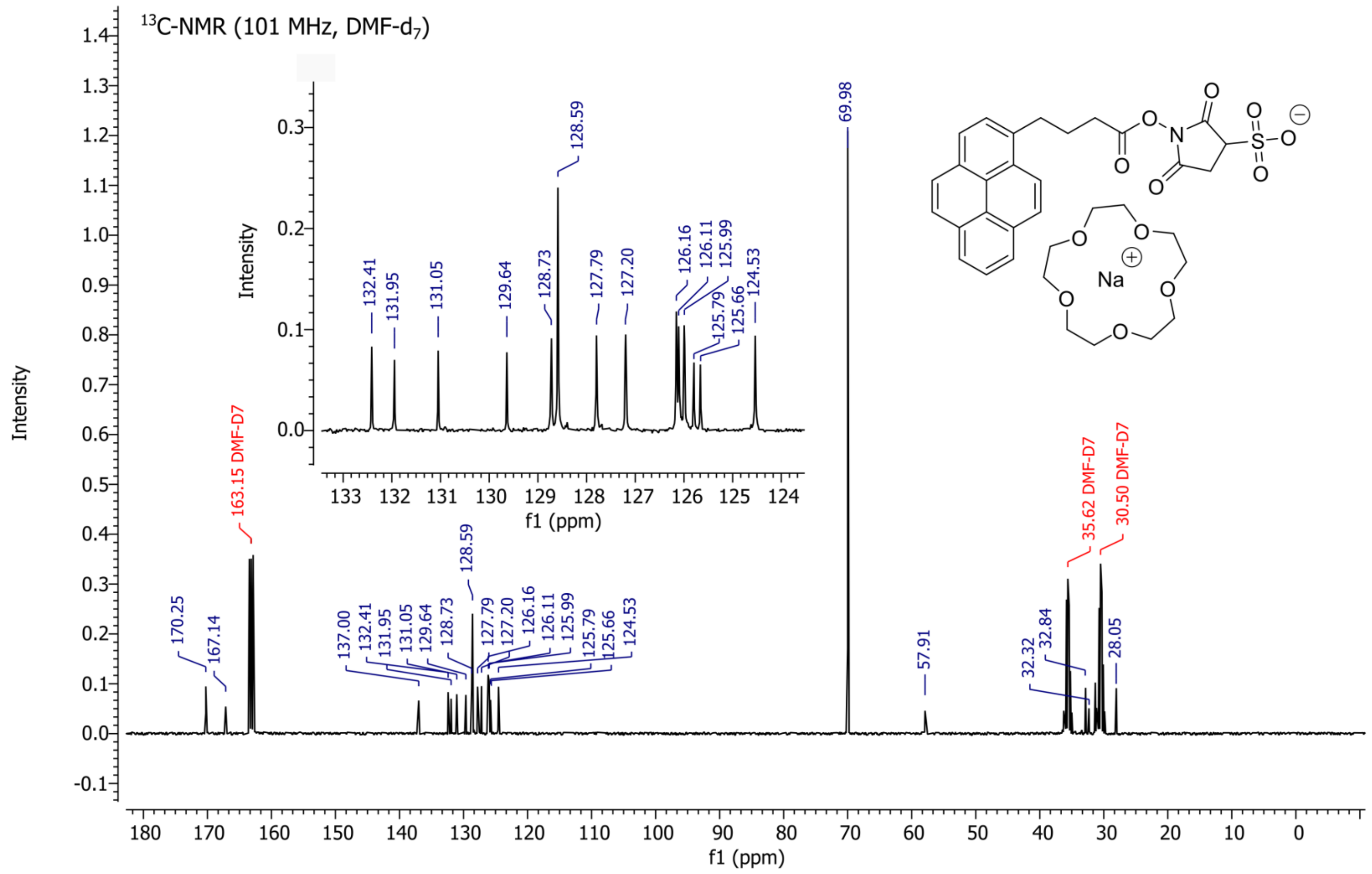

Figure S 59. ^{13}C-NMR spectrum of **11**.

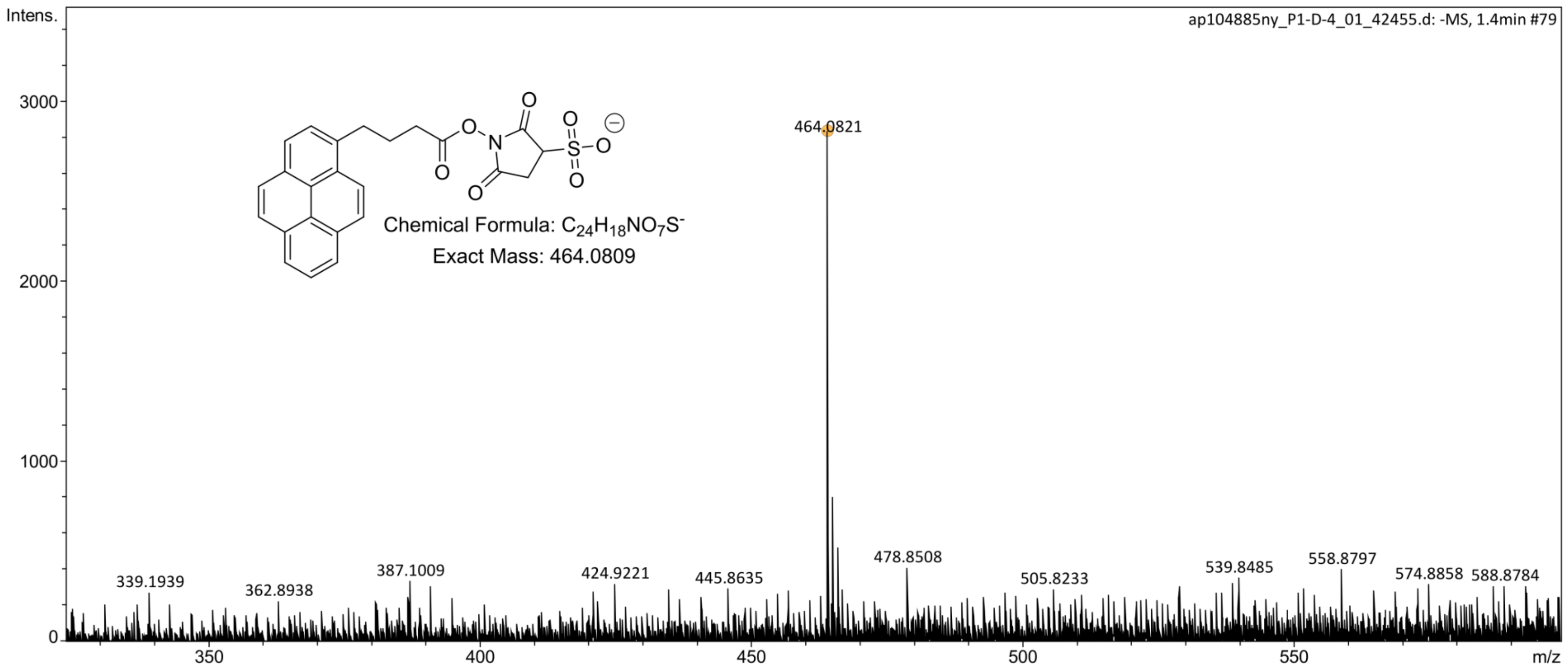

Meas. m/z	#	Ion Formula	m/z	err [ppm]	err [mDa]	mSigma	Mean err [ppm]
464.0821	1	C24H18NO7S	464.0809	-2.4	-1.1	49.1	-3.8

Figure S 60. Negative-mode (ESI)HRMS of **11**.

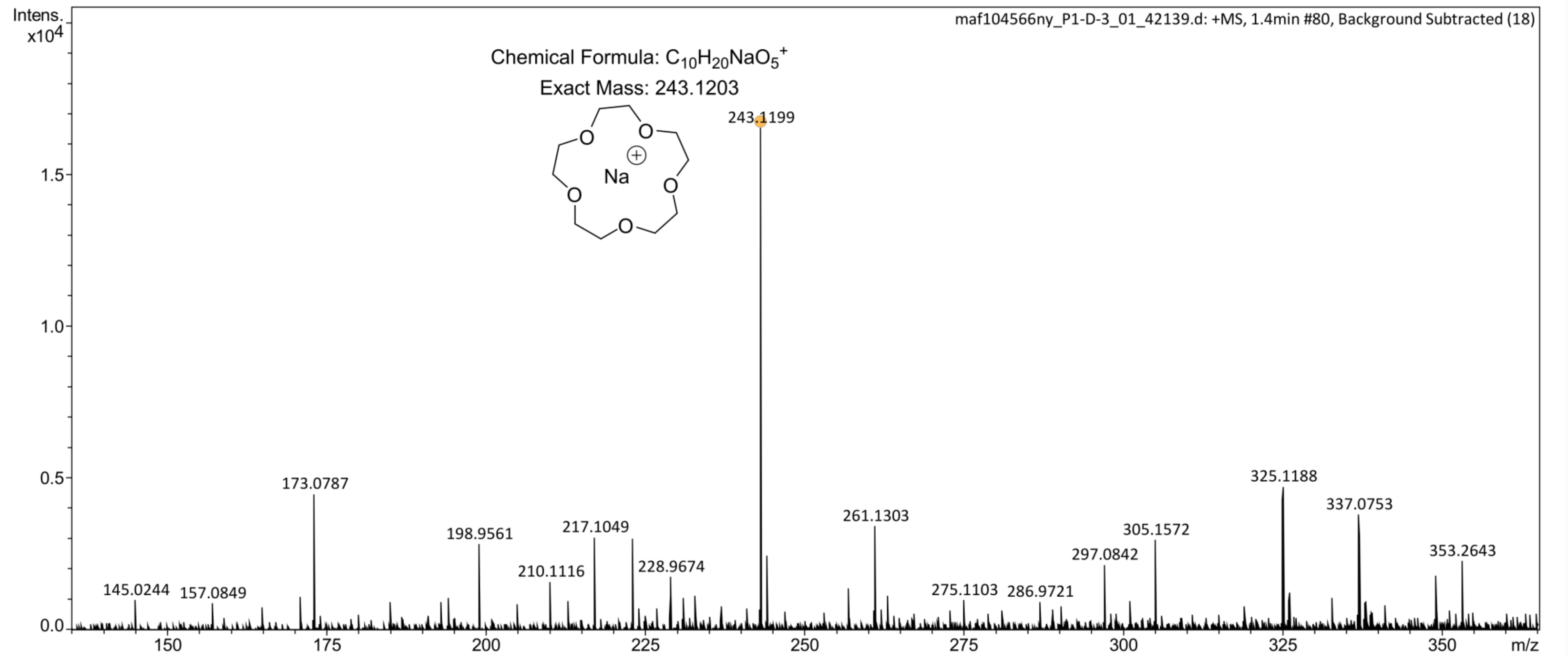

Meas. m/z	#	Ion Formula	m/z	err [ppm]	err [mDa]	mSigma	Mean err [ppm]
243.1199	1	C10H20NaO5	243.1203	1.7	0.4	23.3	0.6

Figure S 61. Positive-mode (ESI)HRMS of **11**.

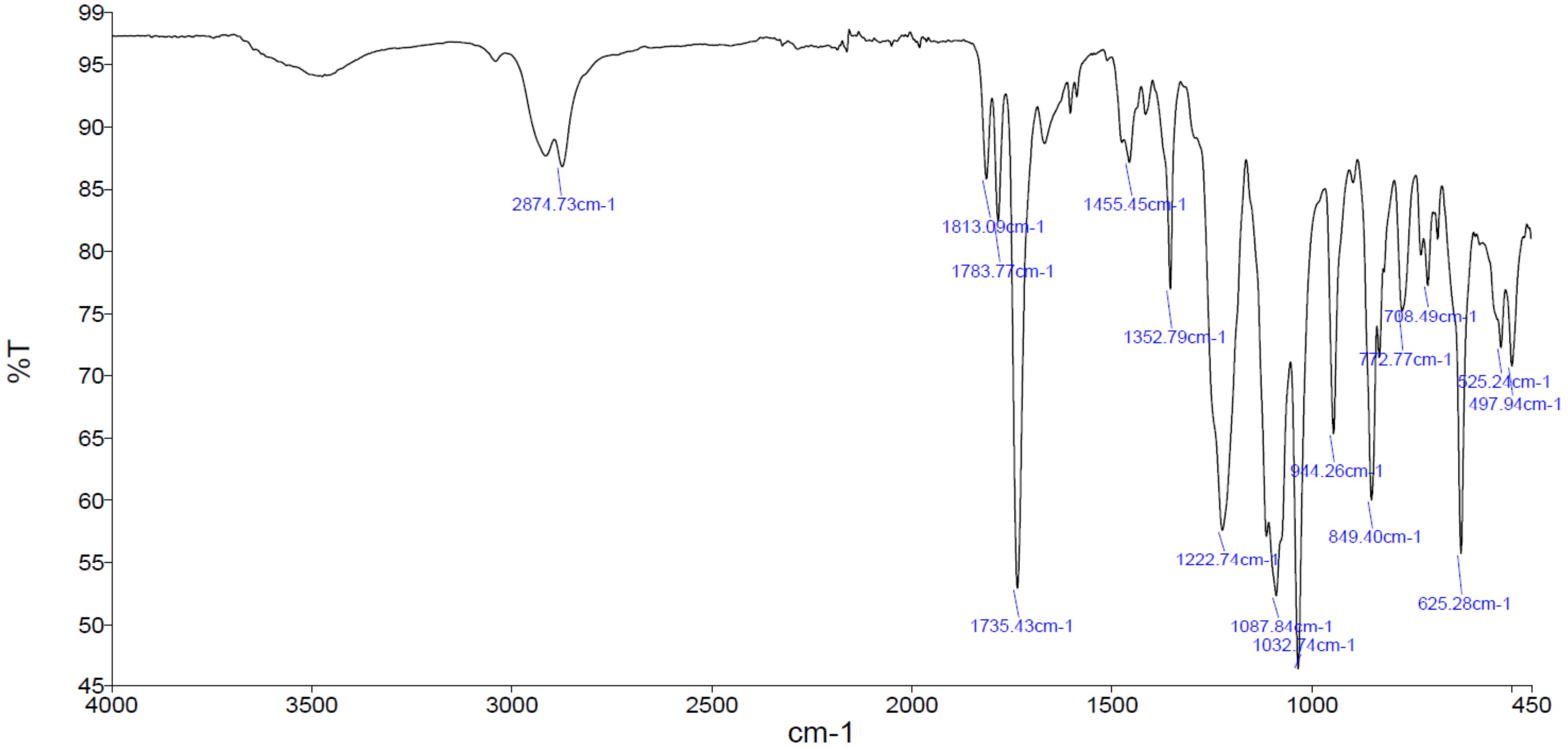

Figure S 62. FT-IR (ATR) spectrum of **11**.

Protein preparation, molecular weight calculations and SDS page gel analysis

Protein preparation and concentration quantification

DsbA[4] and CjX183-D R51K[5] were prepared according to the procedures reported in our previously published works. Prior to their use, the concentrations of samples of DsbA and CjX183-D R51K in bioconjugation reactions were determined using the extinction coefficients tabulated below.

Table S1. Molar extinction coefficients for DsbA and CjX183-D R51K proteins.

Protein	Extinction coefficient
DsbA	ε_{280} = 16700 mol^{-1} dm^{3} cm^{-1}
CjX183-D R51K, oxidised form (i.e. Fe^{3+} in heme)	ε_{410} = 106000 mol^{-1} dm^{3} cm^{-1}

Analysis of proteins via Sodium dodecyl sulfate polyacrylamide gel electrophoresis (SDS-PAGE)

SDS-PAGE gel analysis of DsbA was performed using the procedure detailed in our previously published work.[4]

Calculation of protein theoretical masses:

CjX183-D R51K

Sequence:

GYLVGDATRGANLWNTQTCVACHGVDGERNASGTPALTPLNPNRDLYRHSKDTQDRALRDFISMWMPQGNEG SCTGQCAADIEAFIRTWHHHHHH

- Factoring in the structural disulfide between C78 and C74, the chemical formula of the apo protein can be calculated to be $C_{452}H_{682}N_{146}O_{140}S_6$.
- The two remaining cysteine residues ligate to heme C:

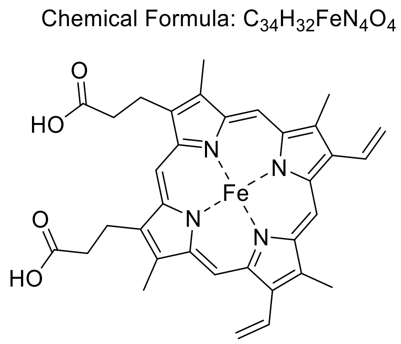

- $C_{452}H_{682}N_{146}O_{140}S_6 + C_{34}H_{32}FeN_4O_4 = C_{486}H_{714}FeN_{150}O_{144}S_6$.
- The relative molecular mass of $C_{486}H_{714}FeN_{150}O_{144}S_6$ is 11210.17 Da.

DsbA mutant

Sequence:

MHHHHHHGKPIPNPLLGLDSTENLYFQGIDPFTSRDDKKDGVAGPGDAVRVTSSKLVTQPGTSNPKAVVSFYEDF LCPACGIFERGFGPTVSKLVDIGAVAADYTMVAILDSASNQHYSSRAAAAAYCVADESIEAFRRFHAALFSKDIQPAE LCTPSRDNARLIELAREAGVVGKVPDCINSGKYIEKVDGLAAAVNVHATPTVRVNGTEYEWSTPAALVAKIKEIVGD VPGIDSAAATATS

- Factoring in the structural disulfide between the highlighted cysteine residues, the chemical formula of the protein can be calculated to be $C_{1138}H_{1780}N_{316}O_{353}S_7$.
- The relative molecular mass of $C_{1138}H_{1780}N_{316}O_{353}S_7$ is 25760.60 Da.

Bioconjugation of proteins

Reaction of CjX183-D R51K with esters 5-7/7'

To 140 µL of a 55 µM solution of CjX183-D R51K in pH 8.3 buffer (50 mM sodium borate) was added 20 equiv. of either ester **5**, **6**, **7** or **7'** via the delivery of 14 µL of a 11 mM stock of the relevant ester in DMF. The resultant solutions were then incubated at room temperature in the dark for 1 hour whereupon the labelled CjX183-D R51K samples were buffer exchanged into HPLC-grade water using PD SpinTrap^{TM} G-25 columns. The samples were then immediately analysed via MS.

Note that the 11 mM solution of **7'** was prepared via the delivery of one equiv. of 15-crown-5 (dissolved in DMF) to a concentrated DMF stock solution of **6**, such that the final concentration of **7'** was 11 mM. The 11 mM solution of **6** was prepared from the same concentrated stock solution of **6**, thus ensuring a fair comparison between the performances of **6** and **7'**.

Reaction of DsbA with 11

To 180 µL of a 49 µM solution of DsbA in pH 8.3 buffer (50 mM sodium borate) was added either 1, 3, 6 or 12 equiv. of **11**, via the delivery of 12 µL of DMSO stocks of **11** of appropriate concentration (i.e. 740 µM, 2.22 mM, 4.44 mM and 8.88 mM). The resultant solutions were then incubated at room temperature in the dark for 1 hour before the labelled DsbA samples were buffer exchanged into HPLC-grade water containing 10 mM DTT using PD SpinTrap^{TM} G-25 columns. The samples were then immediately analysed via MS and SDS PAGE gel.

Note that the DTT was used to prevent intermolecular disulfide bond formation, which would have otherwise complicated MS analysis.

Monitoring the rate of Sulfo-NHS-type ester hydrolysis via UV-vis spectroscopy

The hydrolysis of Sulfo-NHS-type esters

The hydrolysis of Sulfo-NHS esters in aqueous solution can be described by the equation below. NHS-type alcohols are strong absorbers of 268 nm UV irradiation, and thus the rate at which the hydrolysis reaction occurs can be determined by tracking the rate at which the UV-vis absorbance at 268 nm increases. By carefully constructing appropriate equations, it should be possible to determine values for k and half-life for the hydrolysis process, allowing the rate of hydrolysis to be examined at a range of pH values and with a range of buffer salts for both Sulfo esters and C-Sulfo esters.

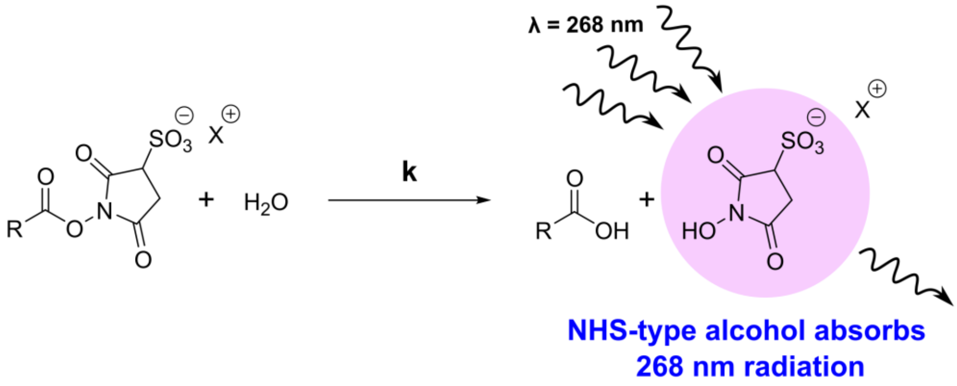

Figure S 63. The hydrolysis of Sulfo-NHS esters.

Derivation of rate equations

Constructing a rate equation for the above process yields **Equation 1.**

$$\frac{d[Sulfo\ NHS\ ester]}{dt} = -k_{second\ order}["H_2O"][Sulfo\ NHS\ ester] \qquad \textbf{Equation 1.}$$

$[Sulfo\ NHS\ ester]$:	The concentration of Sulfo-NHS ester
$-k_{second\ order}$:	The second-order rate constant for the hydrolysis process
$["H_2O"]$:	The concentration of the water-related species responsible for the rate determining step of the hydrolysis reaction.
t:	Time.

It should be noted that the identity of the "H_2O" species responsible for the rate determining step of NHS-ester hydrolysis may not be H_2O itself, and may instead be H_3O^+ or HO^- depending on the pH of the aqueous solution. Regardless, under buffered conditions the concentration of H_2O, H_3O^+ and HO^-

can be considered to be constant throughout the course of the reaction. This allows us to assume a pseudo first order regime, and instead construct the following rate equation (**Equation 2**).

$$\frac{d[Sulfo\ NHS\ ester]}{dt} = -k[Sulfo\ NHS\ ester]$$ **Equation 2.**

k: The pseudo first-order rate constant for the hydrolysis process.

Equation 2 can be rearranged to yield **Equation 3**.

$$\frac{d[Sulfo\ NHS\ ester]}{[Sulfo\ NHS\ ester]} = -k\ dt$$ **Equation 3.**

Integrating this expression over all concentrations of Sulfo-NHS ester and over the timescale of the experiment (**Equation 4**), and by considering $\int \frac{1}{x} = \ln x$, we arrive at **Equation 5**:

$$\int_{[Sulfo\ NHS\ ester]_0}^{[Sulfo\ NHS\ ester]} \frac{d[Sulfo\ NHS\ ester]}{[Sulfo\ NHS\ ester]} = -\int_{t_0}^{t} k\ dt$$ **Equation 4.**

$[Sulfo\ NHS\ ester]_0$: The concentration of Sulfo-NHS ester at the start of the hydrolysis experiment.

$$\ln[Sulfo\ NHS\ ester] - \ln[Sulfo\ NHS\ ester]_0 = -kt$$ **Equation 5.**

$[Sulfo\ NHS\ ester]$ at any given time-point is equal to $[Sulfo\ NHS\ ester]_0 - [NHS\text{-}type\ alcohol]$. Therefore we can construct **Equation 6**:

$$\ln\{[Sulfo\ NHS\ ester]_0 - [NHS\text{-}type\ alcohol]\} - \ln[Sulfo\ NHS\ ester]_0 = -kt$$ **Equation 6.**

$[NHS\text{-}type\ alcohol]$: The concentration of any NHS-type alcohol generated since the start of the hydrolysis experiment.

If the hydrolysis reaction is allowed to reach completion, all the Sulfo-NHS ester will be converted in to NHS-type alcohol. As such, $[Sulfo\ NHS\ ester]_0$ will be equal to the concentration of $[NHS\text{-}type\ alcohol]$ observed when the hydrolysis reaction has reached completion. As such we can make a substitution and arrive at **Equation 7**:

Equation 7.

$$\ln([NHS\text{-}type\ alcohol]_{final} - [NHS\text{-}type\ alcohol]) - \ln[NHS\text{-}type\ alcohol]_{final} = -kt$$

$[NHS\text{-}type\ alcohol]_{final}$: The final concentration of any NHS-type alcohol observed since the start of the hydrolysis experiment.

Applying the Beer-Lambert law of $A = \varepsilon cl$, and by treating the path length as 1 cm, we can construct **Equation 8**:

$$\ln\left\{(\frac{A_{268\,nm})_{final}}{\varepsilon} - \frac{(A_{268\,nm})}{\varepsilon}\right\} - \ln\left\{\frac{(A_{268\,nm})_{final}}{\varepsilon}\right\} = -kt$$ **Equation 8.**

$(A_{268\,nm})_{final}$: The absorbance at 268 nm observed when the hydrolysis reaction has reached completion.

$A_{268\,nm}$: The absorbance at 268 nm.

ε : The extinction coefficient of the NHS-type alcohol at 268 nm

This can be expanded and rearranged to yield **Equation 9**:

$$\ln\{(A_{268\,nm})_{final} - (A_{268\,nm})\} = -kt + \ln\{(A_{268\,nm})_{final}\}$$ **Equation 9.**

By referring to the variable $\{(A_{268\,nm})_{final} - (A_{268\,nm})\}$ as "$InvertedAbs_{(268\,nm)}$", we arrive at **Equation 10**.

$$\ln\{InvertedAbs_{(268\,nm)}\} = -kt + \ln\{(A_{268\,nm})_{final}\}$$ **Equation 10.**

Equation 10 informs us that, for values of t for which the hydrolysis reaction is still occurring, a plot of $\ln\{InvertedAbs_{(268\,nm)}\}$ against t will yield a straight line with a gradient of $-k$. Importantly, **Equation 10** allows for high-throughput screening of values of k under a range of conditions, as the use of this equation does not require either the extinction coefficient of the NHS-type alcohol (which varies with pH) or the initial concentration of $[Sulfo\ NHS\ ester]_0$ to be known, and can yield a value of k purely by tracking relative changes in $A_{268\,nm}$ over the course of the hydrolysis reaction.

Experimental setup data processing

The assay of Sulfo (and C-Sulfo) ester hydrolysis kinetics via UV-vis spectroscopy was performed using a CLARIOstar^{Plus} platereader (Suppler: BMG LABTECH) loaded with a UV-star® microplate (96 well, COC, F-bottom, chimney well, µCLEAR, clear) (Suppler: Greiner Bio-One). The layout of this plate is as follows:

Well	1	2	3	4	5	6	7	8	9	10	11	12
A												
B												
C												
D												
E												
F												
G												
H												

Figure S 64. The layout of a UV-star® 96 well microplate.

The wells we set up such in the following pattern as rapidly as possible using Gilson multichannel pipettes.

AX: Buffer (to be used as a blank)

BX: Buffer + sample of Sulfo-NHS alcohol **2** (such that $Abs_{268\ nm}$ was < 1)

CX: Buffer + sample of C-Sulfo-NHS alcohol **3** (such that $Abs_{268\ nm}$ was < 1)

DX: Buffer + 0.1 mM isobutyric acid (to check that isobutyric acid does not contribute to $Abs_{268\ nm}$)

EX: Buffer + 0.1 mM Sulfo-NHS ester **8**

FX: Buffer + 0.1 mM C-Sulfo-NHS ester **9**

The buffers investigated were: pH 5 sodium acetate, pH 6 BisTris, pH 6 MES, pH 6 sodium phosphate, pH 7 sodium phosphate, pH 7 BisTris, pH 8 sodium phosphate, pH 8 sodium borate, pH 9 sodium borate. The temperature of the lab was 22 °C.

The total volume delivered to each well was 300 µL. The final concentration of buffer salt in each sample was 100 mM. Organic molecules were delivered to the wells using DMSO stock solutions such that every well bore 10% DMSO v/v.

The ester Sulfo-NHS and C-Sulfo-NHS ester used in this experiment were **8** and **9** respectively. These esters were chosen as, being based on a simple isobutyric acid unit, they did not have any notable UV-vis absorbances of their own (which could have complicated the assay).

The CLARIOstar^{Plus} plate reader was set to automatically adjust and report $Abs_{268\ nm}$ values as 1 cm pathlength absorbances using the pathlength correction function before the kinetic run began. During the acquisition of the kinetic data, each well was sampled once a minute.

Over the course of the experiment, sample evaporation (which would induce error by causing the samples to become more concentrated over time) was accounted for by tracking the linear drift in the absorbance of the samples containing just Sulfo-NHS alcohol **2** or C-Sulfo-NHS alcohol **3**. This enabled time-dependent correction factors (Cf_t) to be calculated for each time increment.

Multiplication of the raw experimental data by $\mathrm{Cf_t}$ thereby partially corrects the time-dependent increases in the observed $\mathrm{Abs}_{268\ \mathrm{nm}}$ values caused by sample evaporation. In spite of this, it should be noted that the most accurate datapoints will still be those recorded at lower values of t, as any evaporation-induced errors uncompensated for by the application of $\mathrm{Cf_t}$ will be smaller.

After the application of $\mathrm{Cf_t}$ to the raw experimental data it becomes possible to observe plateaus in the values of $A_{268\ nm}$ reached for the wells which contained Sulfo-NHS ester (or C-Sulfo-NHS ester). The $A_{268\ nm}$ values of this plateaus can be taken as values for $(A_{268\ nm})_{final}$ in **Equation 9**, which allows values for $InvertedAbs_{(268\ nm)}$ to be calculated for each timepoint. Plots of $\ln\{InvertedAbs_{(268\ nm)}\}$ against t can now be constructed for each of the wells that contained a Sulfo-NHS ester (or C-Sulfo-NHS ester). The gradients of the linear portions of these plots can be used to yield values of k (**Equation 10**). The half-lives, $t_{\frac{1}{2}}$, of the NHS-type esters can thereafter be calculated from k using **Equation 11**.

$$t_{\frac{1}{2}} = \frac{\ln 2}{k}$$ **Equation 11**.

Experimental data

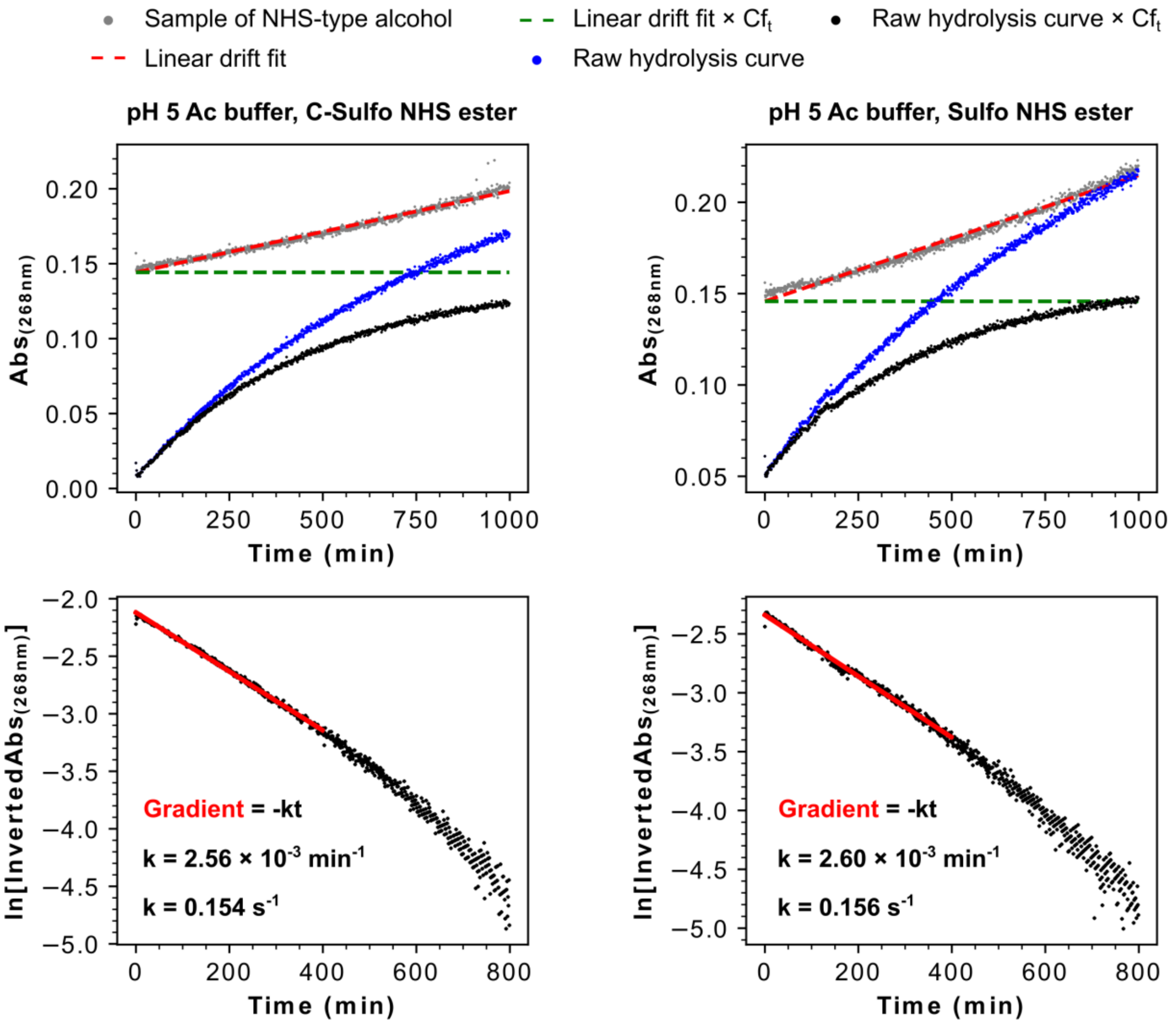

Figure S 65. UV kinetics data for the degradation of C-Sulfo-NHS ester **9** (**Left**) and Sulfo-NHS ester **8** (**Right**) in pH 5 100 mM sodium acetate buffer (with 10% v/v DMSO).

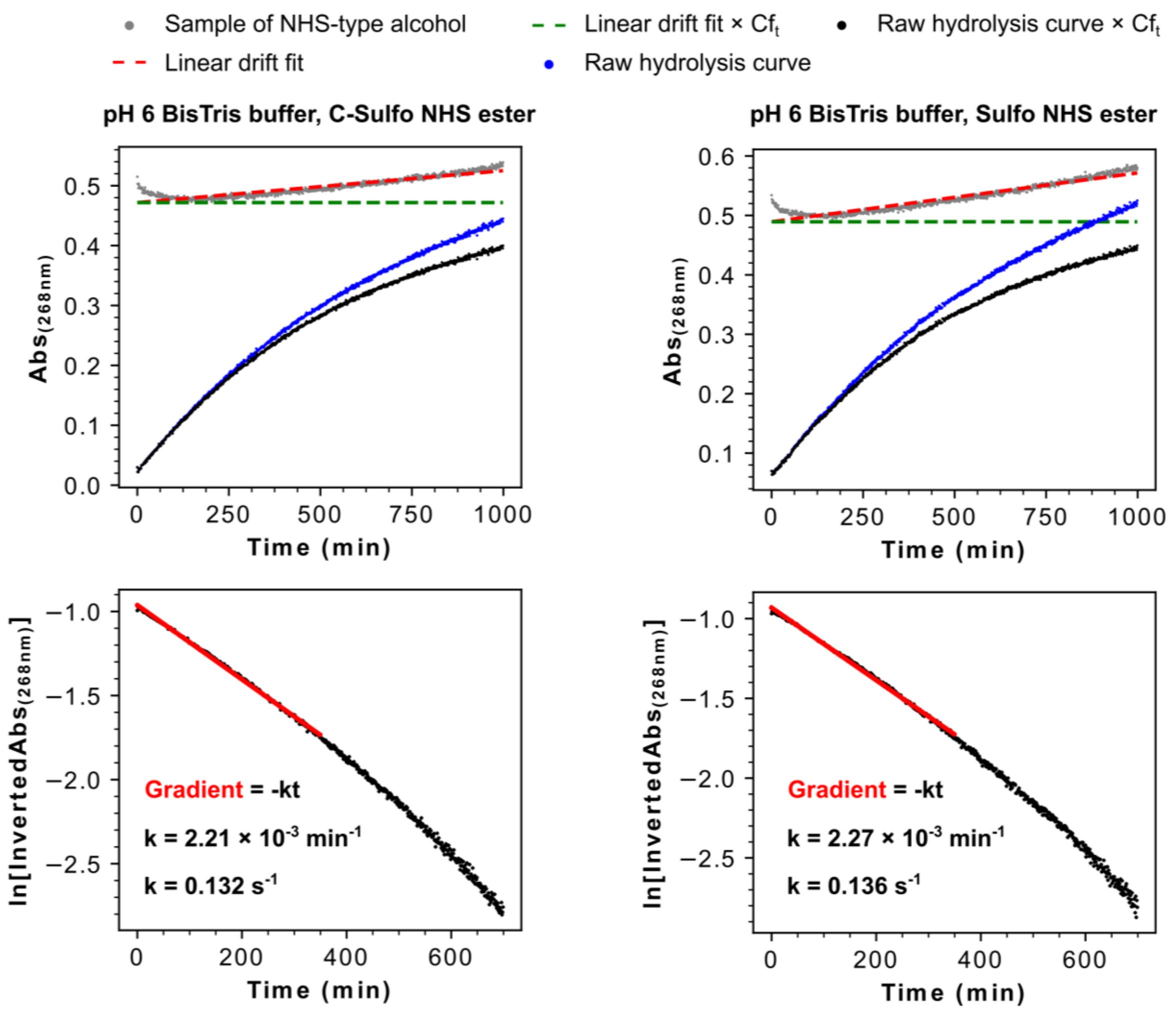

Figure S 66. UV kinetics data for the degradation of C-Sulfo NHS ester **9** (**Left**) and Sulfo NHS ester **8** (**Right**) in pH 6 100 mM BisTris buffer (with 10% v/v DMSO).

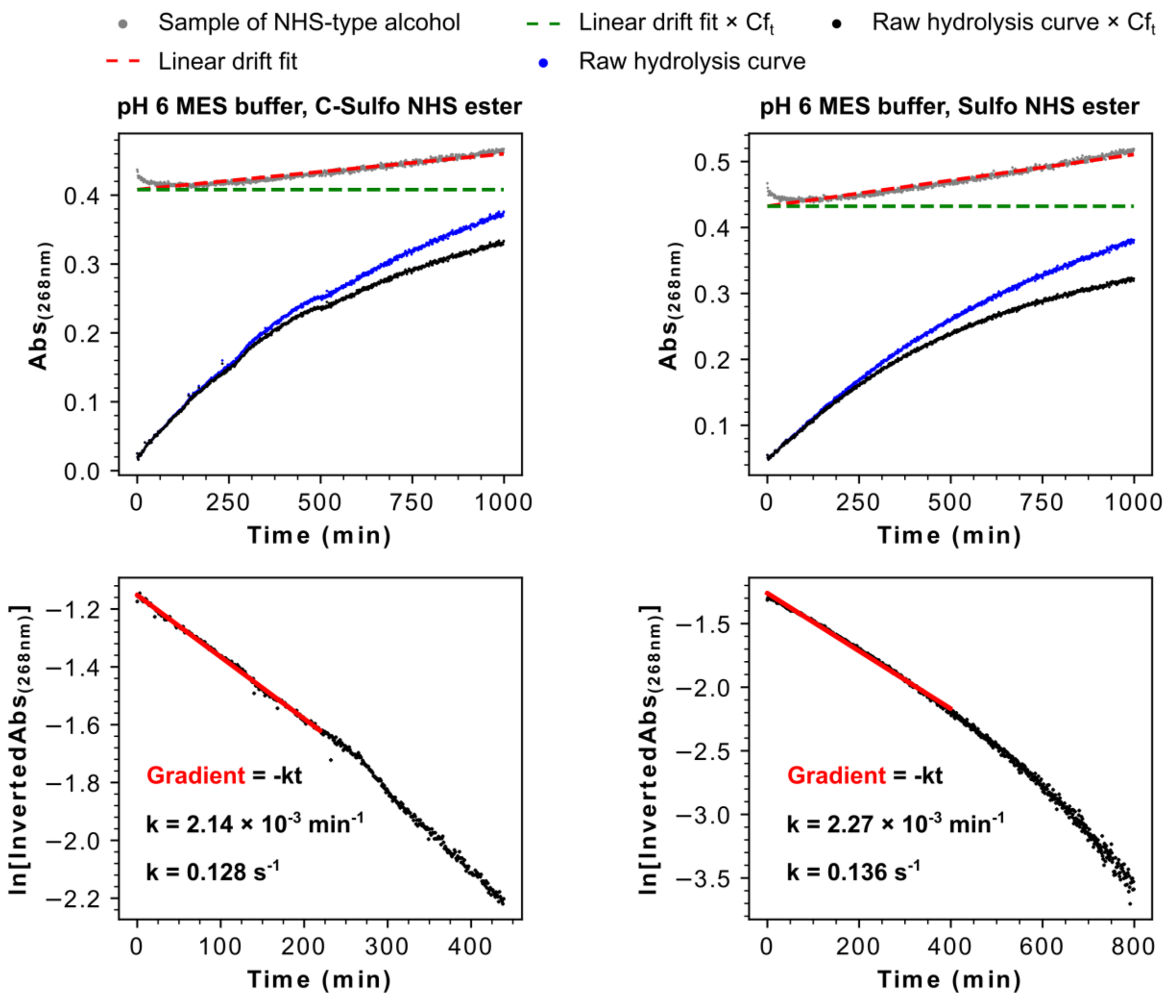

Figure S 67. UV kinetics data for the degradation of C-Sulfo NHS ester **9** (**Left**) and Sulfo NHS ester **8** (**Right**) in pH 6 100 mM MES buffer (with 10% v/v DMSO).

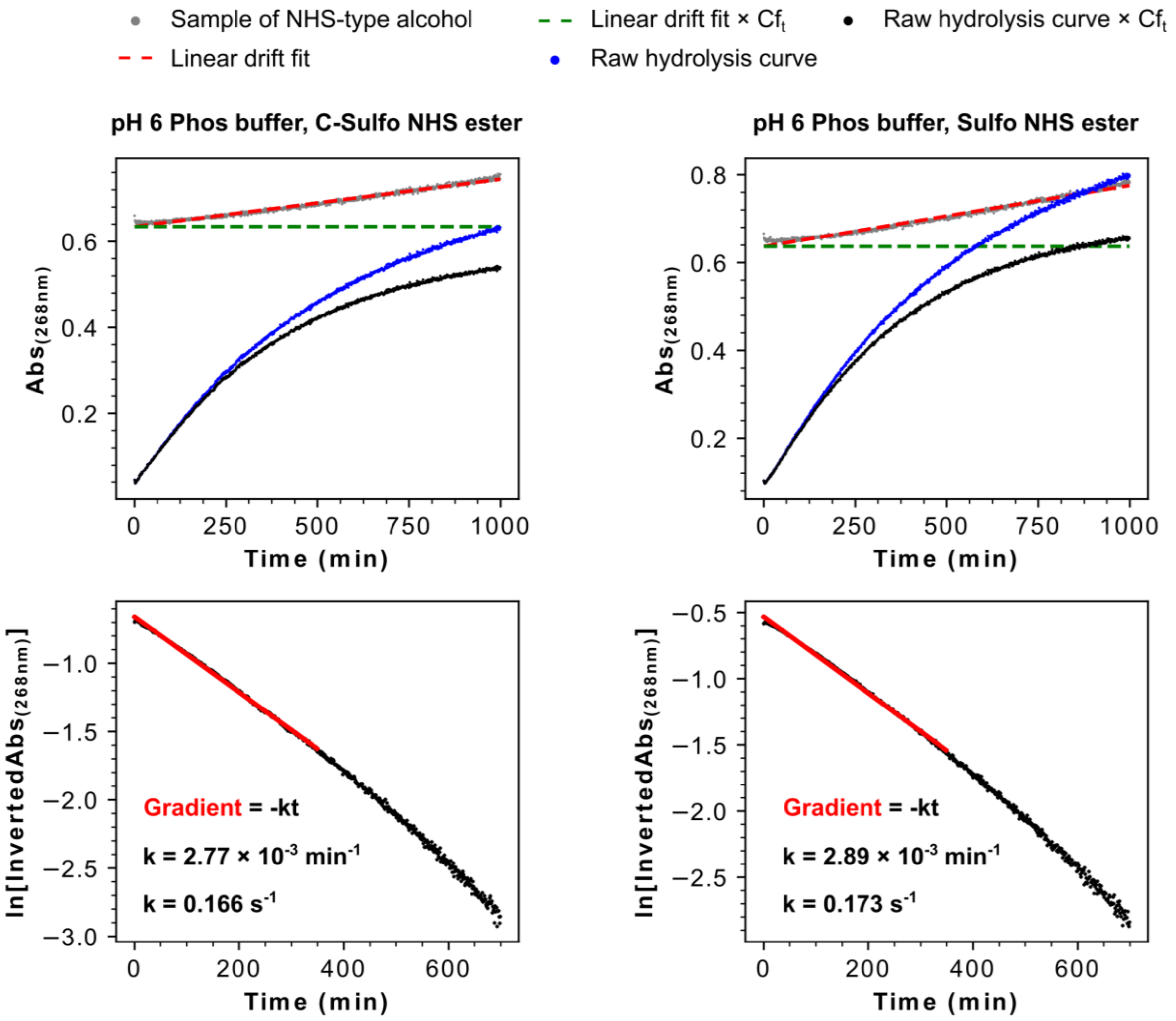

Figure S 68. UV kinetics data for the degradation of C-Sulfo NHS ester **9** (**Left**) and Sulfo NHS ester **8** (**Right**) in pH 6 100 mM sodium phosphate buffer (with 10% v/v DMSO).

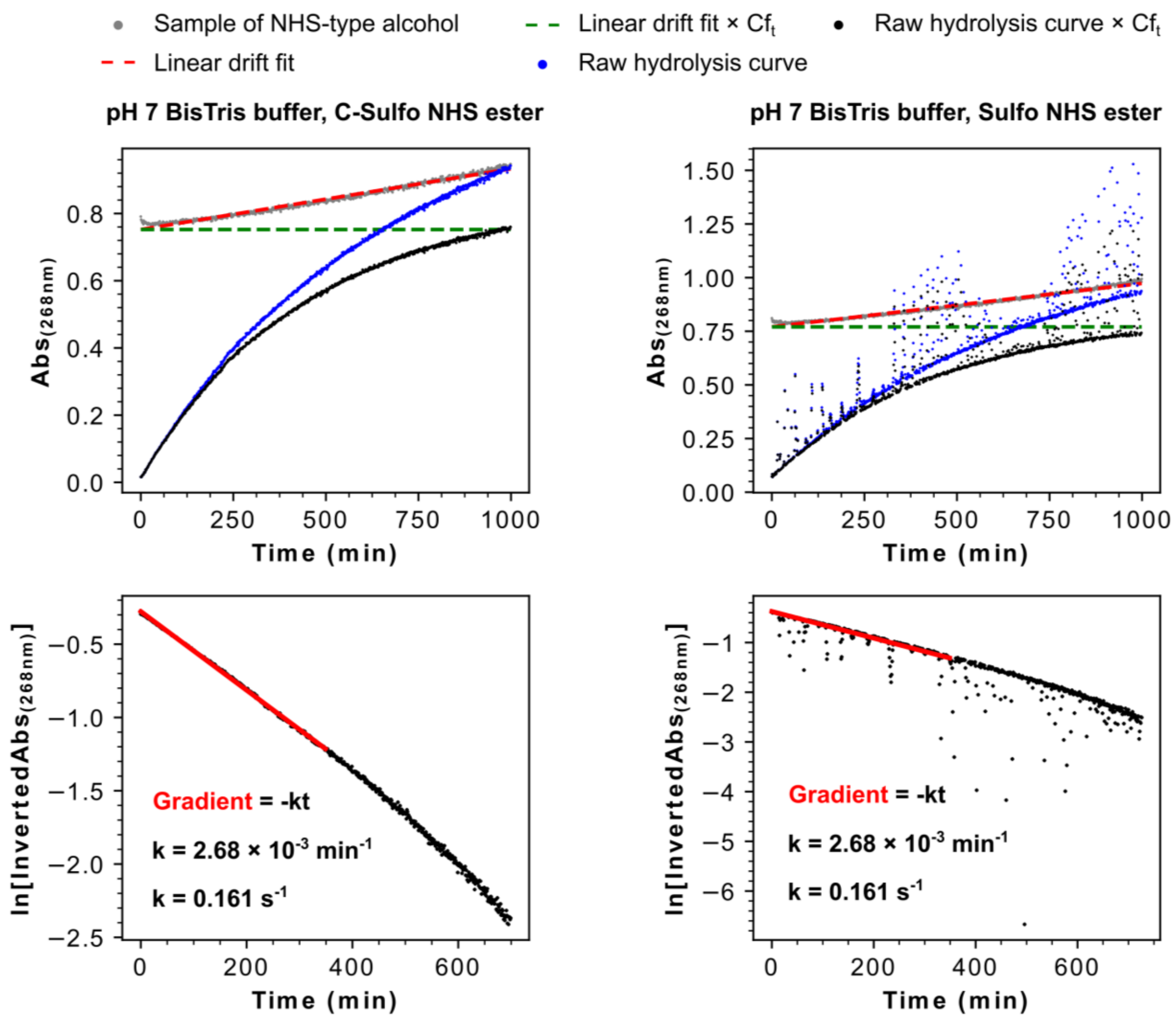

Figure S 69. UV kinetics data for the degradation of C-Sulfo NHS ester **9** (**Left**) and Sulfo NHS ester **8** (**Right**) in pH 7 100 mM BisTris buffer (with 10% v/v DMSO).

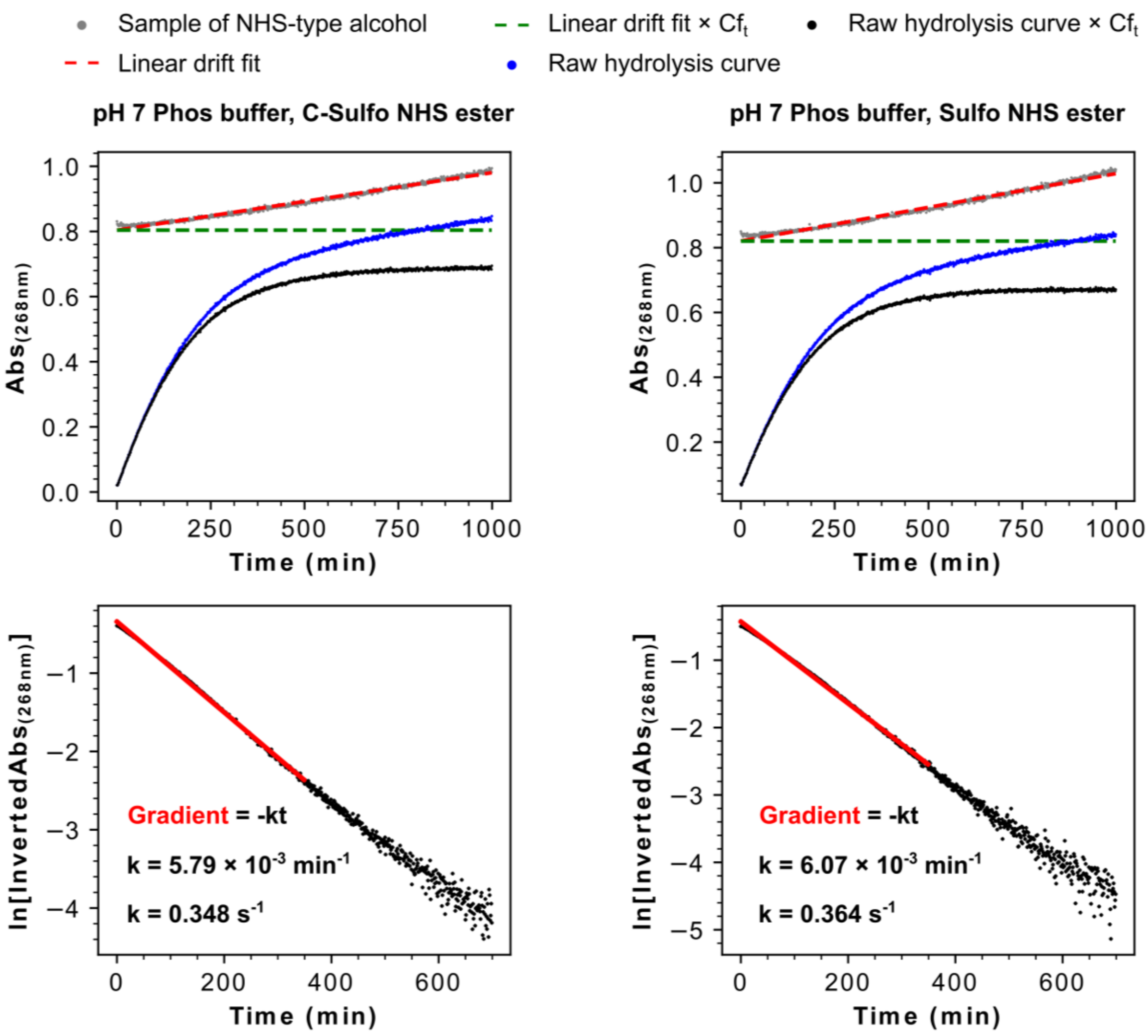

Figure S 70. UV kinetics data for the degradation of C-Sulfo NHS ester **9** (**Left**) and Sulfo NHS ester **8** (**Right**) in pH 7 100 mM sodium phosphate buffer (with 10% v/v DMSO).

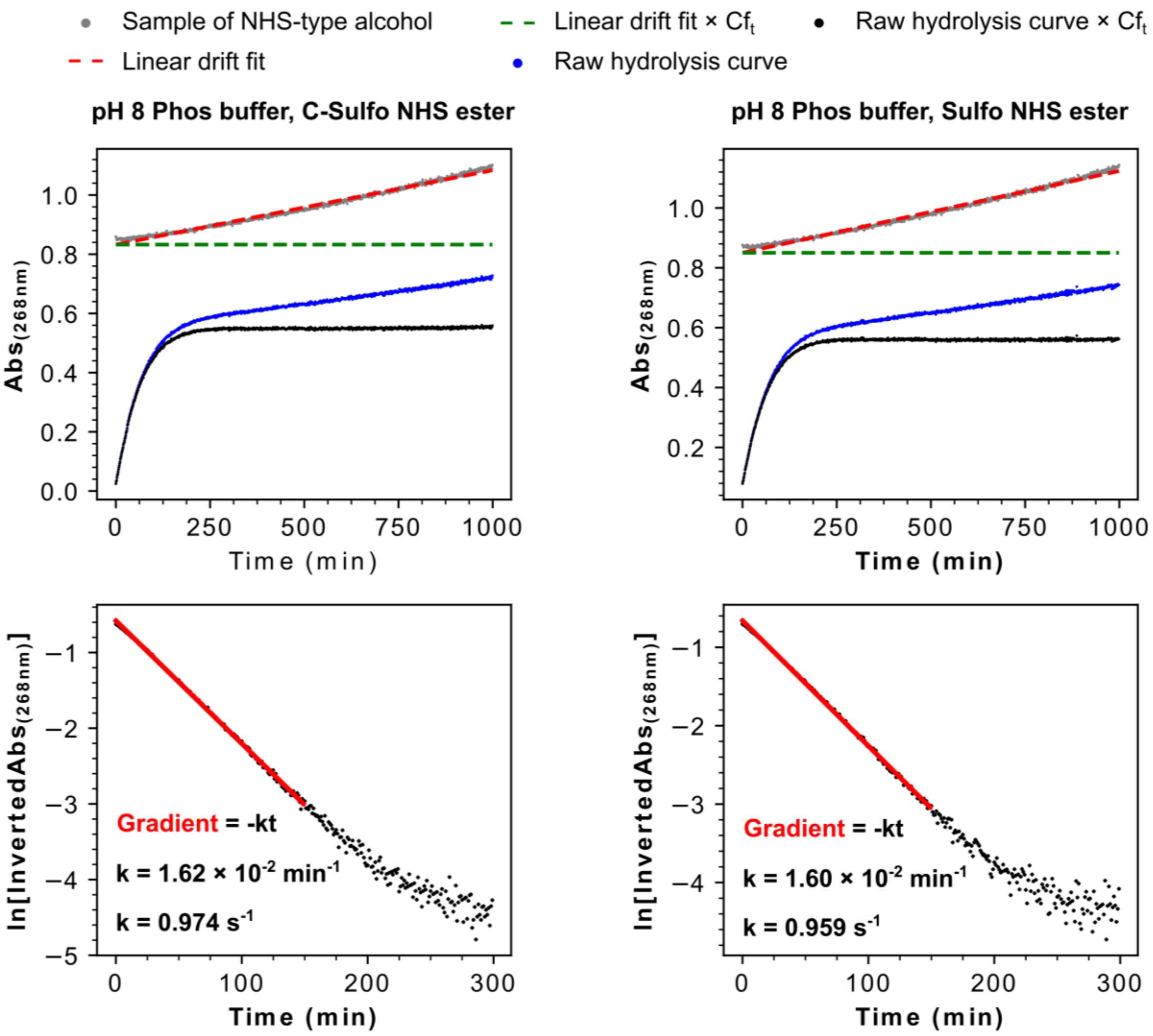

Figure S 71. UV kinetics data for the degradation of C-Sulfo NHS ester **9** (**Left**) and Sulfo NHS ester **8** (**Right**) in pH 8 100 mM sodium phosphate buffer (with 10% v/v DMSO).

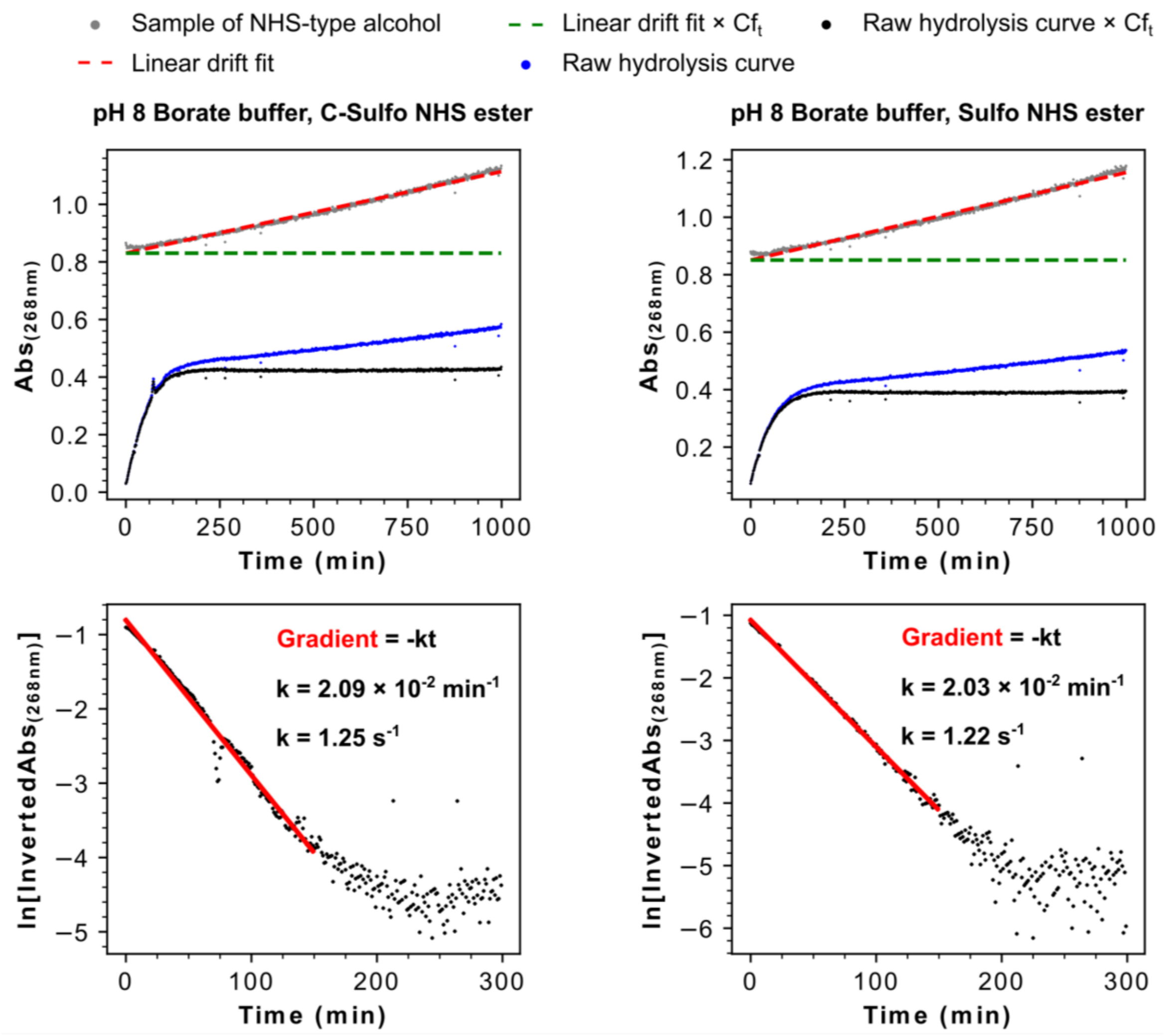

Figure S 72. UV kinetics data for the degradation of C-Sulfo NHS ester **9** (**Left**) and Sulfo NHS ester **8** (**Right**) in pH 8 100 mM sodium borate buffer (with 10% v/v DMSO).

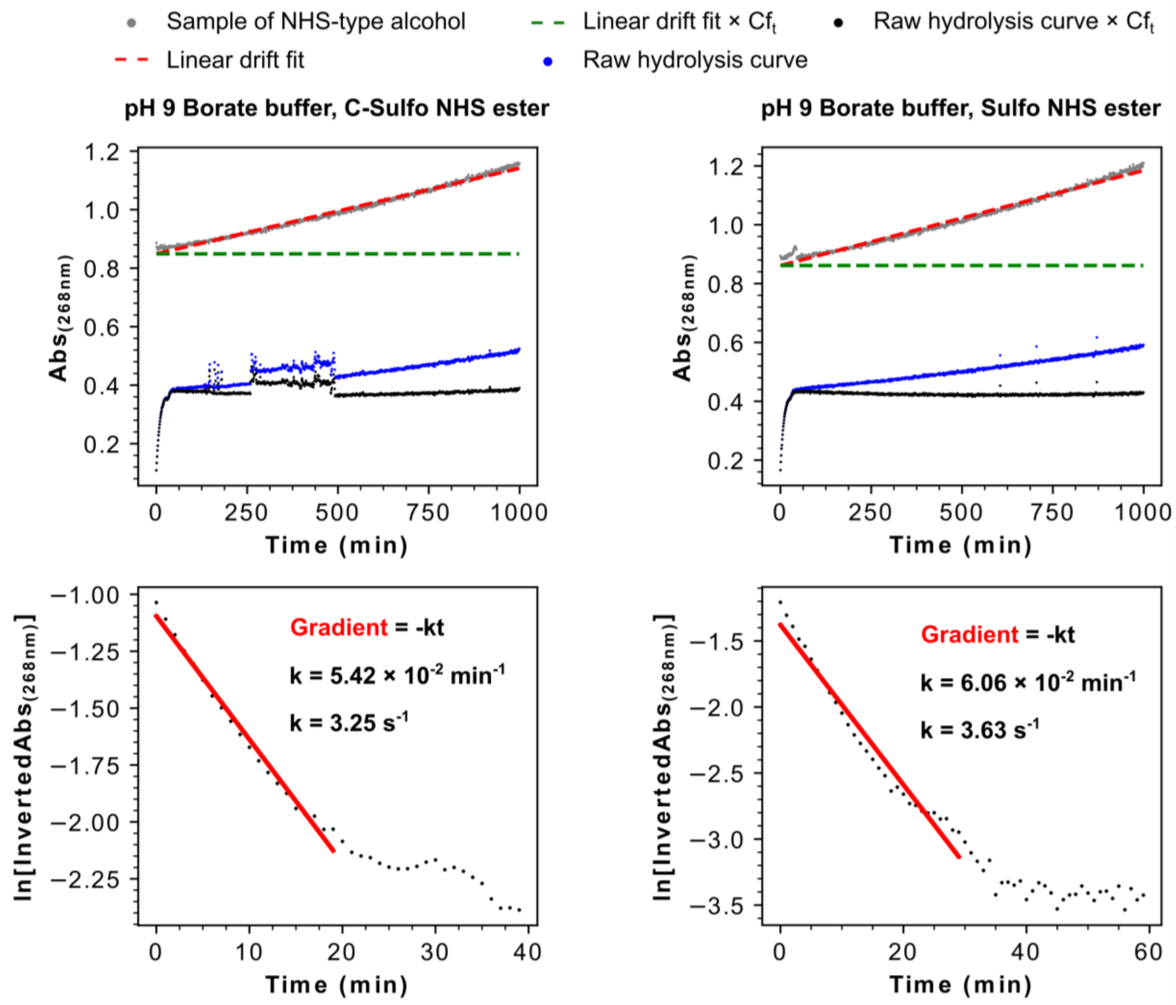

Figure S 73. UV kinetics data for the degradation of C-Sulfo NHS ester **9** (**Left**) and Sulfo NHS ester **8** (**Right**) in pH 9 100 mM sodium borate buffer (with 10% v/v DMSO).

Determining the selectivity of Sulfo NHS-type esters

Competition experiment setup

1 mL reaction solutions were prepared from appropriate stock solutions such that the final composition was 10 mM *N*-Acetyl-L-cysteine, 10 mM *N*α-Acetyl-L-lysine and 10 mM of **8**/**9** in 100 mM *N*-methylmorpholine (NMM) hydrochloride buffer at pH 8.3 or 7.5. The stock solutions of *N*-Acetyl-L-cysteine and **8**/**9** were prepared using DMF, and upon the delivery of these stock solutions to the reaction mixture, the reaction solution became 10% v/v DMF.

Reactions were incubated at rt in darkness for 2 hours, after which time 0.5 mL of the reaction solutions were removed, diluted with HPLC-grade water and lyophilized. The residues yielded after lyophilization were dissolved in 0.5 mL methanol-d_4 and analysed via ^{1}H-NMR. The remaining 0.5 mL reaction solutions were analysed by LC-MS.

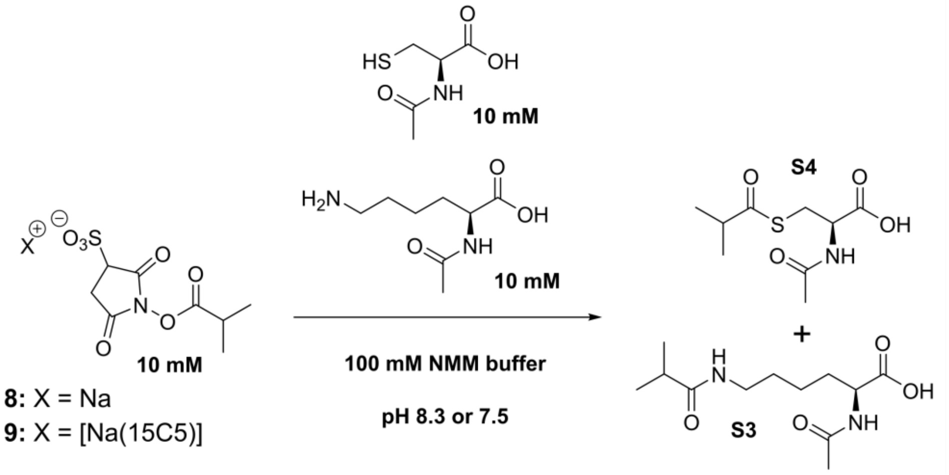

Figure S 74. Competition experiment between cysteine and lysine side-chains for esters **8** and **9**.

Analysis via LC-MS

Calibration curves were constructed by spiking known concentrations of the potential amide and thioester products, **S3** and **S4**, into 100 mM N-methylmorpholine hydrochloride buffer from DMF stock solutions. Upon the delivery of these stock solutions to buffer, the resultant solution became 10% v/v DMF.

Prior to analysis, 5 µL aliquots of these standard solutions were added to 45 µL of 1:1 water:acetonitrile (v/v) + 1% (v/v) formic acid solution. LC was performed using an Accucore™ C18 HPLC Column (Particle Size: 2.6 µm, Diameter: 3 mm, Length: 50 mm, Catalog Number: 17126-053030, Thermo Scientific™). Water (solvent A) and acetonitrile (solvent B), both containing 0.1% formic acid, were used as the mobile phase at a flow rate of 0.3 mL min^{-1}. MS was conducted in negative mode and LC traces were measured via their total ion counts ("All-MS"). The LC gradient used was programmed as shown in **Figure S75**.

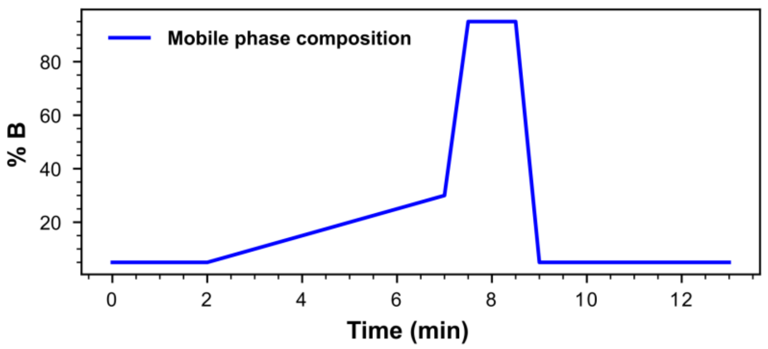

Figure S 75. The LC gradient used to resolve **S3** from **S4**.

Upon running a sample, two well-resolved peaks can be observed. The first peak corresponds to **S3**, whereas the second corresponds to **S4** (Figure SX). A plot of the areas under these peaks against the concentration of **S3** and **S4** present in each standard can be used to yield a calibration curve (Figure SX).

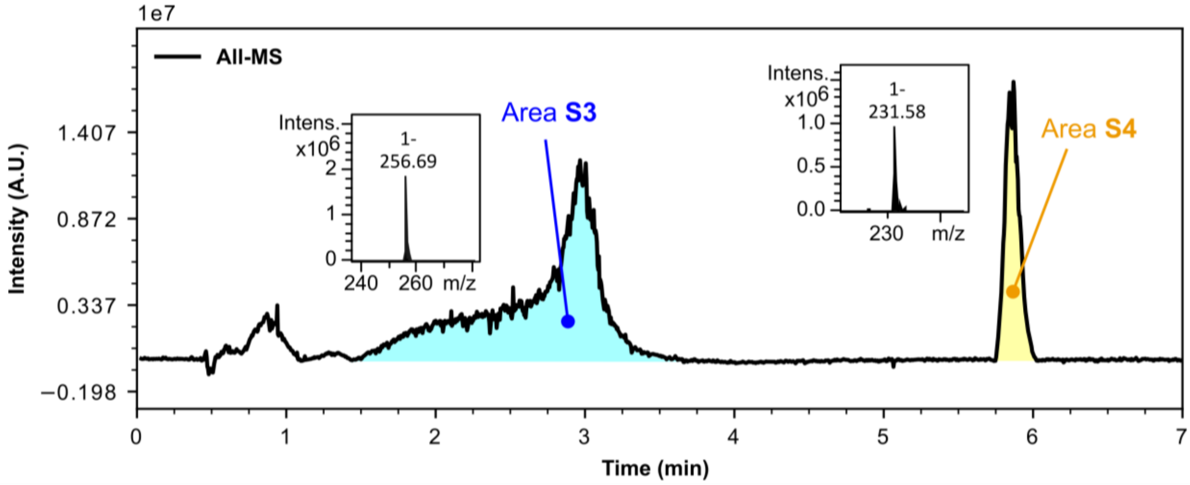

Figure S 76. Analysis of the LC-MS trace of a solution containing **S3** and **S4**.

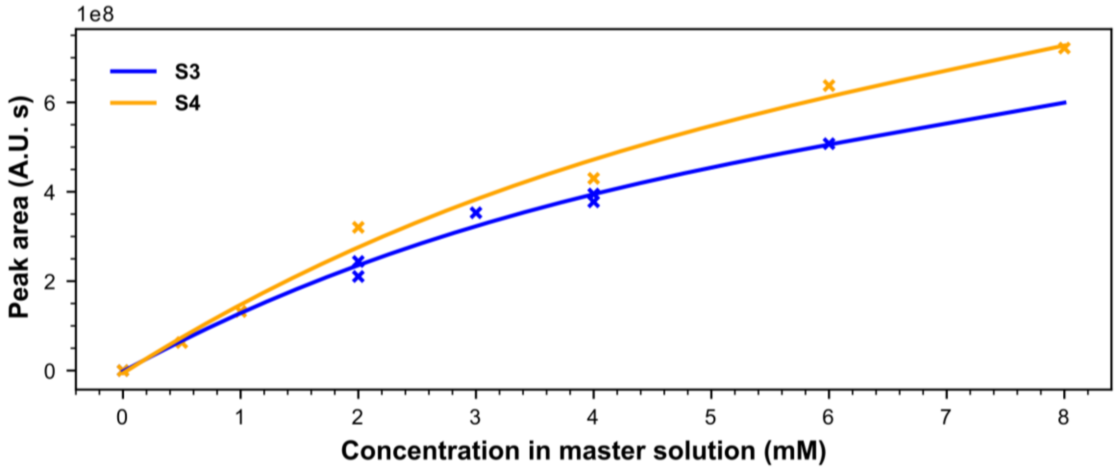

Figure S 77. A calibration curve of All-MS peak area vs concentration of **S3** or **S4**.

In order to analyse a reaction mixture, a 5 µL aliquot was added to 45 µL of 1:1 water:acetonitrile (v/v) + 1% (v/v) formic acid solution and LC-MS was performed as detailed above. Peak areas for **S3** and **S4** were then calculated, and the calibration curve was thereafter used to determine the concentration of **S3** and **S4** present in the reaction mixture. The results of this analysis are tabulated in **Table S2**.

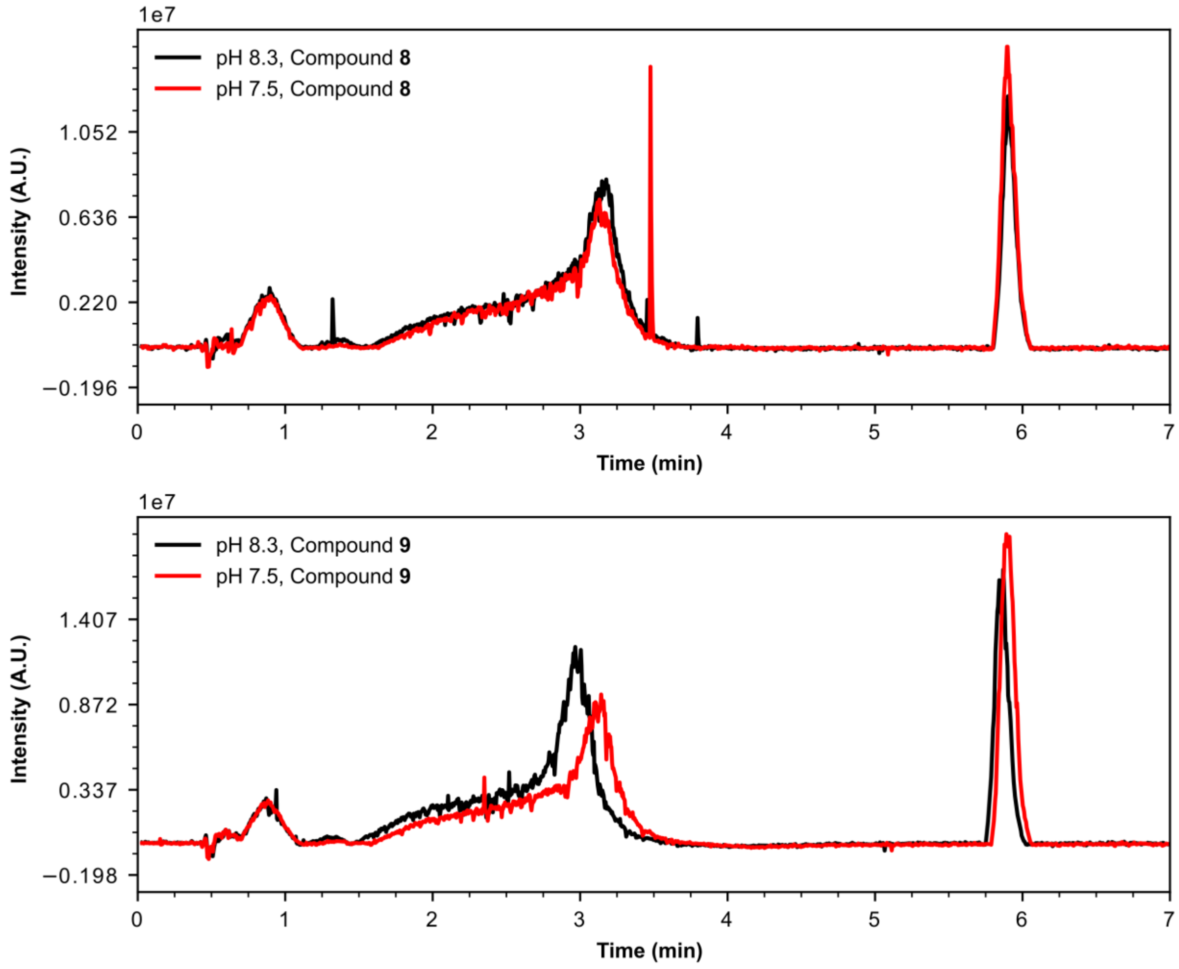

Figure S 78. LC-MS traces of the competition experiments involving **8**/**9**.

Table S2. Results of LC-MS analysis of the competition experiments involving **8**/**9**.

Experimental condition	**S3** Conc (mM)	**S4** Conc (mM)	**S3** Conc / **S4** Conc	**S3** yield (%)	**S4** yield (%)
pH 7.5, **8**	2.18	0.62	3.5	21.8	6.2
pH 8.3, **8**	2.56	0.50	5.1	25.6	5.0
pH 7.5, **9**	2.74	0.92	3.0	27.4	9.2
pH 8.3, **9**	3.57	0.76	4.7	35.7	7.6

Analysis via ^{1}H-NMR

Lyophilisation removes any isobutyric acid (or isobutyric acid salts) that were present in the reaction solution due the hydrolysis of ester **8** / **9**. Consequently, the region corresponding to isobutyrate protons will only be populated with signals arising from either **S3** or **S4** during ^{1}H-NMR analysis of the lyophilised extracts. Upon comparing the chemical shifts of these signals to those observed in pure solutions of **S3** or **S4** in methanol-d_4 (and using COSY), it was possible to assign these signals. The ratio of the integrals of these signals was then used to provide a measure of the selectivity of esters **8** and **9** for lysine side-chains over cysteine side-chains at both pH 8.3 and pH 7.5. The results of this analysis are tabulated in **Table S3**.

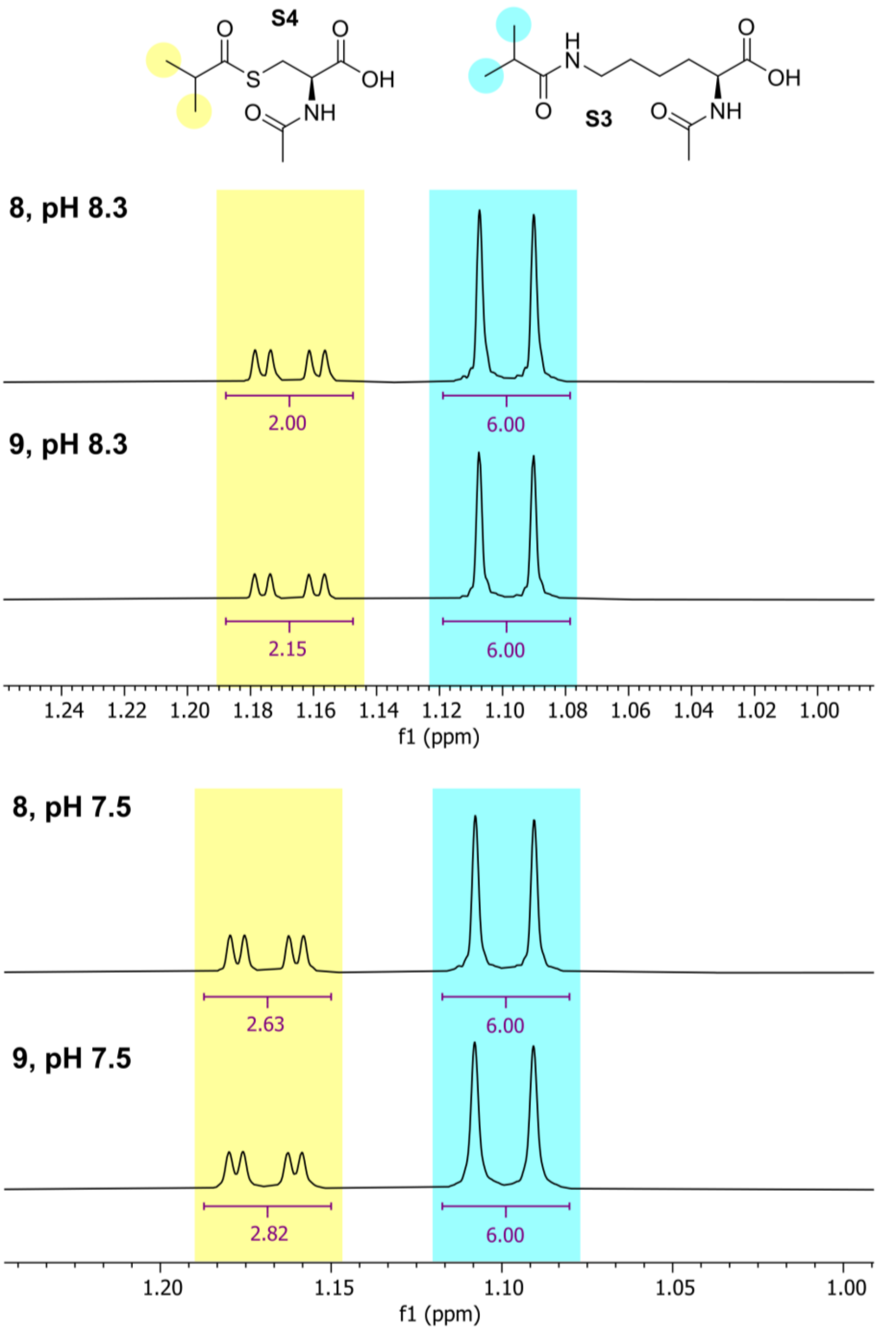

Figure S 79. ^{1}H-NMR analysis of the isobutyrate-derived proton signals arising from **S3** and **S4** formed during the competition experiments involving **8**/**9**.

Table S3. Results of ^{1}H-NMR analysis of the competition experiments involving **8**/**9**.

Experimental condition	Integral of **S3** isobutyrate signal / Integral of **S4** isobutyrate signal
pH 7.5, **8**	2.3
pH 8.3, **8**	3.0
pH 7.5, **9**	2.1
pH 8.3, **9**	2.8

Additional Figures

Relative hydrolysis rates and half-lives of Sulfo and C-Sulfo-NHS esters 8 and 9

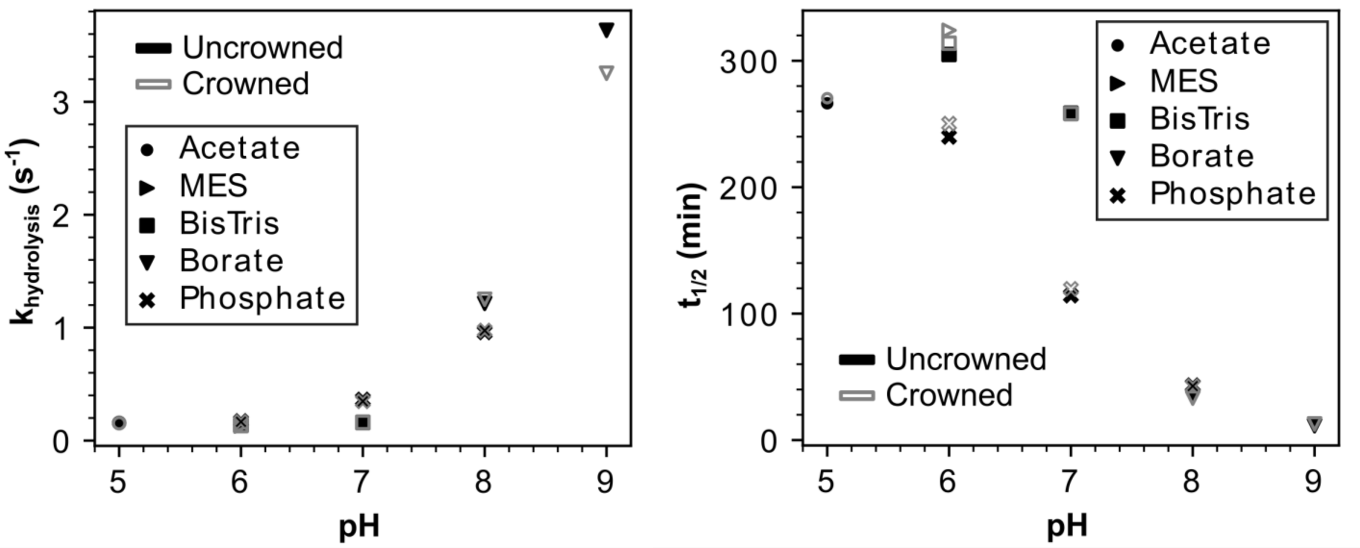

Figure S 80. The variation of the rate of hydrolysis (and associated half-lives) of akin Sulfo and C-Sulfo-NHS esters **8** and **9**. “Uncrowned” = data collected using Sulfo-NHS ester **8**. “Crowned” = data collected using C-Sulfo-NHS ester **9**.

Protein mass spectrometry of CjX183-D R51K bioconjugates

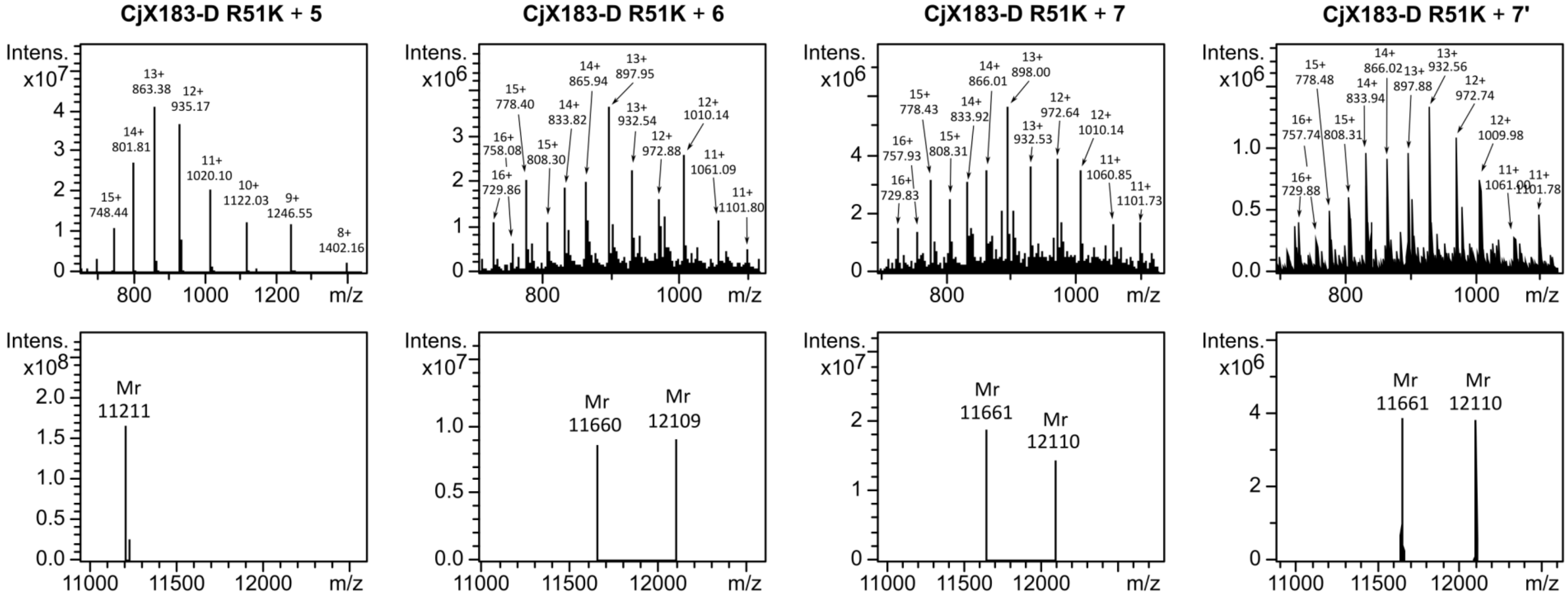

Figure S 81. Protein mass spectrometry analysis of CjX183-D R51K biconjugate samples.

Protein mass spectrometry of DsbA bioconjugates

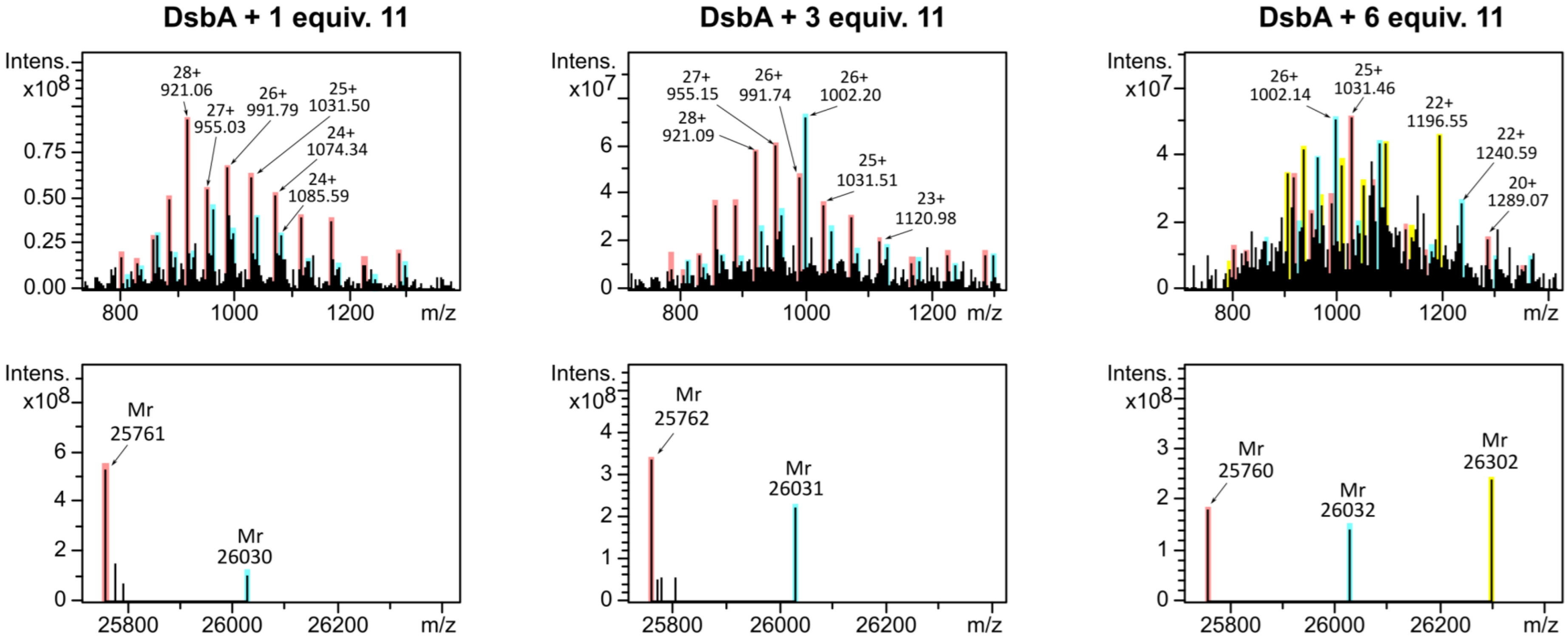

Figure S 82. Protein mass spectrometry analysis of DsbA bioconjugate samples prepared using **11**.

Crude ^{1}H-NMR spectra of esters derived from 4

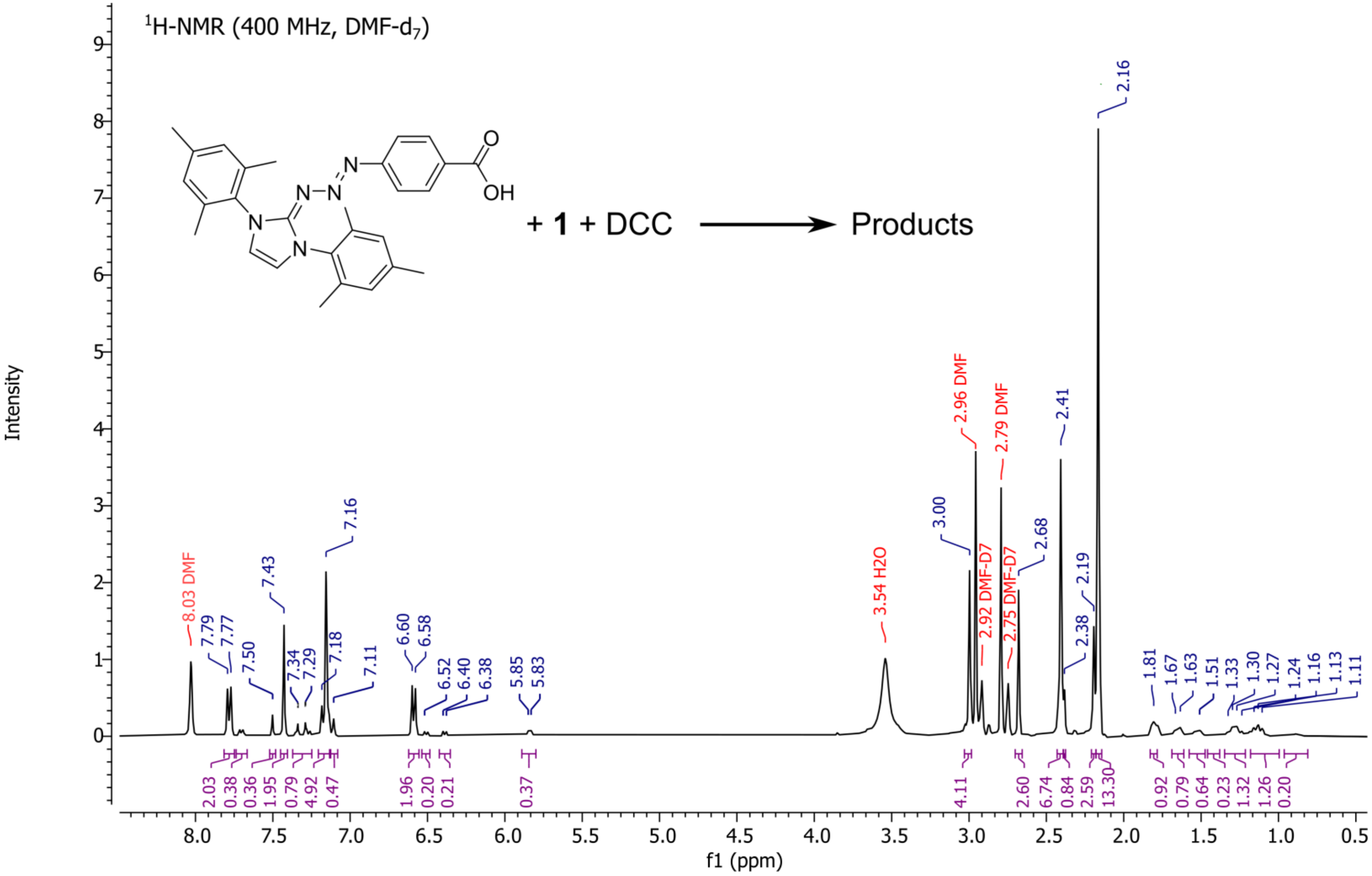

Figure S 83. ^{1}H-NMR analysis of the crude product mixture obtained from the reaction of triazabutadiene carboxylic acid **4** with NHS reagent **1** and DCC.

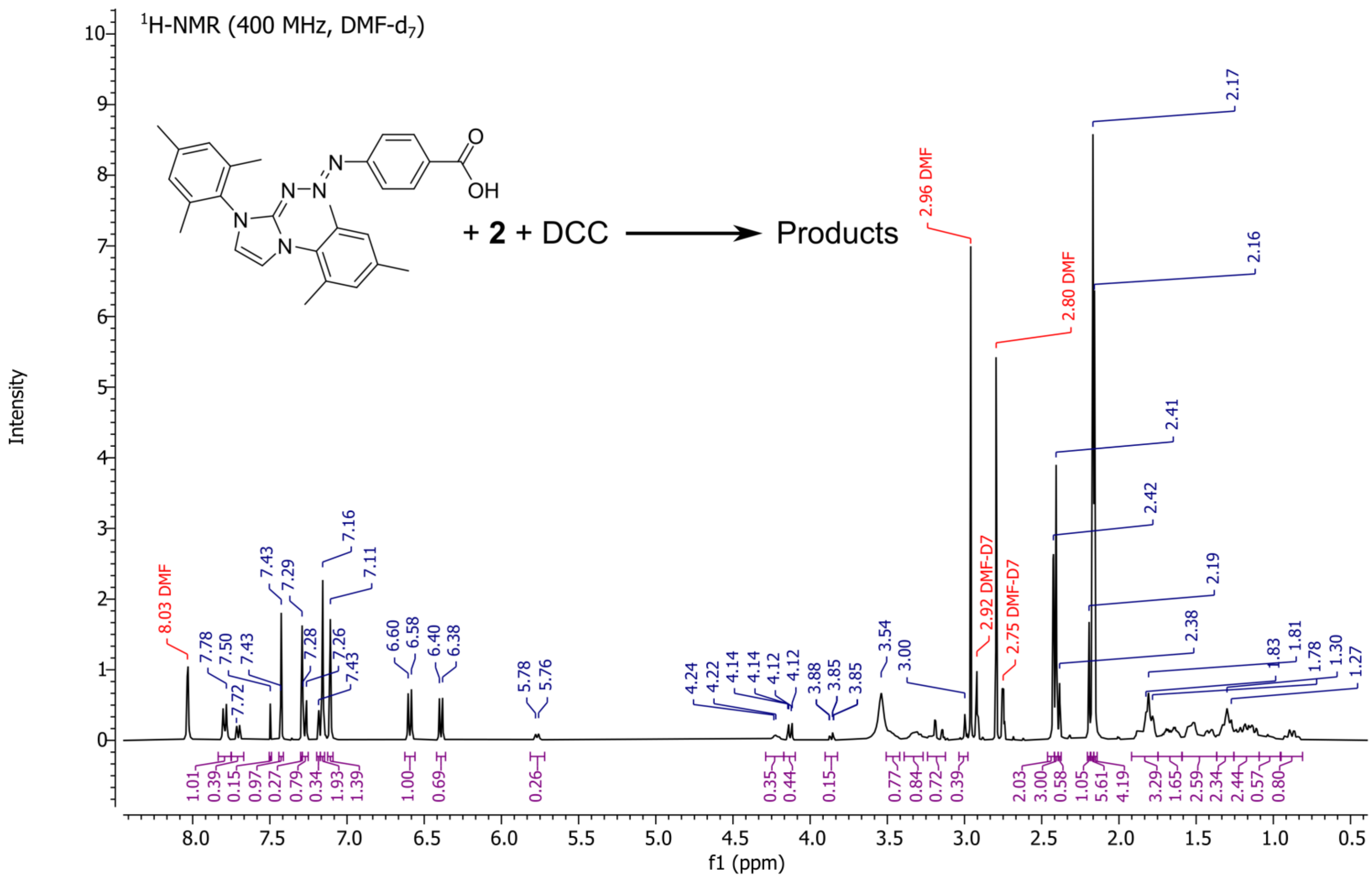

Figure S 84. ^{1}H-NMR analysis of the crude product mixture obtained from the reaction of triazabutadiene carboxylic acid **4** with NHS reagent **2** and DCC.

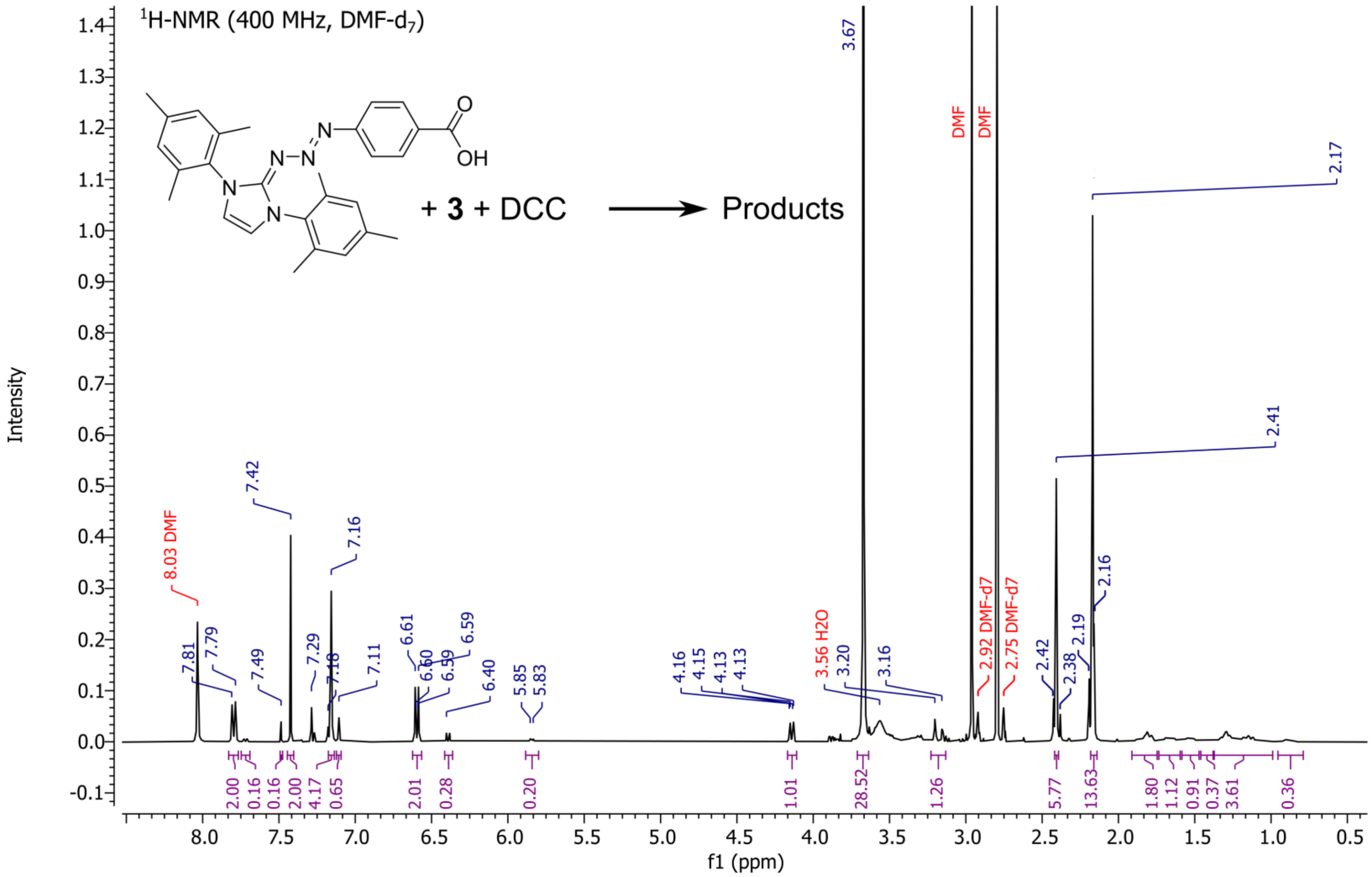

Figure S 85. ^{1}H-NMR analysis of the crude product mixture obtained from the reaction of triazabutadiene carboxylic acid **4** with NHS reagent **3** and DCC.

^{1}H-NMR and (ESI-HRMS) spectra of the urea side-product derived from 4

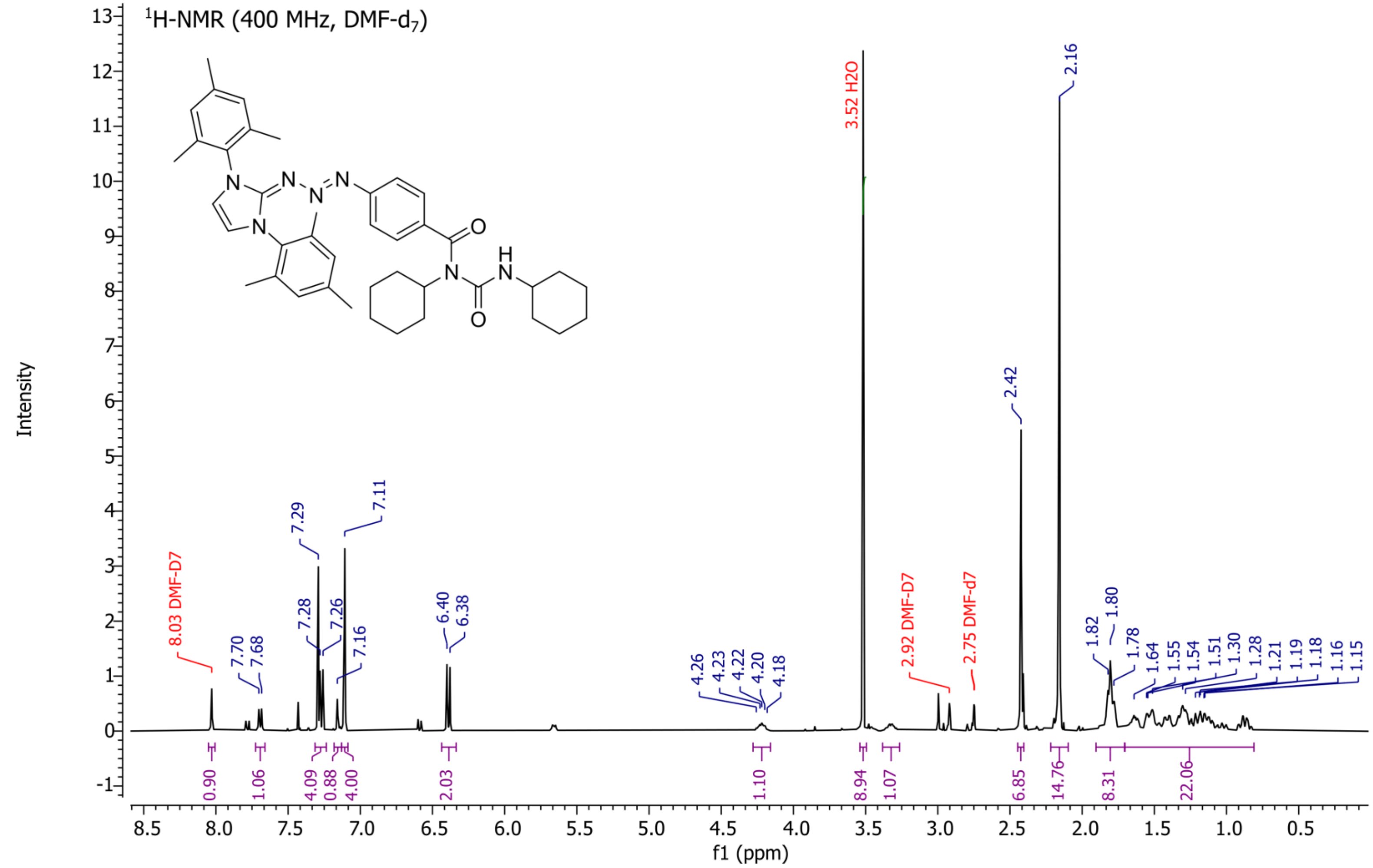

Figure S 86. ^{1}H-NMR of the urea side-product isolated after attempting the esterification of **4** with **2** and DCC.

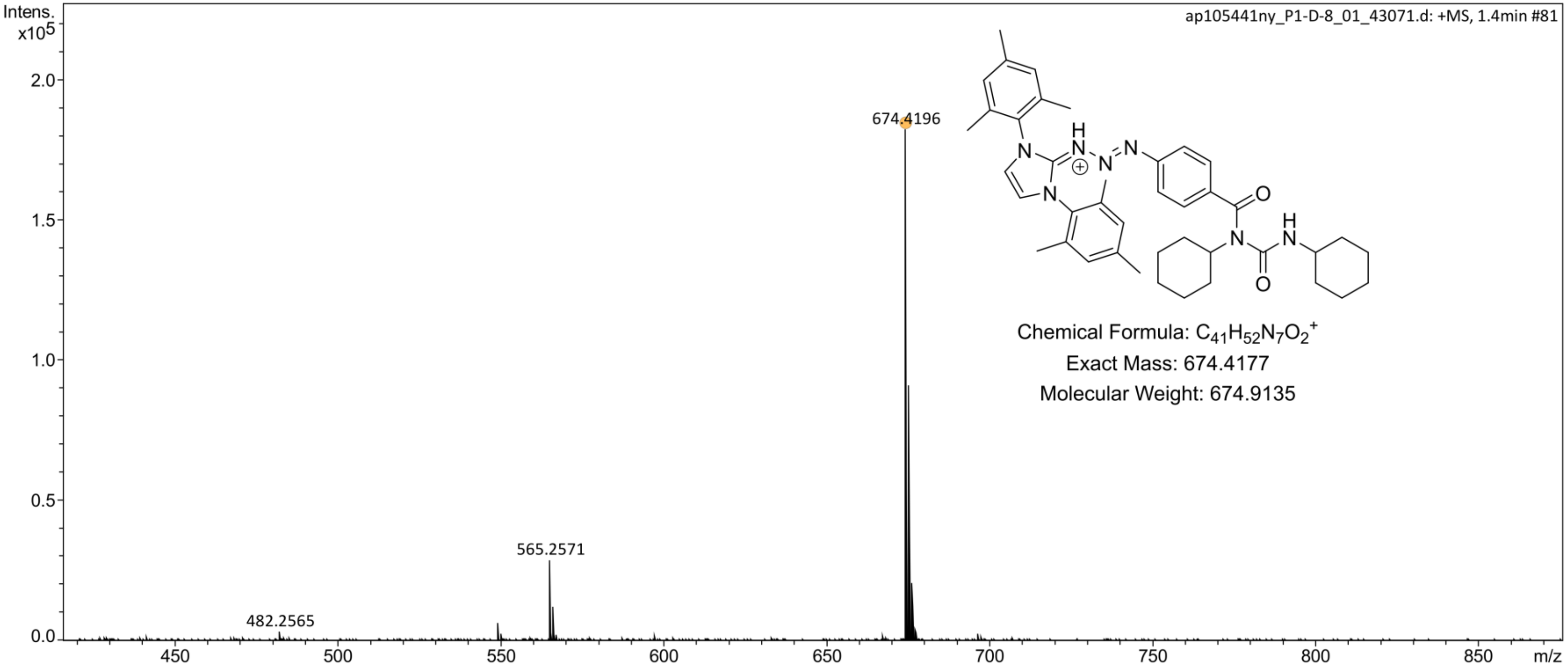

Meas. m/z	#	Ion Formula	m/z	err [ppm]	err [mDa]	mSigma	Mean err [ppm]
674.4196	1	C41H52N7O2	674.4177	-2.8	-1.9	12.1	-2.4

Figure S 87. (ESI)HRMS of the urea side-product isolated after attempting the esterification of **4** with **2** and DCC.

.

^{1}H-NMR spectra from selectivity study of 8 and 9

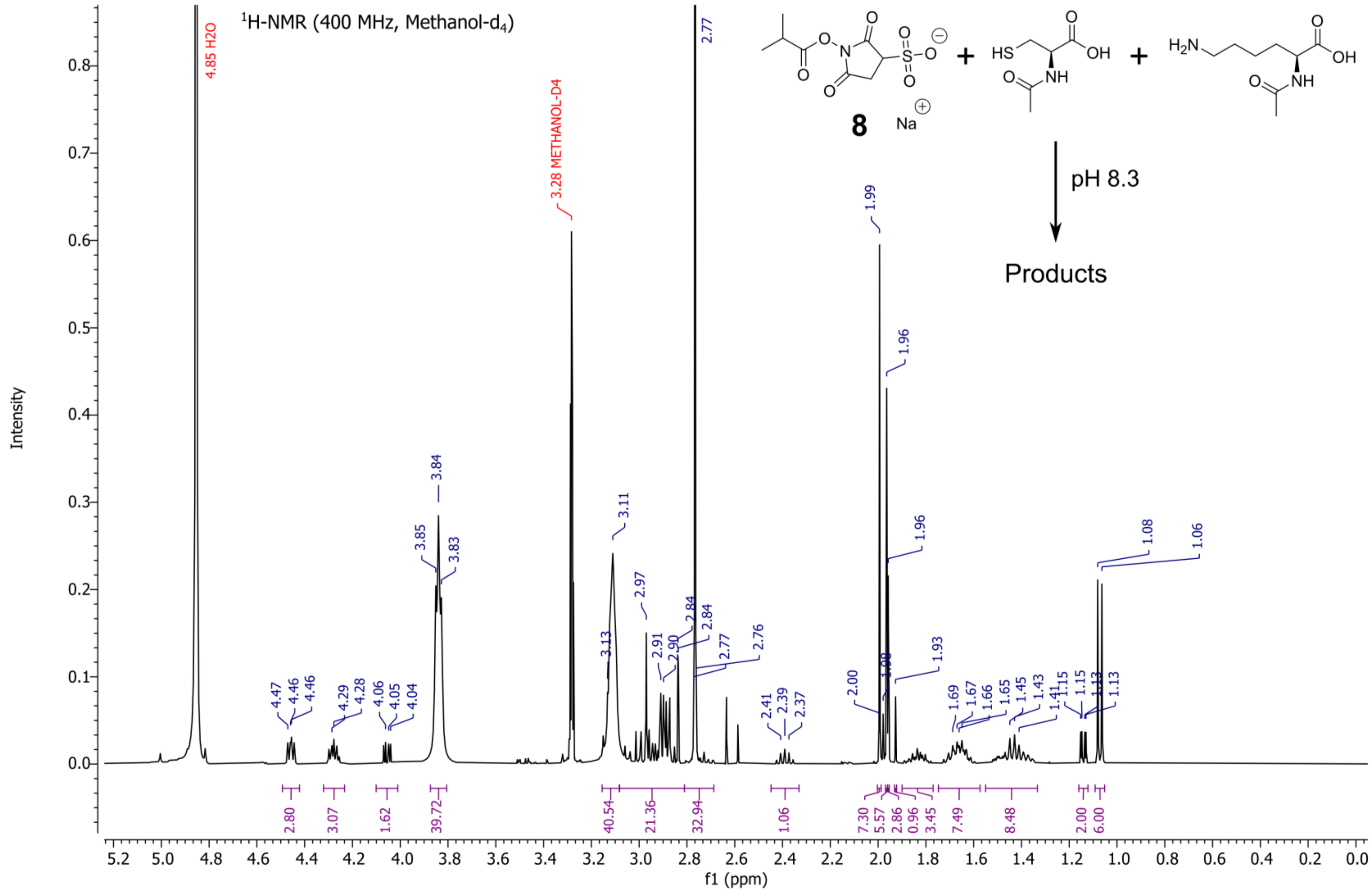

Figure S 88. ^{1}H-NMR analysis of the product mixture obtained from the reaction Sulfo-NHS ester **8** with *N*α-Acetyl-L-lysine and *N*-Acetyl-L-cysteine in pH 8.3 NMM buffer (after lyophilization).

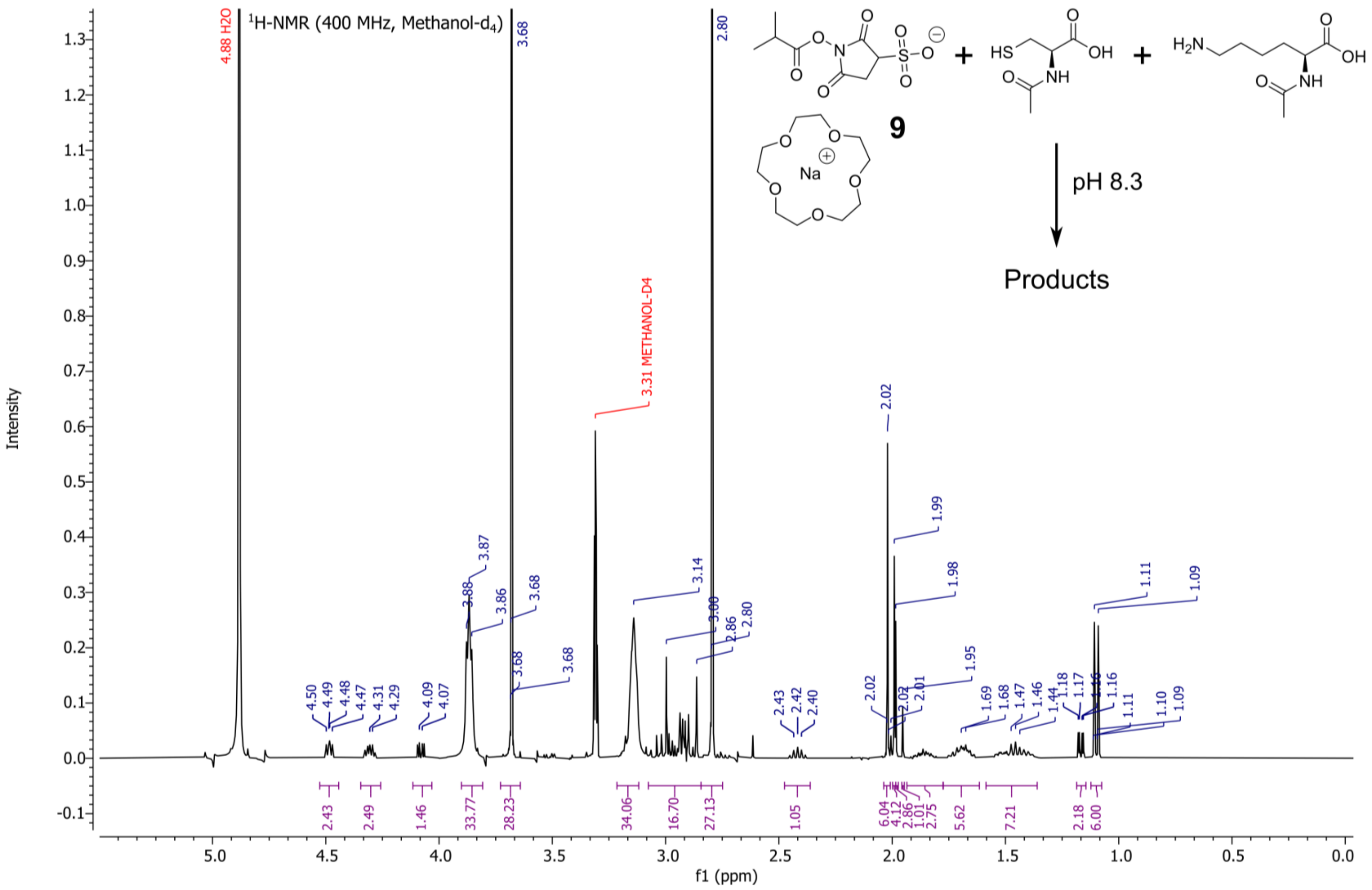

Figure S 89. ^{1}H-NMR analysis of the product mixture obtained from the reaction C-Sulfo-NHS ester **9** with *N*α-Acetyl-L-lysine and *N*-Acetyl-L-cysteine in pH 8.3 NMM buffer (after lyophilization).

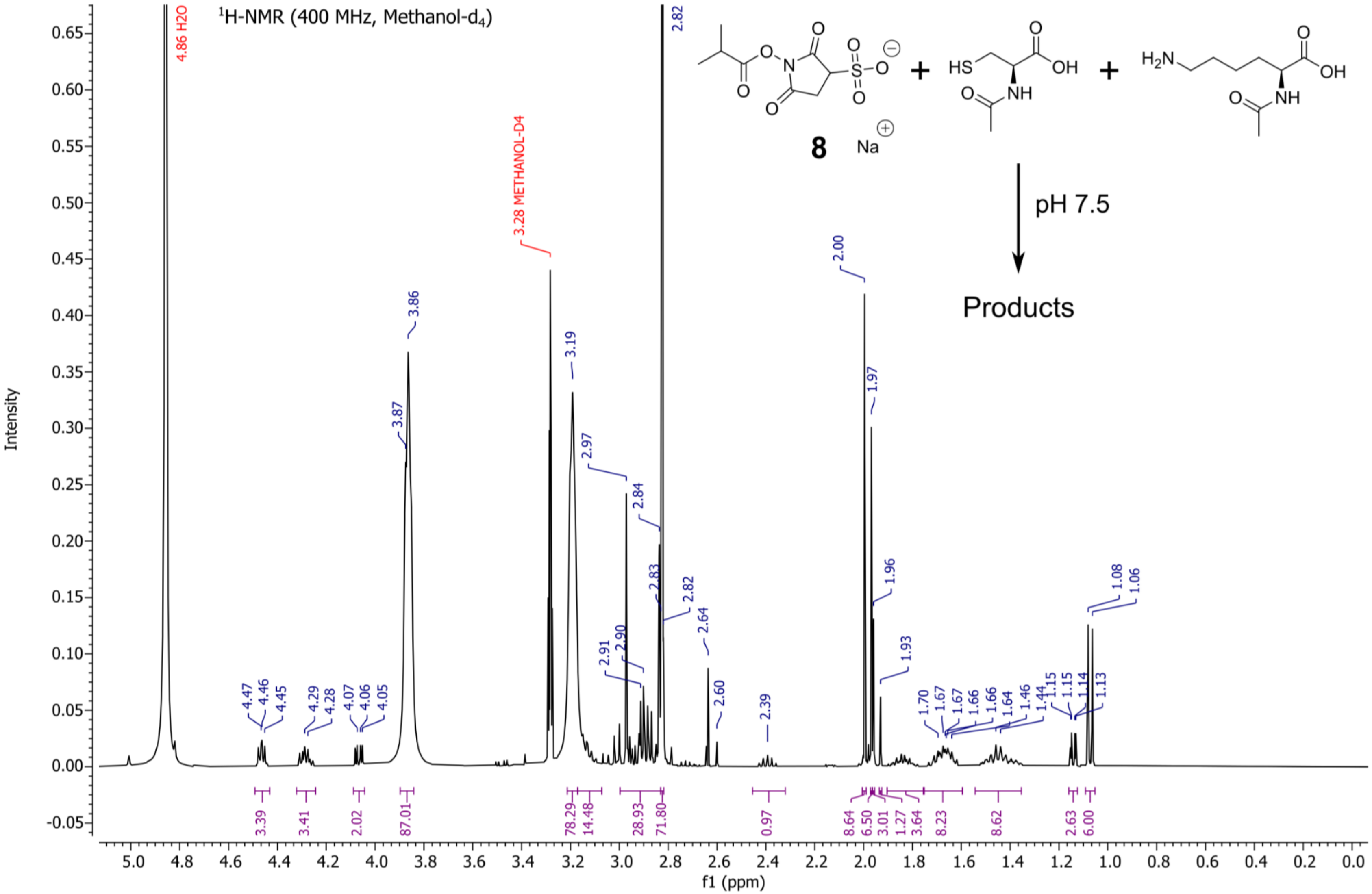

Figure S 90. ^{1}H-NMR analysis of the product mixture obtained from the reaction Sulfo NHS ester **8** with *N*α-Acetyl-L-lysine and *N*-Acetyl-L-cysteine in pH 7.5 NMM buffer (after lyophilization).

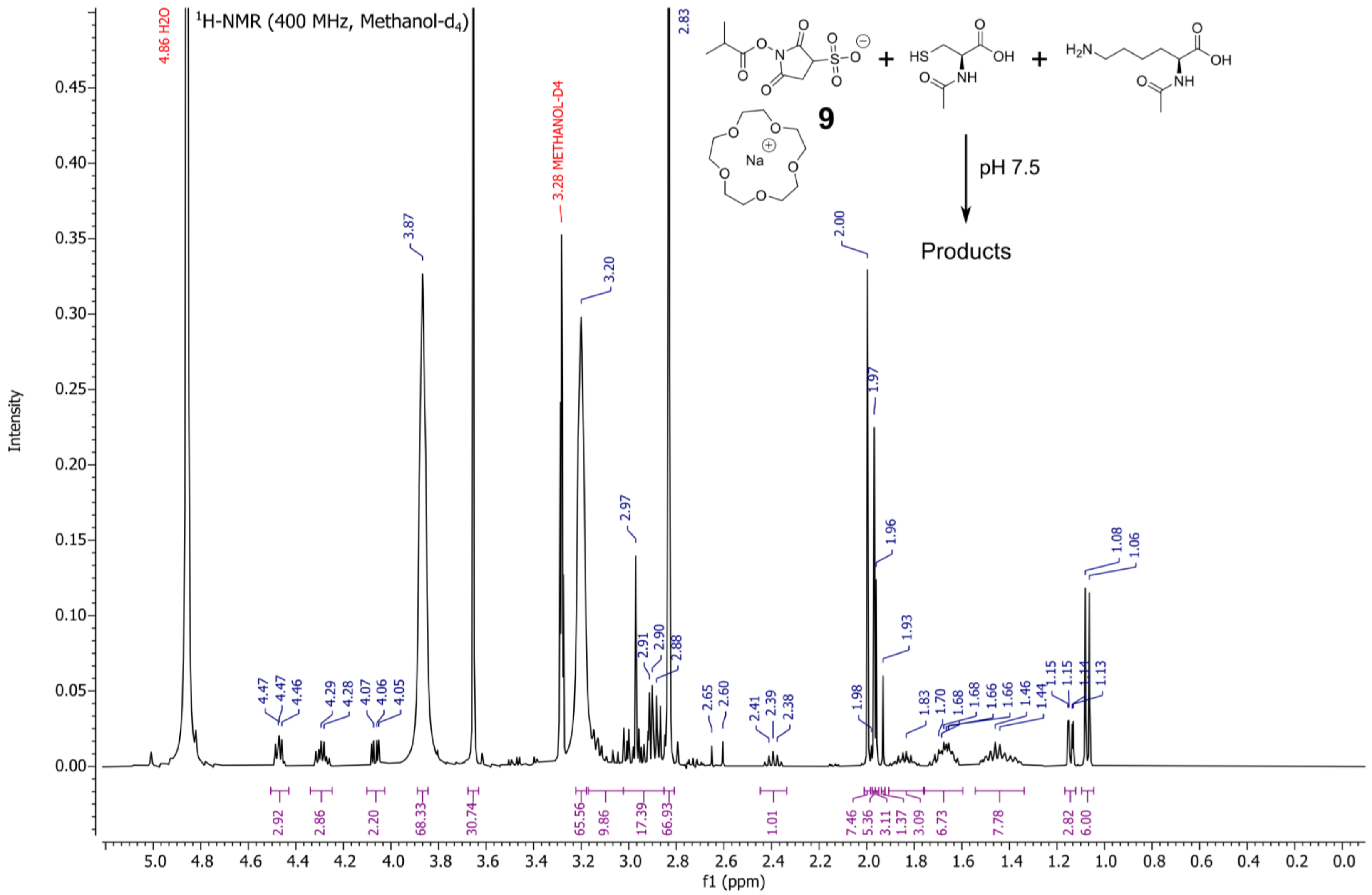

Figure S 91. ¹H-NMR analysis of the product mixture obtained from the reaction C-Sulfo NHS ester **9** with *N*α-Acetyl-L-lysine and *N*-Acetyl-L-cysteine in pH 7.5 NMM buffer (after lyophilization).

LC-MS traces of standard solutions of S3 and S4

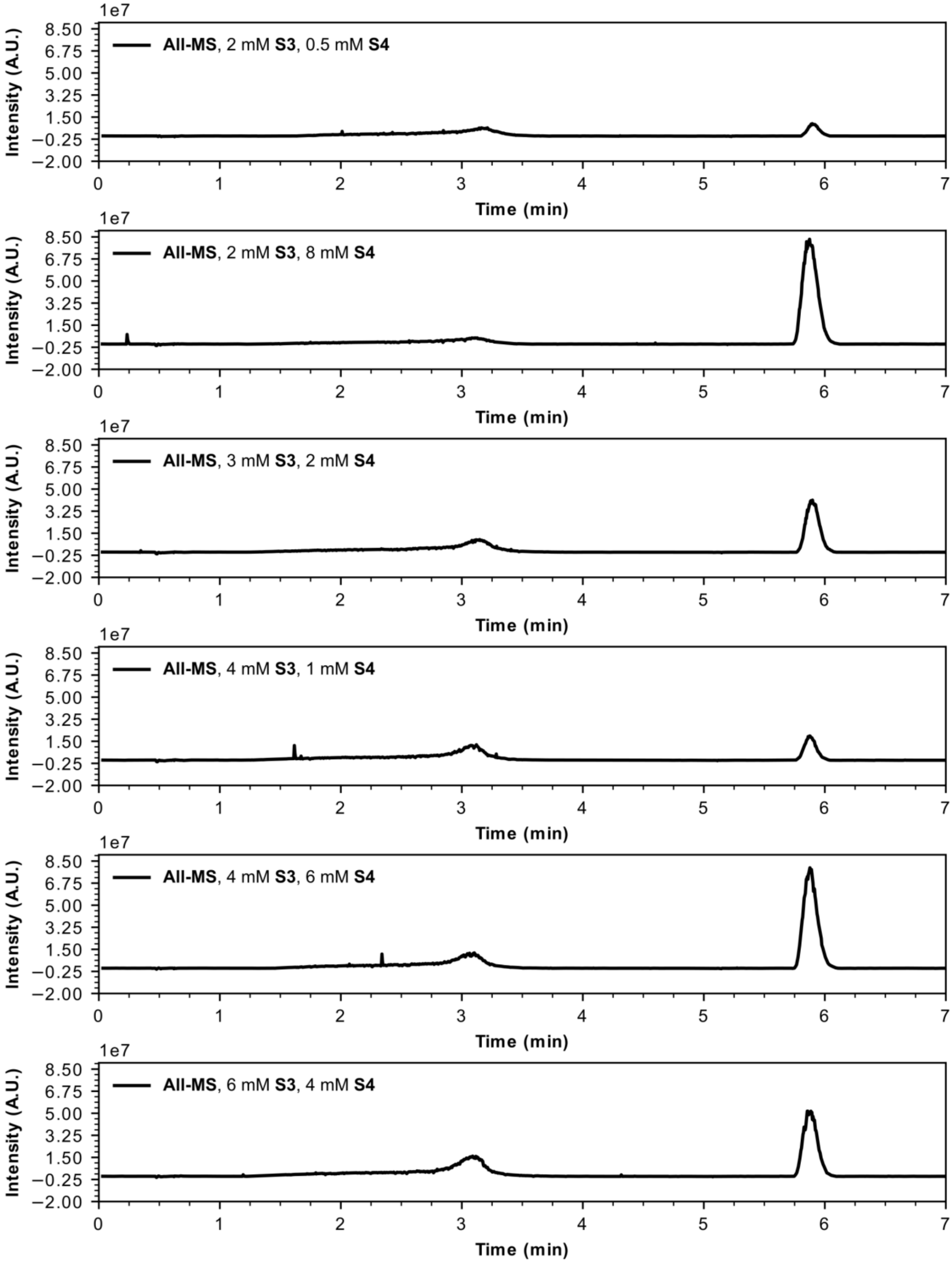

Figure S 92. LC-MS traces of standard solutions containing **S3** and **S4**.

References

(1) Snyder, L. R. Classification of the solvent properties of common liquids. *J. Chromatogr. A.* **1974**, *92*, 223-230.
(2) Bou-Hamdan, F. R.; Lévesque, F.; O'Brien, A. G.; Seeberger, P. H. Continuous flow photolysis of aryl azides: Preparation of 3H-azepinones. *Beilstein J. Org. Chem.* **2011**, *7*, 1124-1129. DOI: 10.3762/bjoc.7.129 PubMed.
(3) Jensen, S. M.; Kimani, F. W.; Jewett, J. C. Light-Activated Triazabutadienes for the Modification of a Viral Surface. *ChemBioChem.* **2016**, *17* (23), 2216-2219. DOI: 10.1002/cbic.201600508 (acccessed 2020/10/22).
(4) Yates, N. D. J.; Hatton, N. E.; Fascione, M. A.; Parkin, A. Site-Selective Aryl Diazonium Installation onto Protein Surfaces at Neutral pH using a Maleimide-Functionalized Triazabutadiene. *ChemBioChem.* **2023**, e202300313. DOI: https://doi.org/10.1002/cbic.202300313 (acccessed 2023/08/08).
(5) Barber, L. J.; Yates, N. D. J.; Fascione, M. A.; Parkin, A.; Hemsworth, G. R.; Genever, P. G.; Spicer, C. D. Selectivity and stability of N-terminal targeting protein modification chemistries. R*SC Chem. Biol.* **2023**, *4* (1), 56-64, 10.1039/D2CB00203E. DOI: 10.1039/D2CB00203E.
